# Excited-State Modulation in Donor-Substituted Multiresonant Thermally Activated Delayed Fluorescence Emitters

**DOI:** 10.1021/acsami.2c02756

**Published:** 2022-05-09

**Authors:** Sen Wu, Wenbo Li, Kou Yoshida, David Hall, Subeesh Madayanad Suresh, Thomas Sayner, Junyi Gong, David Beljonne, Yoann Olivier, Ifor D. W. Samuel, Eli Zysman-Colman

**Affiliations:** † Organic Semiconductor Centre, EaStCHEM School of Chemistry, University of St Andrews, St Andrews, Fife KY16 9ST, United Kingdom; ‡ Organic Semiconductor Centre, SUPA School of Physics and Astronomy, University of St Andrews, St Andrews, Fife KY16 9SS, United Kingdom; § Laboratory for Chemistry of Novel Materials, University of Mons, 7000 Mons, Belgium; ∥ Laboratory for Computational Modeling of Functional Materials & Solid State Physics Laboratory, Namur Institute of Structured Matter, University of Namur, Rue de Bruxelles, 61, 5000 Namur, Belgium

**Keywords:** organic light-emitting diodes, thermally activated delayed fluorescence, multiresonant thermally activated delayed fluorescence, short-range charge transfer, narrowband emission, donor decoration

## Abstract

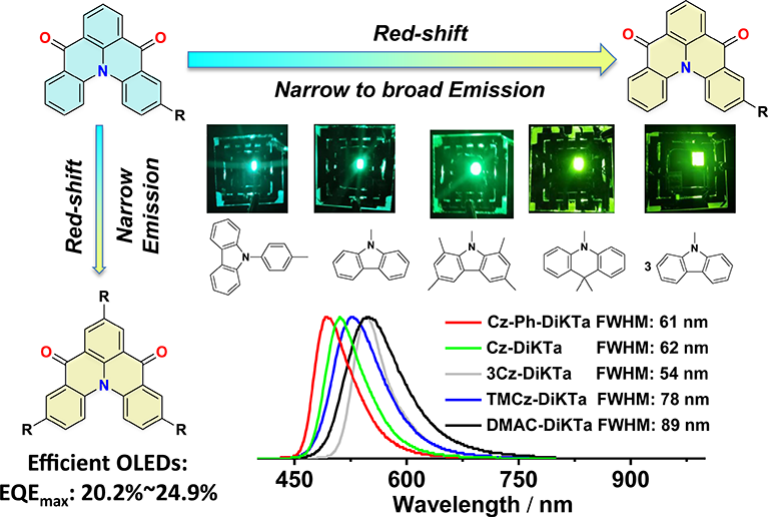

Strategies to tune the emission of multiresonant thermally activated delayed fluorescence (MR-TADF) emitters remain rare. Here, we explore the effect of donor substitution about a MR-TADF core on the emission energy and the nature of the excited state. We decorate different numbers and types of electron-donors about a central MR-TADF core, **DiKTa**. Depending on the identity and number of donor groups, the excited state either remains short-range charge transfer (SRCT) and thus characteristic of an MR-TADF emitter or becomes a long-range charge transfer (LRCT) that is typically observed in donor–acceptor TADF emitters. The impact is that in three examples that emit from a SRCT state, **Cz-DiKTa**, **Cz-Ph-DiKTa**, and **3Cz-DiKTa**, the emission remains narrow, while in four examples that emit via a LRCT state, **TMCz-DiKTa**, **DMAC-DiKTa**, **3TMCz-DiKTa**, and **3DMAC-DiKTa**, the emission broadens significantly. Through this strategy, the organic light-emitting diodes fabricated with the three MR-TADF emitters show maximum electroluminescence emission wavelengths, λ_EL_, of 511, 492, and 547 nm with moderate full width at half-maxima (fwhm) of 62, 61, and 54 nm, respectively. Importantly, each of these devices show high maximum external quantum efficiencies (EQE_max_) of 24.4, 23.0, and 24.4%, which are among the highest reported with ketone-based MR-TADF emitters. OLEDs with D–A type emitters, **DMAC-DiKTa** and **TMCz-DiKTa**, also show high efficiencies, with EQE_max_ of 23.8 and 20.2%, but accompanied by broad emission at λ_EL_ of 549 and 527 nm, respectively. Notably, the **DMAC-DiKTa**-based OLED shows very small efficiency roll-off, and its EQE remains 18.5% at 1000 cd m^–2^. Therefore, this work demonstrates that manipulating the nature and numbers of donor groups decorating a central MR-TADF core is a promising strategy for both red-shifting the emission and improving the performance of the OLEDs.

## Introduction

Thermally activated delayed fluorescence (TADF) materials are a promising class of emitters for organic light-emitting diodes (OLEDs) as the devices can realize up to 100% internal quantum efficiency, while the organic emitters can be easily synthesized at a low cost and are sustainable.^1−3^ TADF operates by converting nonemissive triplet excitons into singlets through endothermic reverse intersystem crossing (RISC). RISC between singlet and triplet excited states is only possible when there is spin–orbit coupling (SOC) between them, and when the energy gap between them (Δ*E*_ST_) is sufficiently small. The magnitude of Δ*E*_ST_ is correlated with the degree of overlap of the orbitals involved in the transition, which typically are the highest occupied molecular orbital (HOMO) and lowest unoccupied molecular orbital (LUMO).^4^ The corresponding molecular design usually involves minimizing the conjugation between the electron-donating moieties and electron-accepting moieties by adopting a strongly twisted conformation between these two units. This commonly used design has a number of drawbacks. Due to the large redistribution of the electron density during the transition, the nature of the emission is charge transfer (CT) between the donor and acceptor.^5^ This, coupled with the conformational flexibility inherent in the design, leads to a broad emission spectrum (full width half-maximum, fwhm, of 80–200 nm).^6^ This significantly degrades the color gamut of the OLEDs, which is an undesirable trait for displays. Therefore, there is at present a growing effort to develop TADF materials that show both a small Δ*E*_ST_ and narrow emission spectra.

One subclass of TADF emitters that responds to these criteria are multiresonant TADF emitters (MR-TADF). Examples of MR-TADF emitters are shown in Figure S3. MR-TADF emitters are nanographenes containing suitably positioned electron-donating atoms (e.g., N, O, and S) and electron-deficient atoms or groups (e.g., B and C=O) within the fused aromatic framework. In these compounds, electrons and holes are localized on adjacent atoms due to the complementary mesomeric effect of the electron-donating and electron-accepting units leading to the required small exchange integral and Δ*E*_ST_.^7^ This electron density distribution is reflected in the small degree of positive solvatochromism that is characteristic of a short-range charge transfer (SRCT) excited state. Although nearly 200 MR-TADF emitters have been reported since the first example in 2016,^8^ the majority show blue or green emission. There are still very few examples that emit at longer wavelengths.^7^ Therefore, this work focuses on developing longer wavelength MR-TADF emitters.

There are several potential strategies that may be employed to tune the emission energy toward the red. The first involves strategic placement of the relative positions of the electron-donating and electron-accepting groups. In 2020, Yasuda and co-workers,^9^ arranged two electron-donating nitrogen atoms para to each other and two boron atoms para to each other about the central phenyl ring (namely, B-π-B and N-π-N), which led to a significantly red-shifted emission and is the first example of a red MR-TADF emitter, **BBCz-R**, showing an emission maximum, λ_PL_, of 615 nm in toluene solution. Duan and co-workers,^10^ adopted a similar strategy, producing two red emitters, **R-BN** and **R-TBN**, with N-π-N arrangements (λ_PL_ = 662 and 692 nm in toluene solution). A second strategy involves modifying the MR-TADF core with electron-donating groups to increase the charge transfer character. Hatakeyama and co-workers^11^ reported a green emitter (λ_PL_ of 506 nm in 1 wt % PMMA film), **OAB-ABP-1**, that contains an extended π-skeleton that consists of an alternating pattern of *para*-disposed O–B–N atoms. Kido and co-workers^12^ reported a green emitter **PXZ-BN** with λ_PL_ of 502 nm by replacement of carbazole for phenoxazine within **BCz-BN** skeleton. In a similar vein, Yang and co-workers incorporated sulfur, affording the green emitter **2PTZBN** (λ_PL_ = 510 nm in toluene), with the expectation of enhancing spin–orbit coupling and hence the reverse intersystem crossing rate.^13^ A third strategy to modulate the emission involves the incorporation of peripheral electron-donating or electron-accepting moieties. Duan and co-workers^14^ reported the first examples of green-emitting MR-TADF emitters (λ_PL_ = 502 nm in 6 wt % doped mCPCB 9-(3-(9*H*-carbazol-9-yl)phenyl)-9*H*-3,9′-bicarbazole (mCPBC) film), **2F-BN**, by decorating peripheral electron-withdrawing fluorophenyl groups para to the central boron atom. Wang and co-workers also employed the same strategy, incorporating electron-withdrawing benzonitrile units in compound **(R)-OBN-4CN-BN**, obtaining a green emitter (λ_PL_ = 500 nm in toluene). Duan and co-workers^15^ reported another green-emitting MR-TADF compound (**AZA-BN**) that incorporates a fused azaphenanthrene (λ_PL_ = 522 nm in toluene). Using the same **BCz-BN** skeleton, Wang and co-workers reported the green emitter (***m*****-Cz-BNCz**) that contains a *meta*-disposed auxiliary di-*tert*-butylcarbazole with respect to the central boron atom (λ_PL_ = 519 nm in toluene).^16^ Recently, Yang and co-workers, introduced donor groups at the *para* position of the carbazole of the **BCz-BN** skeleton and demonstrated color modulation from sky blue to yellow (λ_PL_ of 496 to 562 nm in toluene).^17^ Using the same **BCz-BN** skeleton, You and co-workers combined both donor and acceptor groups located *para* to the N and B atoms, respectively, to realize orange emission (λ_PL_ of 581 nm in toluene).^18^ Although B/N-based emitters have realized full-color emission, their synthesis can only be reached through lithiation–borylation–cyclization reaction or electrophilic fixed-point C–H borylation cyclization reaction, which complicates downstream elaboration of these structures. A second family of MR-TADF compounds employ electron-accepting carbonyl groups in lieu of boron atoms. We showed that decorating the MR-TADF emitter **DiKTa** with mesityl groups, **Mes**_**3**_**DiKTa**, can mitigate undesired aggregation caused quenching (ACQ) and excimer emission while also modestly red-shifting the emission (λ_PL_ = 468 nm in toluene).^19^ We also reported a dimeric compound, **DDiKTa**, consisting of two **DiKTa** units, that showed a red-shifted emission with λ_PL_ of 500 nm.^20^ Liao and co-workers, reported structurally rigid analogs of **DiKTa** that incorporated a carbon-, oxygen-, or sulfur-based tether. The compounds **DQAO**, **OQAO** and **SQAO** showed red-shifted emission compared to that of **DiKTa** with λ_PL_ ranging from 465 to 552 nm in toluene.^21^ Zhang and co-workers, reported the compounds **QAD-Cz**, **QAD-2Cz** and **QAD-mTPDA** that contain donor groups decorating the **DiKTa** core to afford D–A type emitters. These molecules showed blue to red emission, with λ_PL_ in the range of 488–586 nm in toluene solution.^22^

**Figure 1:**
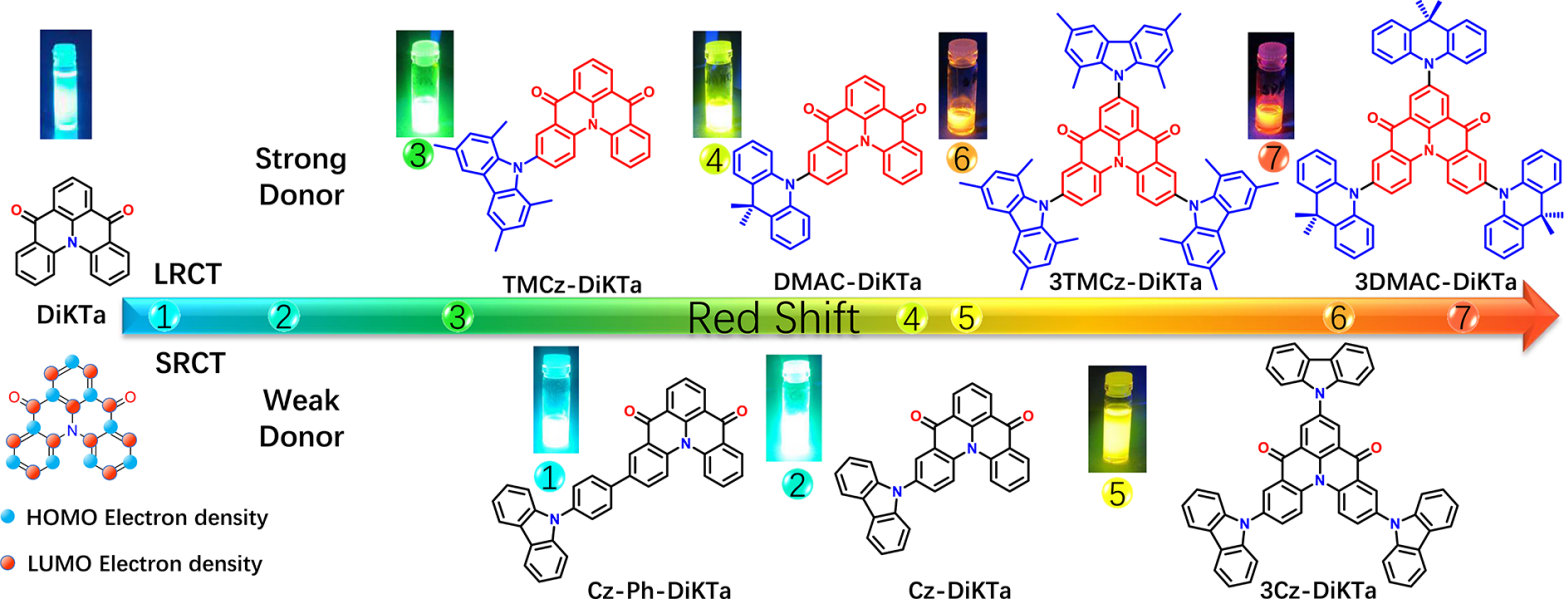
Molecular design of **DiKTa**-based MR-TADF emitters and their emission color. The compounds above the arrow contain stronger donors and emit via a LRCT state and show broad emission, while the compounds below the arrow contain weaker donors and emit via a SRCT state and show narrowband emission. The images are toluene solutions photoexcited at 365 nm.

In this context, we decorated the **DiKTa** core with different numbers of donors with differing electron-donating strengths. These donors include carbazole (Cz), 9,9-dimethylacridan (DMAC), carbazolyl-phenyl (Cz-Ph), and 1,3,6,8-tetramethyl-9*H*-carbazole (TMCz). These donors were positioned *para* to the central electron-donating nitrogen atom. We thus built a framework to systematically study the impact on the emission color and nature of the excited state of the inclusion of these electron-donating groups (Figure 1). It was found that by introducing weak donors such as carbazole to the *para*-carbon position of nitrogen the HOMO levels of the new emitters were significantly destabilized compared to that of parent **DiKTa**, while the LUMO levels were barely perturbed, leading to the desired red-shifted emission. Importantly, the narrow emission characteristic of MR-TADF emitters was maintained. However, when the electron-donating strength was further increased, the emission nature changed to long-range charge transfer (LRCT) and the emission spectra significantly broadened. In this study, weaker-donor-based emitters, **Cz-DiKTa**, **3Cz-DiKTa**, and **Cz-Ph-DiKTa**, maintained their SRCT character in all the tested environments, while stronger-donor-based emitters, **TMCz-DiKTa**, **DMAC-DiKTa**, **3TMCz-DiKTa**, and **3DMAC-DiKTa**, showed a more complicated behavior where LRCT emission dominates in polar media.

## Results and Discussion

### Synthesis

Seven donor-substituted **DiKTa** emitters, **Cz-DiKTa**, **Cz-Ph-DiKTa**, **TMCz-DiKTa**, **DMAC-DiKTa**, **3Cz-DiKTa**, **3TMCz-DiKTa**, and **3DMAC-DiKTa**, were obtained following either palladium-catalyzed Buchwald-Hartwig amination or Suzuki–Miyaura cross-coupling with suitably brominated **DiKTa** intermediates as outlined in Scheme S1. The identity and purity of the seven emitters were ascertained using a combination of ^1^H and ^13^C NMR spectroscopy, HRMS, HPLC and element analysis, and melting point determination.

### Theoretical Studies

The frontier molecular orbitals (FMOs) of these emitters were first modeled based on the optimized ground state gas-phase geometry using density functional theory (DFT) at the PBE0/6-31G(d,p) level of theory. The HOMO and LUMO distributions are shown in Figure S5, and the HOMO and LUMO level of the seven compounds are listed in the Table S1. Compared to **DiKTa** (−5.94/–2.31 eV), the HOMO level is destabilized by 0.21–0.68 eV, and the degree of destabilization correlates with both the strength and numbers of the peripheral donor group. Within the series of the donor-substituted **DiKTa** compounds, we noticed that the occupied orbitals localized on the **DiKTa** core do not correspond to the HOMO, instead, their HOMOs reside on the donor (see Figure S5). For the compounds with the weakest donors (**Cz-DiKTa**, **Cz-Ph-DiKTa**, and **3Cz-DiKTa**) this orbital slightly delocalizes to the carbazoles, thereby resulting in destabilization compared to **DiKTa** while for the compounds containing the strongest donors (**TMCz-DiKTa**, **DMAC-DiKTa**, and **3DMCz-DiKTa**) the energy of the **DiKTa**-localized orbital remains unaffected.

**Figure 2:**
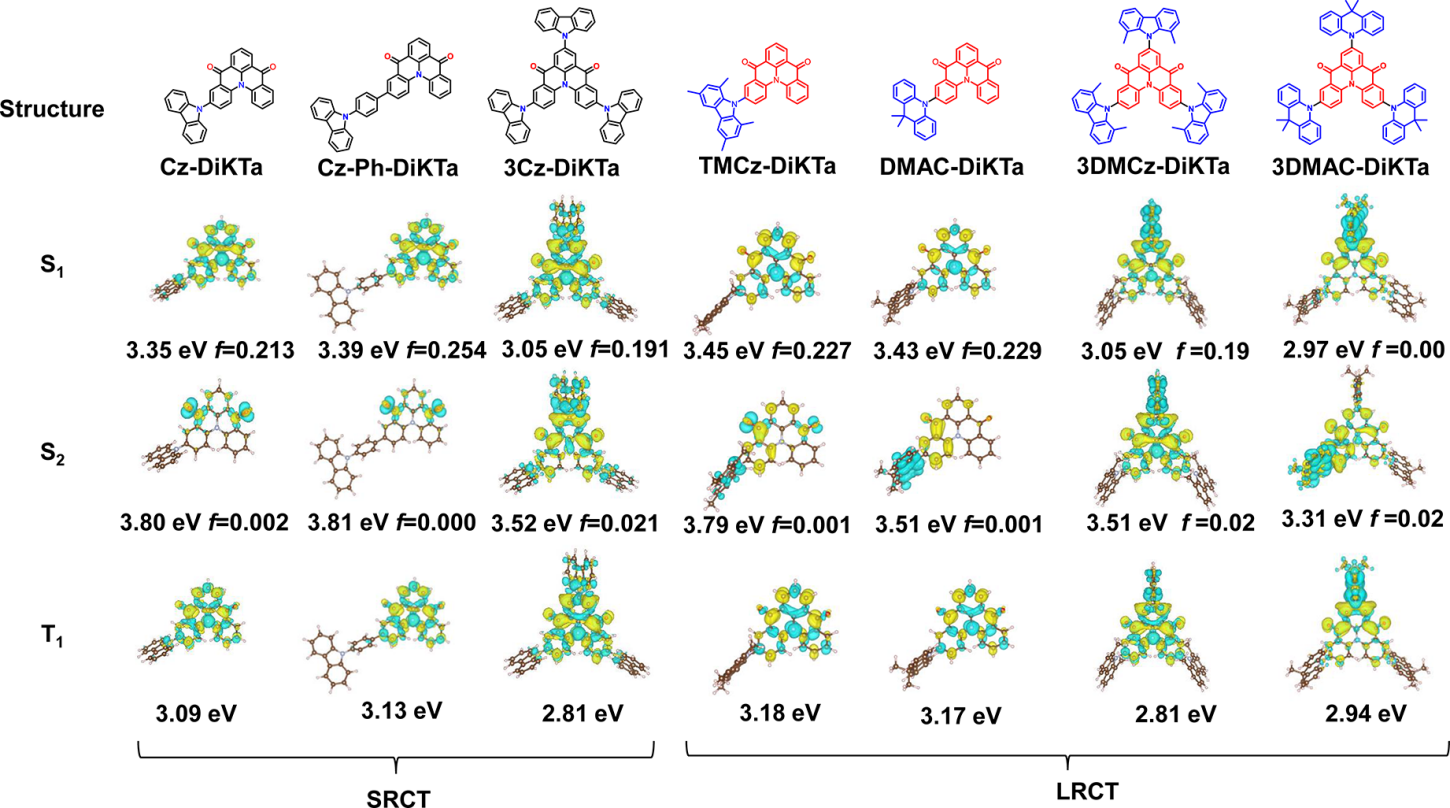
Molecular structures and difference density plots of S_1_ and S_2_ excited states (calculated in the gas phase at the SCS-CC2/cc-pVDZ level) for **Cz-DiKTa**, **Cz-Ph-DiKTa**, **3Cz-DiKTa**, **TMCz-DiKTa**, **DMAC-DiKTa**, **3DMCz-DiKTa**, and **3DMAC-DiKTa**. *f* is the oscillator strength.

We employed spin component scaled second order approximate coupled cluster (SCS-CC2) with the cc-pVDZ basis set to more accurately model the nature of the charge transfer excited states. Figure 2 shows the difference density plots of the S_1_ and the second lowest singlet excited state (S_2_) transitions of these emitters obtained using SCS-CC2. **Cz-DiKTa**, **Cz-Ph-DiKTa**, and **3Cz-DiKTa** all exhibit similar S_1_ difference density plot patterns akin to that for **DiKTa** (shown in Figure S7). On the basis of the charge-transfer distance, *D*_CT_ < 1.4 Å, we assign these excited states to be SRCT (Table S2). At the same time, small contributions to the difference density plots can be seen at the peripheral donor fragments in the new emitters, especially on the “top” carbazole moiety in **3Cz-DiKTa**. The S_2_ state of each of **Cz-DiKTa**, **TMCz-DiKTa**, and **Cz-Ph-DiKTa** possesses n−π* character, while for **3Cz-DiKTa** S_2_ remains a π–π* transition. The S_1_ difference density plot of **DMAC-DiKTa** is almost identical to that of **DiKTa**, while the S_2_ difference density plots show LRCT character as the increased density can be seen on the electron-deficient **DiKTa** core and the decreased density is located on the peripheral DMAC moieties. Indeed, the *D*_CT_ of this state is 3.18 Å, which is characteristic of a LRCT state. Since all the calculations are carried out in gas-phase, potentially the nature of the emissive excited state may switch between SRCT and LRCT, depending on the environment as the energy gap between the S_1_ and S_2_ (Δ*E*_S1S2_) of this emitter is small (0.34 eV) and the S_2_ electrical dipole moment for the S_2_ state is large. We have also carried out the same calculations on other reported donor–acceptor type emitters containing an MR-TADF moiety acting as the acceptor to validate our computational methodology (Figure S4).^23^ All the investigated emitters show SRCT S_1_ states in the gas-phase calculations and a narrow emission characteristic of a MR-TADF behavior. **ADBAN-Me-MesCz** and **DABNA-2** show large Δ*E*_S1S2_ of 0.71 and 0.64 eV at the SCS-CC2 level, which suggests that this Δ*E*_S1S2_ energy gap is large enough to prevent the switching of light emission from the SRCT state to the LRCT state when considering the impact of solvent effects or polarization effects arising in the solid state. By contrast, **PXZ-DABOA**, **TDBAAc**, **TDBA-DI** and **QAO-dic** all have predicted LRCT S_2_ states_,_ and much smaller Δ*E*_S1S2_ values of 0.04–0.29 eV. Such a small energy difference implies that LRCT-SRCT inversion is possible when the medium is sufficiently polar. In the case of **3DMCz-DiKTa** and **3DMAC-DiKTa**, the S_1_ states show a strong contribution of the hole density on the top Cz unit, while the electron density is mainly localized on the **DiKTa** core, resulting in a LRCT state. The calculated S_1_/ T_1_ energies for the MR-TADF predicted emitters, **Cz-DiKTa**, **Cz-Ph-DiKTa** and **3Cz-DiKTa,** are 3.35/3.09, 3.39/3.13 and 3.05/2.81 eV, respectively, with corresponding Δ*E*_ST_ values of 0.26, 0.26, and 0.24 eV. These Δ*E*_ST_ values are of similar magnitude to that of **DiKTa** (0.27 eV), and it indicates a likely similar TADF efficiency in these compounds. As **3TMCz-DiKTa** and **3DMAC-DiKTa** are predicted to be LRCT TADF emitters, the Δ*E*_ST_ obtained from the SCS-CC2 calculations are smaller (0.18 and 0.03 eV, respectively).

### Optoelectronic Properties

**Figure 3:**
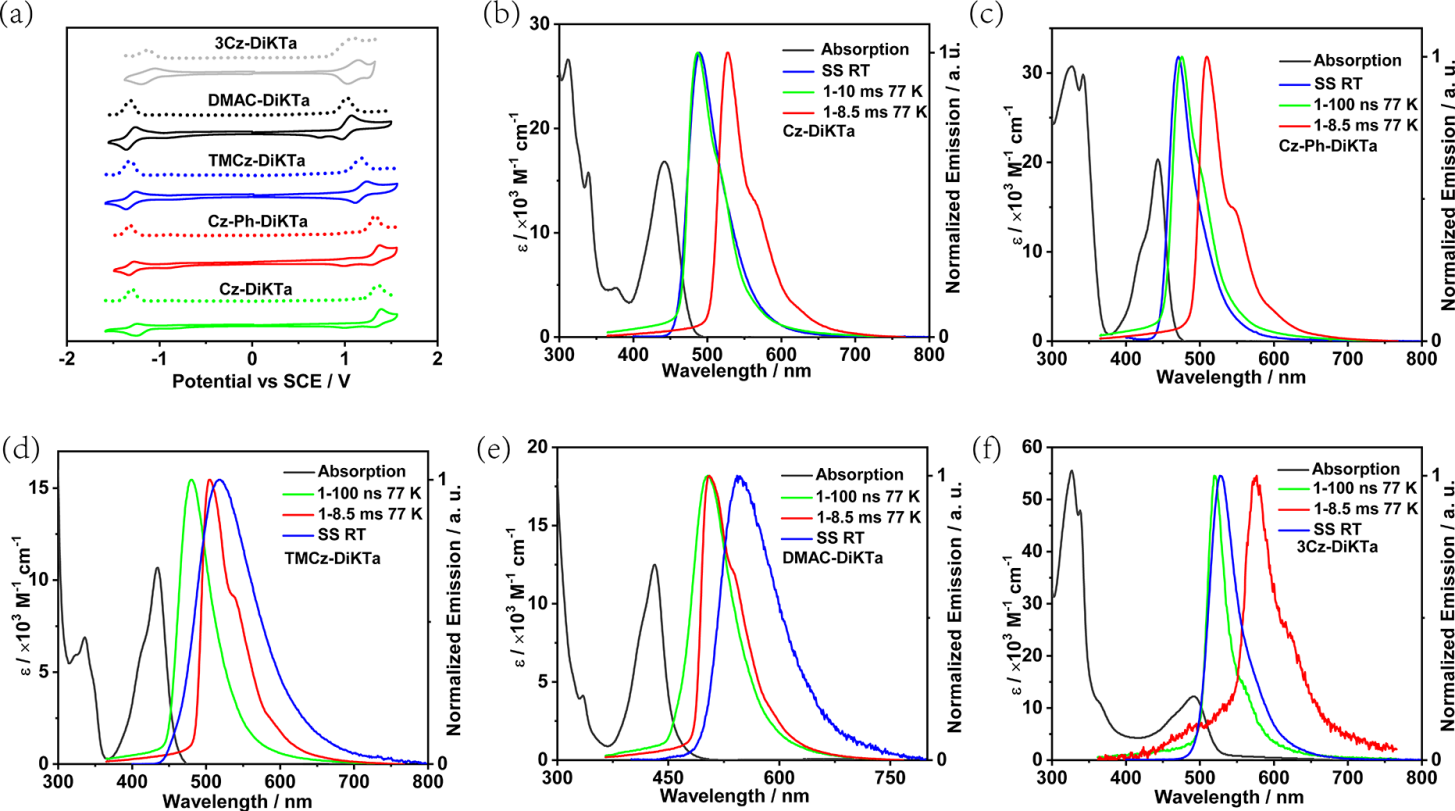
(a) Cyclic voltammogram (CV) and differential pulse voltammetry (DPV) in degassed DCM with 0.1 M [*n*Bu_4_N]PF_6_ as the supporting electrolyte and Fc/Fc^+^ as the internal reference (0.46 V vs SCE).^24^ Absorption and steady-state PL spectra obtained in toluene at RT (SS RT), prompt PL (1–100 ns) and phosphorescence spectra (1–10 ms), obtained in toluene glass at 77 K, measured by iCCD (λ_exc_ = 340 nm) for (b) **Cz-DiKTa**, (c) **Cz-Ph-DiKTa**, (d) **TMCz-DiKTa**, (e) **DMAC-DiKTa**, and (f) **3Cz-DiKTa**.

Cyclic voltammetry (CV) and differential pulse voltammetry (DPV) measurements were used to experimentally determine the HOMO and LUMO levels. The CV and DPV profiles in dichloromethane are shown in Figure 3a (**3TMCz-DikTa** and **3DMAC-Dikta** are shown in Figure S8), and the electrochemical data are summarized in Table S3. The CV profile of **Cz-DiKTa**, **Cz-Ph-DiKTa**, **TMCz-DiKTa**, **DMAC-DiKTa**, **3Cz-DiKTa**, **3TMCz-DiKTa**, and **3DMAC-DiKTa** all show reversible reduction waves, which corresponds to the reduction localized on the **DiKTa** core. While **Cz-DiKTa** and **Cz-Ph-DiKTa** show irreversible oxidation waves, which are assigned to the oxidation of the carbazole, when the donor is DMAC and TMCz, the oxidation waves become significantly more reversible. The *E*_red_/*E*_ox_ values of all seven emitters are determined from the peak of the DPVs. The LUMO levels of **Cz-DiKTa**, **Cz-Ph-DiKTa**, **TMCz-DiKTa**, and **DMAC-DiKTa** are almost identical to that of **DiKTa** (HOMO/LUMO values of −6.12/–3.00 eV). When the numbers of donors are increased (**3Cz-DiKTa**, **3TMCz-DiKTa**, and **3DMAC-DiKTa**), the LUMO stabilizes by ca. 0.2 eV. In comparison, increasing the electron-donating strength of the peripheral donor (e.g., from Cz to DMAC) results in shallower HOMO levels. Similarly, increasing the number of electron donors also results in shallower HOMOs. Therefore, both strategies can be used to reduce the HOMO–LUMO band gap.

The room-temperature ultraviolet–visible (UV–vis) absorption, steady-state photoluminescence (PL), recorded at room-temperature (SS RT) and PL spectra of the prompt and delayed emission recorded at 77 K (the latter being the phosphorescence spectra) in toluene (10^–5^ M) are shown in Figure 3b–f, and Figure S9 shows the PL spectra for **3TMCz-DiKTa** and **3DMAC-DiKta**. The corresponding data are summarized in Table S4. The absorption spectra all show two characteristic absorption bands. The higher energy bands (300–430 nm) are attributed to π–π* locally excited (LE) transitions of both the donors and the **DiKTa** core, and the lower energy bands between 443 and 492 nm are attributed to SRCT transitions centered on the **DiKTa** core that are characteristic of MR-TADF emitters. Compared to **DiKTa**,^19^ the lowest-energy absorption band of the **Cz-DiKTa**, **Cz-Ph-DiKTa** and **3Cz-DiKTa** progressively red-shifts and becomes broader and weaker as shown in Figure 3, suggesting the increasing CT character of the transitions associated with this band. The CT absorption bands of **TMCz-DiKTa** and **DMAc-DiKTa** have lower molar absorptivities, which can be rationalized by the small FMO overlap due to the strongly twisted conformation of the bulky TMCz donor and the electron-donating strength of the DMAC donor. The seven compounds show a progressively red-shifted emission with increasing number and strength of the electron donors from 472 to 667 nm compared with those of **DiKTa** (λ_PL_ = 460 nm, fwhm = 27 nm). **Cz-DiKTa**, **Cz-Ph-DiKTa**, and **3Cz-DiKTa** show small fwhm values of 54, 47, and 53 nm, respectively. The small fwhm is correlated to the small Stokes shifts of 31–50 nm, which indicates that the structural relaxation is small in their excited states. In contrast, the Stokes shifts of **TMCz-DiKTa**, **DMAC-DiKTa**, **3TMCz-DiKTa**, and **3DMAC-DiKTa** are much larger (86–242 nm), and the fwhm values are greater than 80 nm. These results indicate that in the presence of strong electron-donating groups, the SRCT character of the excited state disappears, and the long-range D–A type CT character starts to be dominant. These observations agree with the computational results shown in the Figure 2. We next probed how the nature of the emissive excited state evolves as a function of solvent polarity, and the results are presented in Figure S10. **Cz-DiKTa**, **Cz-Ph-DiKTa**, and **3Cz-DiKTa** all show a small degree of positive solvatochromism that is characteristic of SRCT states associated with MR-TADF emitters. By contrast, **TMCz-DiKTa**, **DMAC-DiKTa**, **3TMCz-DiKTa**, and **3DMAC-DiKTa** show significant positive solvatochromism, suggesting that the lowest excited states of these compounds, especially in polar media, are LRCT in nature. They also show LRCT and SRCT dual emission in high polarity solvents, with emission moving from SRCT to LRCT with increasing polarity (Figure S10**)**. This was observed previously using a MR-TADF core (**ADBNA-Me-Mes**), with NMe_2_ substitution.^19^

The Δ*E*_ST_ values were determined from the difference in energy of the onsets of the prompt fluorescence and phosphorescence spectra in toluene at 77 K. The corresponding Δ*E*_ST_ values of **Cz-DiKTa**, **Cz-Ph-DiKTa**, **TMCz-DiKTa**, **DMAC-DiKTa**, **3Cz-DiKTa**, **3TMCz-DiKTa**, and **3DMAC-DiKTa** are 0.20, 0.22, 0.23, 0.21, 0.11, 0.01, and 0.01 eV, respectively. The Δ*E*_ST_ values of **Cz-DiKTa**, **Cz-Ph-DiKTa**, and **3Cz-DiKTa** are consistent with the SCS-CC2 calculations, which implies the emissive excited state is SRCT in nature. **TMCz-DiKTa** shows a broader emission spectrum but possesses an identical Δ*E*_ST_ value to the predicted one, which indicates that there may be mixed SRCT/LRCT character in low polarity solvents such as toluene. The calculated D–A type emitters **DMAC-DiKTa**, **3TMCz-DiKTa**, and **3DMAC-DiKTa** show much smaller Δ*E*_ST_ values, which reflects the smaller overlap integral.

We next evaluated the photophysical properties of the seven emitters in drop-cast 1,3-bis(*N*-carbazolyl)benzene (mCP) films at a doping concentration of 2 wt % (Table 1). This host was chosen due to its high triplet energy of 2.81 eV,^25^ and the photoluminescence quantum yield (Φ_PL_) was found to be the highest at a concentration of 2 wt % (Tables S5 and S6). Indeed, **Cz-DiKTa**, **Cz-Ph-DiKTa**, **TMCz-DiKTa**, **DMAC-DiKTa**, and **3Cz-DiKTa** films show high Φ_PL_ values of 90, 77, 71, 72, and 78%, respectively. However, **3TMCz-DiKTa** and **3DMAC-DiKTa** only present Φ_PL_ values of around 20%, which may be attributed to the much stronger CT band and relatively lower calculated oscillator strength for the CT states (*vide supra*). Furthermore, their emission is red-shifted compared to the others, resulting in larger nonradiative decay processes. S_1_ and T_1_ levels in the doped film were determined from the onsets of the prompt fluorescence and phosphorescence spectra, respectively, measured at 77 K (Figure S11). The corresponding Δ*E*_ST_ values of **Cz-DiKTa**, **Cz-Ph-DiKTa**, and **3Cz-DiKTa** are 0.14, 0.10, and 0.16 eV, respectively, which are slightly smaller than those measured in toluene glass. A possible explanation for the smaller Δ*E*_ST_ in doped film can be attributed to the changes of conformation upon slow cooling of the film in comparison to flash freezing of the toluene glass samples, as well as host/guest interaction.^26^**DMAC-DiKTa**, **TMCz-DiKTa**, **3TMCz-DiKTa**, and **3DMAC-DiKTa** possess very small Δ*E*_ST_ values ranging from 0.01 to 0.08 eV_._

**Table 1:** Photophysical Data in 2 wt % Doped mCP Films

compound	λ_PL_ (nm)^a^	fwhm (nm)^b^	Φ_PL_ (%)^c^	τ_p_, τ_d_ (ns, μs)^d^	*T*_1_ (eV)^e^	*S*_1_ (eV)^f^	Δ*E*_ST_ (eV)^g^
**DiKTa**	466	40	70	4.5, 168	2.55	2.75	0.20
**Cz-DiKTa**	502	54	90	8.6, 196	2.48	2.62	0.14
**Cz-Ph-DiKTa**	486	47	77	6.2, 153	2.53	2.63	0.10
**TMCz-DiKTa**	501	80	71	8.2, 22	2.56	2.64	0.08
**DMAc-DiKTa**	534	94	76	26.4, 6.6	2.61	2.57	0.04
**3Cz-DiKTa**	539	53	78	11.2, 286	2.25	2.41	0.16
**3TMCz-DiKTa**	577	110	18	29.1, 3.0	2.38	2.39	0.01
**3DMAc-DiKTa**	599	116	23	29.9, 3.5	2.32	2.33	0.01

a Obtained at 300 K, λ_exc_ = 340 nm.

b Full width at half-maximum.

c Calculated using an integrating sphere, under N_2_ at λ_exc_ = 340 nm; Φ_PL_ values are ±10% of the stated value (e.g., 70 ± 7% for **DiKTa**).

d Measured at λ_exc_ = 379 nm and 300 K under vacuum.

e Obtained from the onset of the delayed spectrum (1–10 ms) at 77 K, λ_exc_ = 343 nm.

f Obtained from the onset of the prompt spectrum (1–100 ns) at 77 K.

g Δ*E*_ST_ = *E*(*S*_1_) – *E*(*T*_1_).

**Figure 4:**
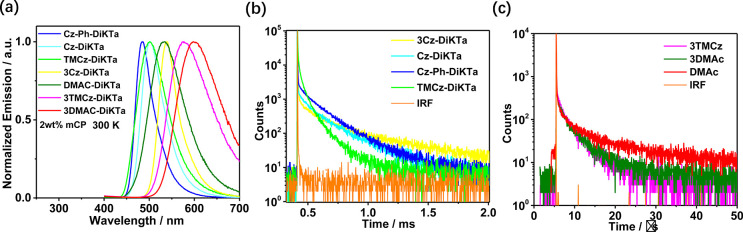
(a) PL spectra of **Cz-DiKTa**, **Cz-Ph-DiKTa**, **3Cz-DiKTa**, **TMCz-DiKTa**, **Cz-DiKTa**, **3TMCz-DiKTa**, and **3DMAC-DiKTa** in 2 wt % doped mCP films at room temperature, λ_exc_ = 340 nm. (b) Time-resolved PL decays of **Cz-DiKTa**, **Cz-Ph-DiKTa**, **3Cz-DiKTa**, and **TMCz-DiKTa** in 2 wt % mCP at room temperature, λ_exc_ = 379 nm. (c) Time-resolved PL decays curves of **DMAC-DiKTa**, **3TMCz-DiKTa**, and **3DMAC-DiKTa** in 2 wt % doped mCP films at room temperature for λ_exc_ = 379 nm. IRF is the instrument response function.

Figure 4b,c show the time-resolved PL decays of the 2 wt % doped mCP films. All PL decays show prompt and delayed emission components at room temperature. For **Cz-DiKTa**, **Cz-Ph-DiKTa**, and **3Cz-DiKTa**, the prompt emission lifetimes, τ_p_, are in the range of 6.2–11.2 ns, and the delayed emission lifetimes, τ_d_, are between 153 and 286 μs, which are of the same order of magnitude as that of parent compound **DiKTa** (168 μs) in 2 wt % mCP-doped film. Compound **TMCz-DiKTa** shows an intermediate delayed lifetime of 22 μs, indicating that this compound possesses an excited state of intermediate character between LRCT and SRCT in mCP. By contrast, **DMAC-DiKTa**, **3TMCz-DiKTa**, and **3DMAc-DiKTa** show longer prompt emission lifetimes ranging from 26.4 to 29.9 ns and shorter delayed emission lifetimes of 3.0–6.6 μs. The relatively shorter delayed lifetime of these three emitters can be attributed to their smaller Δ*E*_ST_ values. The temperature-dependent time-resolved PL decays of all seven emitters (Figure S13) reveal the expected increase in the contribution of the delayed emission component with increasing temperature, which corroborates the TADF nature of these compounds.

### Organic Light-Emitting Diodes

**Figure 5:**
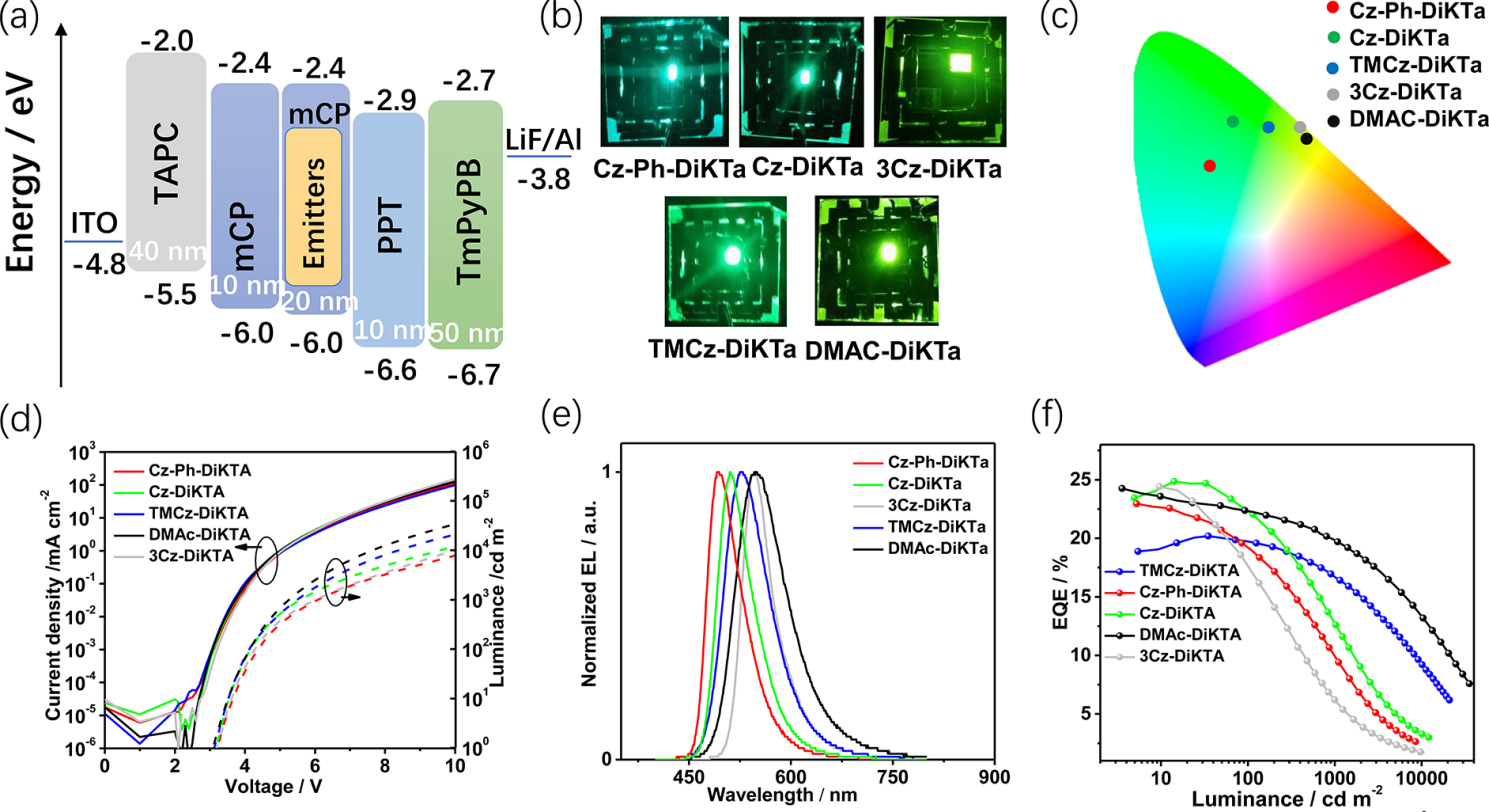
(a) Device configuration with energy levels and thicknesses for each layer. (b) Photographs of OLEDs under operation. (c) Commission Internationale de L’Éclairage diagram. (d) Current density versus voltage and luminance versus voltage curves. (e) Electroluminescence spectra. (f) External quantum efficiency (EQE) versus luminance curves.

We next fabricated vacuum-deposited OLEDs only with **Cz-DiKTa**, **Cz-Ph-DiKTa**, **TMCz-DiKTa**, **DMAC-DiKTa**, and **3Cz-DiKTa**, as these compounds possessed suitably attractive Φ_PL_. Here we utilized a higher concentration of emitter (7.5 wt %) with the aim of improving the charge balance in the OLED device structure; we note that the photophysical behavior of the evaporated 7.5 wt % doped films in mCP is quite similar to that of the 2 wt % doped films in mCP, with only a small red-shift in the emission and a small decrease in Φ_PL_ (Figure S15 and Table S7). The optimized OLED structure is shown in Figure 5a and consists of indium–tin oxide (ITO)/4,4′-cyclohexylidenebis[*N*,*N*-bis(4-methylphenyl)benzenamine] (TAPC, 40 nm)/mCP (10 nm)/7.5 wt % emitter/mCP (20 nm)/2,8-bis(diphenyl-phosphoryl)-dibenzo[*b*,*d*]thiophene (PPT, 10 nm)/1,3,5-tri(*m*-pyridin-3-ylphenyl)benzene (TmPyPb, 50 nm)/LiF (1 nm)/Al (100 nm), where ITO acts as the transparent anode, TAPC acts as the hole transporting layer, mCP acts as both the electron blocking layer and the host within the emissive layer, PPT acts as the hole blocking layer, TmPyPb acts as the electron transporting layer, and LiF acts as the electron injection layer.

Figure 5e illustrates the EL spectra of these devices. The OLEDs with **Cz-DiKTa**, **Cz-Ph-DiKTa**, **TMCz-DiKTa**, **DMAC-DiKTa**, and **3Cz-DiKTa** show electroluminescence maxima, λ_EL_, of 511, 492, 527, 549, and 547 nm, with corresponding Commission Internationale de l’Éclairage (CIE) coordinates of (0.24, 0.61), (0.18, 0.50), (0.32, 0.60), (0.40, 0.57), and (0.39, 0.60), and the comparison between EL and PL of the doped films are shown in Figure S16. The devices based on the MR-TADF emitters, **Cz-DiKTa**, **Cz-Ph-DiKTa**, and **3Cz-DiKTa**, show narrow electroluminescence spectra with fwhm values of 62, 61, and 54 nm which are slightly broader than that of **DiKTa** (39 nm).^19^ The fwhm values are larger for the devices with **TMCz-DiKTa** and **DMAC-DiKTa** (78–89 nm), respectively. This reflects that the emission originates from a LRCT state in these devices in line with that observed in the PL spectra. Overall, the EL emission of the devices can be tuned from sky blue to yellow green by regulating the number and the electron-donating strength of the peripheral donor around the **DiKTa** core.

As depicted in Figure 5f and summarized in Table 2, the devices with **Cz-DiKTa**, **Cz-Ph-DiKTa**, **TMCz-DiKTa**, **DMAc-DiKTa**, and **3Cz-DiKTa** show very high EQE_max_ of 24.9, 23.0, 20.2, 23.8, and 24.4%, respectively, which is much higher than that of the **DiKTa**-based device with **DiKTa** (14.7%) reported by our group.^19^ These EQE_max_ values are among the highest reported for ketone-containing MR-TADF OLEDs (Table S8). Considering the measured Φ_PL_ of the doped films fabricated by thermal evaporation (see Table S9) and assuming 25% outcoupling efficiency, the EQE_max_ of these devices are expected to be, respectively, 19.3, 17.3, 14.3, 15.5, and 18.5%, which are much lower than the observed EQE_max_. One potential explanation would be if the transition dipole of the emitters were horizontally oriented parallel to the substrate surface, as this could lead to higher amount of the out-coupled light from the OLEDs to air.^2^ We therefore measured the molecular orientations of evaporated doped films of the emitters, which are identical to the ones used in the OLEDs. The angle-dependent PL measurement results of these films are shown in Figure S19, and the anisotropy factors, *a*, extracted from the p-polarized emission were found to be 0.33 for **Cz-DiKTa**, 0.36 for **Cz-Ph-DiKTa**, 0.37 for **TMCz-DiKTa**, 0.36 for **DMAc-DiKTa**, and 0.30 for **3Cz-DiKTa**. These values are very close to the anisotropy factors of isotropically oriented emitters (0.33). Considering the device structure and the measured anisotropy factors, the corresponding simulated outcoupling efficiencies of these devices are, respectively, 25.4, 24.4, 23.2, 23.0, and 25.4% (see the Supporting Information for the details of the calculation and Table S11). Combining the measured Φ_PL_ of the films and the simulated outcoupling efficiencies, the EQE_max_ values were expected to be 19.6, 16.8, 13.1, 14.3, and 18.8%, respectively, which are lower than the observed values. Therefore, the emitter orientation alone cannot explain this discrepancy. Similar higher than expected performance was also found in many other MR-TADF emitters.^22,27,28^ Among these works, a group of structurally related emitters, **QAD-Cz**, **QAD-2Cz**, and **QAD-mTDPA**, reported recently by Zhang and co-workers, showed near unity Φ_PL_ values of 99.6, 99.5, and 97.2% in mCP doped film, respectively, and EQE_max_ of the corresponding OLEDs of 20.3, 27.3, and 23.9%, respectively, which implies outcoupling efficiencies of 20.4, 27.4, and 27.1%, respectively.^22^ These EQE_max_ values are also higher than 20%. Unfortunately, the anisotropy factors of these materials were not measured in the paper and the out-coupling efficiency was not discussed.

**Table 2:** Summary of OLED Performance

emitters	λ_EL_ (nm)	fwhm (nm)^a^	*V*_on_ (V)^b^	*L*_max_ (cd m^–2^)^c^	EQE (% max/100/1000)^d^	PE_max_ (lm W^–1^)^e^	CIE^f^
**Cz-DiKTa**	511	62	3.2	13260	24.9/20.4/13.0	68.9	0.24, 0.61
**Cz-Ph-DiKTa**	492	61	3.2	8529	23.0/19.3/10.2	52.3	0.18, 0.50
**TMCz-DiKTa**	527	78	3.1	21758	20.2/19.6/16.7	60.0	0.32, 0.60
**DMAC-DiKTa**	549	89	3.1	35506	23.8/22.3/19.9	78.6	0.40, 0.57
**3Cz-DiKTa**	547	54	3.2	10796	24.4/17.3/6.2	83.1	0.39, 0.60

a Full width at half-maximum of the EL spectrum.

b Turn-on voltage at the luminance of 1 cd m^–2^.

c Maximum luminance.

d Maximum external quantum efficiency/EQE at 100 cd m^–2^/EQE at 1000 cd m^–2^.

e Maximum power efficiency.

f Commission Internationale de L’Éclairage coordinates.

Although the origin of our higher-than-expected EQE_max_ is not clear, we can envisage two potential causes (see the section “Out-coupling efficiency simulation and possible explanation about higher experimental EQE than predicted EQE” in the Supporting Information). The first is that the emission efficiency of our emitters might be underestimated due to some oxygen remaining in the integrating sphere during our measurements of the Φ_PL_; also, in the OLED stack, emission efficiency can be enhanced by the Purcell effect. The second potential explanation could be the microcavity effects in the OLED stack leading to light emission that is directed forward more than for a Lambertian emitter and hence increasing the apparent EQE when measured in the forward direction. As the main focus of this work is to demonstrate the impact of donor substitution about **DiKTa**-type MR-TADF compounds and how it modulates the nature of the CT character of the emitters and affects the performance of the OLEDs, the origin of the apparently high out-coupling efficiency will be investigated in future work.

In addition to the high EQE_max_, these devices also show suppressed efficiency roll-off. The EQE values at 100 cd m^–2^ (EQE_100_) for the **Cz-DiKTa**, **Cz-Ph-DiKTa** and **3Cz-DiKTa** devices are 22.5, 19.2, and 17.8%, respectively, corresponding to an efficiency roll-off of 9.6, 16.5, and 27.0%, respectively. This performance is improved compared to the **DiKTa**-based OLED (44% reported by us^19^ and 54% reported by Liao and co-workers).^27^ The EQE_1000_ values, however, drop dramatically with efficiency roll-off of between 50–74%, which is not uncommon in MR-TADF-based OLEDs such as **Mes**_**3**_**DiKTa** and **DABNA-1**.^8,19^ Serious efficiency roll-off also was observed in the devices with **QAD-Cz**, **QAD-2Cz**, and **QAD-mTDPA**, where the EQE dropped to 0.73, 12.4, and 4.7% at 1000 cd/m^2^, representing an efficiency roll-off greater than 55%. By contrast, for the devices with our D–A type TADF emitters, the EQE_1000_ for the OLEDs based on **DMAC-DiKTa** and **TMCz-DiKTa** are 19.9 and 16.7%, these show a much smaller efficiency roll-off of 16.4 and 17.3%, which can be attributed to the smaller Δ*E*_ST_ values. The OLED using the previously reported D–A emitter, **QAO–DAd**, and similar device structure also showed a comparable efficiency roll-off of 19.2% at 1000 cd/m^2^ (EQE_max_ of 23.9% and EQE_1000_ of 19.3%). Due to the small efficiency roll-off observed in the **DMAC-DiKTa** device, a very high maximum brightness, *L*_max_, of 35 500 cd m^–2^ was reached (Figure 5c). The device performances reported in the present study are among the best results in ketone-containing MR-TADF devices (and devices containing a ketone-containing MR-TADF core as the acceptor in D–A emitters). Moreover, we demonstrate the importance of the choice of peripheral donor in order to maintain the MR-TADF character of the emitters.

## Conclusions

In summary, through attaching different numbers of donors with different electron-donating strengths at the para position to the central nitrogen atom of the previous reported **DiKTa** core, the character of the charge transfer excited state can be modulated from SRCT to LRCT. This change in the nature of the emissive excited state is reflected in a broadening and a bathochromic shift of the emission. The photophysical properties, corroborated by SCS-CC2 calculations, show that the introduction of strongly electron-donating donor moieties to the periphery of the **DiKTa** core leads to a destabilization of the HOMO and an enhancement of the long-range CT character of the emitters. It is noteworthy that the SRCT character that is emblematic of MR-TADF compounds is conserved with the introduction of weak donors (Cz, Cz–Ph), so the color purity of these emitters is high. As a result, we achieved narrowband emission beyond 547 nm (fwhm = 54 nm) in the OLED accompanied by a high EQE_max_ of 24.4% from the device with **3Cz-DiKTa**. The **Cz-DiKTa** OLED exhibited the highest EQE_max_ of 24.9% at λ_EL_ of 511 nm. The OLED with D–A type emitter **3DMAC-DiKTa** showed high EQE_max_ of 24.3% and a small roll-off of 18.5% at 1000 cd m^–2^. The strategy of judiciously decorating the MR-TADF core with weak donating groups is a useful tool to modulate the photophysical properties of these emitters and to realize high-performance OLEDs. However, too strong a choice of donor leads to the generation of donor–acceptor compounds, which leads to red-shifted and broadened emission in the device.

## Experimental Section

### General Method

HPLC analysis was conducted on a Shimadzu LC-40 HPLC system. HPLC traces were carried out using a Shim-pack GIST 3 μm C18 reverse-phase analytical column. Melting points were measured using open-ended capillaries on an Electrothermal 1101D Mel-Temp apparatus and are uncorrected. High-resolution mass spectrometry (HRMS) was carried out at the BBSRC Mass Spectrometry Facility, University of St Andrews. Elemental analyses were carried out by Joe Casillo at the University of Edinburgh.

**Br**_**3**_**DiKTa** and **BrDiKTa** were synthesized following a previously reported protocol.^19,20^

#### 3,7,11-Tri(9*H*-carbazol-9-yl)quinolino[3,2,1-*de*]acridine-5,9-dione (**3Cz-DiKTa**)

**Br**_**3**_**DiKTa** (267.0 mg, 0.5 mmol 1 equiv), carbazole (501.0 mg, 3 mmol, 6 equiv), Pd_2_(dba)_3_ (91.6 mg, 0.1 mmol, 0.2 equiv), tri-*tert*-butylphosphonium tetrafluoroborate (91.5 mg, 0.2 mmol, 0.4 equiv), sodium *tert*-butoxide (288.3 mg, 3 mmol, 6 equiv), and 15 mL of dry toluene were added to a 50 mL Schlenk tube, which was then placed under a nitrogen atmosphere. The solution was heated to reflux and stirred for 24 h. The reaction mixture was brought to room temperature and combined with 50 mL of DCM. The organic phase was collected and washed with brine (3 × 50 mL). The red, floating solid in the aqueous phase was washed with DCM. The organic fractions were combined, and the solvent was evaporated under reduced pressure. The concentrated mixture was filtered and washed with hexane to afford red solid. Then the pure red solid recrystallized from THF and methanol. Yield: 40% (160.0 mg). *R*_f_: 0.45 (EtOAc/hexane = 1:5). Mp: 376–377 °C. ^1^H NMR (400 MHz, CDCl_3_) δ 9.20 (s, 2H), 8.86 (d, *J* = 2.5 Hz, 2H), 8.70 (d, *J* = 9.1 Hz, 2H), 8.29–8.18 (m, 8H), 7.60 (d, *J* = 8.2 Hz, 4H), 7.56–7.49 (m, 8H), 7.41 (t, *J* = 7.4 Hz, 6H). ^13^C NMR (126 MHz, CDCl_3_) δ 140.27, 138.10, 137.42, 136.60, 135.55, 132.84, 132.53, 127.03, 126.61, 126.49, 125.46, 124.41,124.06, 123.98, 122.65, 121.29, 121.07, 120.69, 109.22, 108.85. HRMS (ESI-MS): [C_56_H_32_N_4_O_2_ + Na]^**+**^ Calcd: 815.25. Found: 815.2415. HPLC analysis, 96.2% pure, retention time 11.3 min in 98% methanol, 2% water. Calcd for C_65_H_50_N_4_O_2_: C, 84.83%, H, 4.07%, and N, 7.07%. Anal. C, 84.54%, H, 4.16%, and N, 6.34%.

#### 3,7,11-tris(9,9-dimethylacridin-10(9H)-yl)quinolino[3,2,1-de]acridine-5,9-dione (**3DMAC-DiKTa**)

The procedure is similar to that used for **3Cz-DiKTa** with DMAC (502.0 mg, 2.40 mmol, 6 equiv) used in lieu of carbazole. An orange-red powder was obtained after the crude product was purified via column chromatography (EtOAc/hexane = 1:10). Yield: 86% (315.0 mg). *R*_f_: 0.25 (EtOAc/hexane = 1:10). Mp: 392–393 °C. ^1^H NMR (500 MHz, CDCl_3_) δ 8.87 (s, 2H), 8.61 (dd, *J* = 5.6, 4.6 Hz, 4H), 7.84 (dd, *J* = 8.9, 2.5 Hz, 2H), 7.54 (ddd, *J* = 6.2, 4.2, 1.8 Hz, 6H), 7.09–6.94 (m, 12H), 6.41 (dd, *J* = 7.9, 1.5 Hz, 4H), 6.34–6.27 (m, 2H), 1.77 (d, *J* = 2.5 Hz, 18H). ^**13**^C NMR (125 MHz, CDCl_3_) δ 177.01, 140.58, 139.01, 138.69, 137.93, 136.93, 136.68, 131.19, 130.74, 128.63, 126.50, 126.47, 126.22, 125.48, 123.17, 121.38, 121.34, 114.21, 114.09, 36.13, 30.98. HRMS (ESI-MS): [C_65_H_50_N_4_O_2_ + H]^+^ Calcd: 919.39. Found: 919.3922. HPLC analysis, 95.6% pure, retention time 11.9 min in 98% methanol, 2% water. Calcd for C_65_H_50_N_4_O_2_: C, 84.94%; H, 5.48%, and N, 6.10%. Anal. C, 84.21%, H, 5.23%, and N, 5.90%.

#### 3,7,11-Tris(1,3,6,8-tetramethyl-9*H*-carbazol-9-yl)quinolino[3,2,1-*de*]acridine-5,9-dione (**3TMCz-DiKTa**)

The procedure is similar to that used for **3Cz-DiKTa** with tetramethylcarbazole (352.0 mg, 1.58 mmol, 4.5 equiv) used in lieu of carbazole. An orange-yellow powder was obtained after the raw product was purified via column chromatography (EtOAc/hexane = 1:8). Yield: 55% (186 mg). *R*_f_: 0.30 (EtOAc/hexane = 1:8). Mp: 378–379 °C. ^1^H NMR (400 MHz, CDCl_3_) δ 8.85 (s, 2H), 8.66 (d, *J* = 2.5 Hz, 2H), 8.31 (d, *J* = 8.9 Hz, 2H), 7.88 (dd, *J* = 8.9, 2.5 Hz, 2H), 7.80 (s, 6H), 6.96 (d, *J* = 11.2 Hz, 6H), 2.52 (d, *J* = 2.1 Hz, 18H), 2.01 (s, 12H), 1.92 (s, 6H). ^13^C NMR (101 MHz, CDCl_3_) δ 177.40, 140.41, 140.23, 139.87, 139.19, 138.79, 135.37, 134.51, 130.51, 130.05, 129.87, 129.82, 126.13, 125.02, 124.79, 123.41, 121.03, 120.94, 120.42, 118.14, 21.16, 20.44, 20.07. HRMS (ESI-MS): [C_68_H_56_N_4_O_2_]^+^ Calcd: 960.44. Found: 860.4384. HPLC analysis, 99.2% pure, retention time 17.1 min in 100% methanol. Calcd for C_68_H_56_N_4_O_2_: C, 84.97%; H, 5.87%, and N, 5.83%. Anal. C, 84.02%, H, 6.08%, and N, 5.70%.

#### 3-(9*H*-Carbazol-9-yl)quinolino[3,2,1-*de*]acridine-5,9-dione (**Cz-DiKTa**)

The procedure is similar to that used for **3Cz-DiKTa** with **BrDiKTa** used in lieu of **Br**_**3**_**DiKTa**. A light orange powder was obtaind after the raw product was purified via column chromatography (EtOAc/hexane = 1:10). Yield: 50% (230 mg). *R*_f_: 0.30 (EtOAc/hexane = 1:10). Mp: 298–299 °C. ^1^H NMR (400 MHz, CDCl_3_) δ 8.86–8.79 (m, 2H), 8.76 (d, *J* = 2.5 Hz, 1H), 8.57 (dd, *J* = 7.9, 1.5 Hz, 1H), 8.42 (d, *J* = 9.0 Hz, 1H), 8.28 (d, *J* = 8.3 Hz, 1H), 8.23–8.19 (m, 2H), 7.95 (dd, *J* = 9.0, 2.6 Hz, 1H), 7.81 (ddd, *J* = 8.7, 7.1, 1.7 Hz, 1H), 7.73 (t, *J* = 7.7 Hz, 1H), 7.60–7.46 (m, 5H), 7.40–7.34 (m, 2H). ^13^C NMR (101 MHz, CDCl_3_) δ 178.54, 178.00, 140.55, 139.71, 139.36, 138.37, 134.92, 133.44, 133.14, 132.99, 131.12, 128.08, 127.71, 126.58, 126.29, 125.59, 125.40, 123.98, 123.72, 123.58, 123.31, 122.16, 120.61, 120.56, 120.08, 109.54. HRMS [M + H]^+^: [C_32_H_19_N_2_O_2_]^+^ Calcd: 463.1438. Found: 463.1361. HPLC analysis, 96.5% pure, retention time 6.8 min in 85% methanol, 15% water. Calcd. for C_32_H_18_N_2_O_2_: C, 83.10%, H, 3.92%, and N, 6.06%. Anal. C, 83.38%, H, 4.02%, and N, 6.07%.

#### 3-(9,9-Dimethylacridin-10(9*H*)-yl)quinolino[3,2,1-*de*]acridine-5,9-dione (**DMAC-DiKTa**)

The procedure is similar to that used for **3Cz-DiKTa** with **BrDiKTa** and DMAC used in lieu of **BrDiKTa** and carbazole. An orange powder was obtained after the raw product was purified via column chromatography (EtOAc/hexane = 1:10). Yield: 80% (400 mg). *R*_f_: 0.20 (EtOAc/hexane = 1:10). Mp: 364–366 °C. ^1^H NMR (400 MHz, CDCl_3_) δ 8.86–8.79 (m, 2H), 8.76 (d, *J* = 2.5 Hz, 1H), 8.57 (dd, *J* = 7.9, 1.5 Hz, 1H), 8.42 (d, *J* = 9.0 Hz, 1H), 8.28 (d, *J* = 8.3 Hz, 1H), 8.23–8.19 (m, 2H), 7.95 (dd, *J* = 9.0, 2.6 Hz, 1H), 7.81 (ddd, *J* = 8.7, 7.1, 1.7 Hz, 1H), 7.73 (t, *J* = 7.7 Hz, 1H), 7.60–7.46 (m, 5H), 7.40–7.34 (m, 2H). 1.75 (s, 6H). ^13^C NMR (101 MHz, CDCl_3_) δ 178.55, 177.90, 140.63, 139.70, 139.41, 139.22, 138.25, 136.12, 133.28, 133.14, 132.99, 130.81, 130.55, 128.06, 126.58, 126.46, 125.61, 125.41, 124.01, 123.58, 123.38, 123.11, 121.16, 120.35, 114.07, 36.09, 31.03. HRMS [M + H]^+^: [C_35_H_24_N_2_O_2_]^+^ Calcd.: 504.1838. Found: 504.1825. HPLC analysis, 98.18% pure, retention time 8.3 min in 85% methanol, 15% water. Calcd. for C_35_H_24_N_2_O_2_: C, 83.31%, H, 4.79%, and N, 5.55%. Anal. C, 83.22%, H, 4.79%, and N, 5.51%.

#### 3-(1,3,6,8-Tetramethyl-9*H*-carbazol-9-yl)quinolino[3,2,1-*de*]acridine-5,9-dione (**TMCz-DiKTa**)

The procedure is similar to that used for **3Cz-DiKTa** with **BrDiKTa** and TMCz used in lieu of **Br**_**3**_**DiKTa** and carbazole. An orange powder was obtaind after the raw product was purified via column chromatography (EtOAc/hexane = 1:10). Yield: 44% (210 mg). *R*_f_: 0.30 (EtOAc/hexane = 1:10). Mp: 328–330 °C. ^1^H NMR (400 MHz, CDCl_3_) δ 8.80 (ddd, *J* = 9.5, 7.7, 1.7 Hz, 2H), 8.64 (d, *J* = 2.5 Hz, 1H), 8.55 (dd, *J* = 7.9, 1.5 Hz, 1H), 8.23 (d, *J* = 8.9 Hz, 1H), 8.17 (d, *J* = 8.4 Hz, 1H), 7.82–7.69 (m, 5H), 7.61–7.53 (m, 1H), 6.99 (d, *J* = 26.6 Hz, 2H), 2.51 (s, 6H), 2.11–1.80 (s, 6H). ^13^C NMR (101 MHz, CDCl_3_) δ 178.49, 178.06, 134.91, 133.32, 133.11, 132.98, 130.43, 129.65, 128.08, 126.63, 126.00, 125.63, 124.63, 124.02, 123.60, 123.42, 121.03, 120.28, 120.24, 118.05, 21.15, 19.99. HRMS [M]^+^: [C_36_H_26_N_2_O_2_]^+^ Calcd: 518.1944. Found: 518.190. HPLC analysis, 97.04% pure, retention time 8.3 min in 85% methanol, 15% water. Calcd. for C_36_H_26_N_2_O_2_: C, 83.37%, H, 5.05%, and N, 5.40%. Anal. C, 83.34%, H, 4.97%, and N, 5.33%.

#### 3-(4-(9*H*-Carbazol-9-yl)phenyl)quinolino[3,2,1-*de*]acridine-5,9-dione (**Cz-Ph-DiKTa**)

To **BrDiKTa** (0.3 g, 0.79 mmol, 1 equiv) under a nitrogen atmosphere were added (4-(9*H*-carbazol-9-yl)phenyl)boronic acid (457 mg, 1.59 mmol, 2 equiv), THF (20 mL), and aqueous NaOH (2 M, 6 mL). Under a positive flow of nitrogen, Pd(PPh_3_)_4_ (46 mg, 0.04 mmol, 0.05 equiv) was added, and the solution was then refluxed for 12 h. The reaction was cooled to room temperature and diluted with DCM (150 mL). The organic layer was washed with water (3 × 50 mL) and then dried with anhydrous sodium sulfate. The solvents were removed under reduced pressure. The crude product was purified by chromatography on silica gel (EtOAc/hexane = 1:15). The corresponding fractions were combined and concentrated under reduced pressure to afford bright yellow solid. Yield: 49% (210 mg). *R*_f_: 0.45 (EtOAc/hexane = 1:5). Mp: 294–295 °C. ^1^H NMR (400 MHz, CDCl_3_) δ 8.83 (ddd, *J* = 9.5, 8.5, 2.0 Hz, 3H), 8.56 (dd, *J* = 7.9, 1.5 Hz, 1H), 8.31 (d, *J* = 8.8 Hz, 1H), 8.25 (d, *J* = 8.4 Hz, 1H), 8.20 (d, *J* = 7.7 Hz, 2H), 8.07 (dd, *J* = 8.8, 2.4 Hz, 1H), 8.04–7.99 (m, 2H), 7.83–7.68 (m, 4H), 7.59–7.51 (m, 3H), 7.51–7.45 (m, 2H), 7.38–7.32 (m, 2H). ^13^C NMR (101 MHz, CDCl_3_) δ 178.63, 174.89, 140.73, 139.77, 139.10, 137.86, 137.68, 137.04, 133.91, 133.15 133.09, 132.87, 131.18, 128.42, 127.97, 127.61, 126.79, 126.53, 126.08, 125.62, 125.40, 123.79, 123.55, 121.15, 120.42, 120.37, 120.17, 117.08, 109.81. HRMS [M + H]^+^: [C_38_H_22_N_2_O_2_] ^+^ Calcd: 538.1681. Found: 538.1672. HPLC analysis: 98.54% pure, retention time 8.3 min in 85% methanol, 15% water. Calcd. for C_38_H_22_N_2_O_2_: C, 84.74%, H, 4.12%, and N, 5.20. Anal. C, 84.29%, H, 4.18%, and N, 5.15%.

### Electrochemistry Measurements

Cyclic voltammetry (CV) analysis was carried out on an Electrochemical Analyzer potentiostat model 620E from CH Instruments at a sweep rate of 100 mV/s. Differential pulse voltammetry (DPV) was conducted with an increment potential of 0.004 V and a pulse amplitude, width, and period of 50 mV, 0.05, and 0.5 s, respectively. Samples were prepared as DCM solutions, which were degassed by sparging with DCM-saturated argon gas for 5 min prior to measurements. All measurements were carried out using 0.1 M DCM solution of tetra-*n*-butylammonium hexafluorophosphate, [*n*Bu_4_N]PF_6_]. An Ag/Ag^+^ electrode was used as the reference electrode, while a platinum electrode and a platinum wire were used as the working electrode and counter electrode, respectively. The redox potentials are reported relative to a saturated calomel electrode (SCE) with a ferrocenium/ferrocene (Fc/Fc^+^) redox couple as the internal standard (0.46 V vs SCE).^24^

### Photophysical Measurements

Optically dilute solutions of concentrations on the order of 10^–5^ or 10^–6^ M were prepared in spectroscopic- or HPLC-grade solvents for absorption and emission analysis. Absorption spectra were recorded at room temperature on a Shimadzu UV-2600 double beam spectrophotometer with a 1 cm quartz cuvette. Molar absorptivity determination was verified by linear regression analysis of values obtained from at least four independent solutions at varying concentrations with absorbance ranging from 0.025 to 0.100.

For emission studies, aerated solutions were bubbled by compressed air for 5 min and the degassed solutions were prepared via three freeze–pump–thaw cycles and spectra were measured using a homemade Schlenk quartz cuvette. Steady-state emission, excitation spectra and time-resolved emission spectra were recorded at 298 K using an Edinburgh Instruments FS5 fluorimeter. Samples were excited at 340 nm for steady-state measurements. Photoluminescence quantum yields for solutions were determined using the optically dilute method,^29^ in which four sample solutions with absorbances of ca. 0.10, 0.075, 0.050, and 0.025 at 360 nm were used. The Beer–Lambert law was found to remain linear at the concentrations of the solutions. For each sample, the linearity between absorption and emission intensity was verified through linear regression analysis, with the Pearson regression factor (*R*^2^) for the linear fit of the data set surpassing 0.9. Individual relative quantum yield values were calculated for each solution, and the values reported represent the slope obtained from the linear fit of these results. The quantum yield of the sample, Φ_PL_, can be determined by the equation ,^30^ where *A* stands for the absorbance at the excitation wavelength (λ_exc_: 360 nm), *I* is the integrated area under the corrected emission curve, and *n* is the refractive index of the solvent with the subscripts “s” and “r” denote sample and reference, respectively. Φ_r_ is the absolute quantum yield of the external reference quinine sulfate (Φ_r_ = 54.6% in 1 N H_2_SO_4_).^31^

An integrating sphere (Hamamatsu, C9920–02) was employed for the photoluminescence quantum yield measurements of thin film samples.^32^ The Φ_PL_ of the films were then measured in air and N_2_ environment by purging the integrating sphere with N_2_ gas flow. Time-resolved PL measurements of the thin films were carried out using the time-correlated single-photon counting and MCS technique. The samples were excited at 375 nm by a pulsed laser diode (PicoQuant, LDH-D-C-375, fwhm < 40 ps, pulse energy = 58.5 ± 1.2 pJ, peak power = 1.5 ± 0.3 W, laser spot diameter = 0.4 ± 0.1 mm, power density = 11.6 ± 3.7 mW/cm^2^) and were kept in a vacuum of <8 × 10^–4^ mbar. The singlet–triplet energy splitting (Δ*E*_ST_) was estimated by recording the prompt fluorescence spectra and phosphorescence emission at 77 K. The films were excited by a femtosecond laser emitting at 343 nm (Orpheus-N, model: SP-06-200-PP). Emission from the samples was focused onto a spectrograph (Chromex imaging, 250is spectrograph) and detected on a sensitive-gated iCCD camera (Stanford Computer Optics, 4Picos) with sub-nanosecond resolution. Phosphorescence spectra were measured from 1 ms after photoexcitation, with an iCCD exposure time was 9 ms. Fluorescence spectra were promptly measured from 1 ns after photoexcitation with an iCCD exposure time was 99 ns.

### Quantum Chemical Calculations

All ground-state optimizations were carried out using the DFT level with Gaussian09^33^ using the PBE0^34^ functional and the 6-31G(d,p) basis set.^35^ Excited-state calculations have been carried out with the Turbomol/6.5 package for SCS-CC2 calculations.^15^ Besides DFT calculations, we have investigated all compounds using spin-component scaled second order coupled-cluster (SCS-CC2). We first optimized the ground state using the SCS-CC2 method considering the cc-pVDZ basis set.^36^ Vertical excited states were carried out on the ground state optimized structure using SCS-CC2 method. Different density plots were used to visualize change in electronic density between the ground and excited state and were visualized using the VESTA package.^37^ Excited-state calculations also have been carried out using time-dependent DFT (TD-DFT) within the Tamm–Dancoff approximation (TDA)^38,39^ with the same functional and basis set as for the ground state geometry optimization.

### OLED Fabrication and Testing

OLED devices were fabricated using precleaned indium–tin oxide (ITO)-coated glass substrates with an ITO thickness of 90 nm. The OLED devices had a pixel size of 2 mm × 1 mm. The small molecules and cathode layers were thermally evaporated using a deposition chamber at 10^–7^ mbar at 0.3 or 0.6 A/s for organic layers and 3 A/s for cathode. OLED testing was carried out using a Keithley 2400 sourcemeter and photodiode, assuming that the OLEDs show Lambertian emission. Electroluminescence spectra were collected using an Oriel MS125 spectrograph coupled to an Andor DV420-BU CCD camera.

### Calculation of Out-Coupling Efficiency of OLEDs

Dipole orientation of emitter molecules was determined by angle-resolved PL measurements of thin films doped with each emitter. Our out-coupling simulation of the OLEDs is based on emission dipole as forced damped harmonic oscillator and embedded in thin film stacks.

## Supporting Information Available

Cartesian coordinates (XYZ)
^1^H and ^13^C NMR spectra, HRMS and HPLC of all target compounds; supplementary computational data; supplementary photophysical data(PDF)

## Supplementary Material

am2c02756_si_001.xyz

## Notes

The research data supporting this publication can be accessed at https://doi.org/10.17630/8ba5fd13-8d45-4967-95c7-1fceb947612b.
