# Supplementary material for: Excited-State Modulation in Donor-Substituted Multiresonant Thermally Activated Delayed Fluorescence Emitters
Source: ACS Appl Mater Interfaces. 2022 May 9;14(19):22341–52. doi: 10.1021/acsami.2c02756 (PMC9121343; doi:10.1021/acsami.2c02756)
Supplement: Supplementary file 2 — am2c02756_si_002.pdf [file am2c02756_si_002.pdf]

Supporting Information

Excited state modulation in Donor-Substituted Multi-resonant Thermally Activated Delayed Fluorescence Emitters

Sen Wu,[a] Wenbo Li,[b] Kou Yoshida,[b] David Hall,[a,c] Subeesh Madayanad Suresh,[a] Thomas Sayner,[b] Junyi Gong,[b] David Beljonne,[c] Yoann Olivier,[d] Ifor D. W. Samuel[b*] and Eli Zysman-Colman[a*]*

[a]Organic Semiconductor Centre, EaStCHEM School of Chemistry, University of St Andrews, St Andrews, Fife, UK, KY16 9ST, Fax: +44-1334 463808; Tel: +44-1334 463826; E-mail: eli.zysman-colman@st-andrews.ac.uk.

[b]Organic Semiconductor Centre, SUPA School of Physics and Astronomy, University of St Andrews, St Andrews, UK, KY16 9SS; E-mail: idws@st-andrews.ac.uk

[c]Laboratory for Chemistry of Novel Materials, University of Mons, 7000, Mons, Belgium.

[d]Laboratory for Computational Modeling of Functional Materials & Solid State Physics Laboratory, Namur Institute of Structured Matter, University of Namur, Rue de Bruxelles, 61, 5000 Namur, Belgium. E-mail: yoann.olivier@unamur.be

Table of Contents

General methods **S3**

Literature Study **S9**

Theoretical calculation **S10**

Optoelectronic characterization **S15**

Device section S**22**

Reference **S49**

General methods

General Synthetic Procedures. All reagents and solvents were obtained from commercial sources and used as received unless otherwise stated. Air-sensitive reactions were done under a nitrogen atmosphere using Schlenk techniques. Dry solvents used in the reaction were obtained from a MBRAUN SPS5 solvent purification system. Flash column chromatography was carried out using silica gel (Silia-P from Silicycle, 60 Å, 40-63 μm). Analytical thin-layer-chromatography (TLC) was performed with silica plates with aluminium backings (250 μm with F-254 indicator). TLC visualization was accomplished by 254/365 nm UV lamp. HPLC analysis was conducted on a Shimadzu LC-40 HPLC system. HPLC traces were performed using a Shim-pack GIST 3μm C18 reverse phase analytical column. ^{1}H and =C and NMR spectra were recorded on a Bruker Advance spectrometer (500 MHz for ^{1}H and 125 MHz for ^{13}C). The following abbreviations have been used for multiplicity assignments: "s" for singlet, "d" for doublet, "t" for triplet, "q" for quartet, "m" for multiplet. ^{1}H and ^{13}C NMR spectra were referenced to the solvent peaks). Melting points were measured using open-ended capillaries on an Electrothermal 1101D Mel-Temp apparatus and are uncorrected. High-resolution mass spectrometry (HRMS) was performed at the BBSRC Mass Spectrometry Facility, University of St Andrews. Elemental analyses were performed by Joe Casillo at the University of Edinburgh.

Fitting of time-resolved luminescence measurements: Time-resolved PL measurements were fitted to a sum of exponentials decay model, with chi-squared (χ^2) values between 1 and 2, using the EI FLS980 software. Each component of the decay is assigned with a weight, (w_i), which is the contribution of the emission from each component to the total emission.

The average lifetime was then calculated using the following expressions:[1]

1. Two exponential decay model:

$$\tau_{AVG} = \tau_1 w_1 + \tau_2 w_2 \qquad \text{(S1)}$$

with weights defined as $w_1 = \frac{A1\tau_1}{A1\tau_1 + A2\tau_2}$ and $w_2 = \frac{A2\tau_2}{A1\tau_1 + A2\tau_2}$ where A_1 and A_2 are the preexponential-factors of each component.

2. Three exponential decay model:

$$\tau_{AVG} = \tau_1 w_1 + \tau_2 w_2 + \tau_3 w_3 \qquad \text{(S2)}$$

with weights defined as $w_1 = \frac{A1\tau_1}{A1\tau_1 + A2\tau_2 + A3\tau_3}$, $w_2 = \frac{A2\tau_2}{A1\tau_1 + A2\tau_2 + A3\tau_3}$ and $w_3 = \frac{A3\tau_3}{A1\tau_1 + A2\tau_2 + A3\tau_3}$ where A_1, A_2 and A_3 are the preexponential-factors of each component.

Determination of emitter dipole orientation by angle-resolved PL measurement:

Dipole orientation of emitter molecules was determined by angle-resolved PL measurements of thin films doped with each emitter. The experiment used p-polarized light and the results were fitted to an optical simulation of the film stack.[1, 2] The measured PL intensity of p-polarized light has contributions from vertical dipoles and horizontal dipoles, with the relative amount depending on the dipole orientation.[2] To quantify this, an anisotropy factor (a) was used, which is defined by the ratio of emitted power by vertical dipoles to total emitted power by all dipoles.[3] We note that for perfectly horizontal dipole orientation (i.e. parallel to the substrate surface), a = 0, for isotropic orientation, a = 1/3, and for perfectly vertical orientation, a = 1. The calculated results for each dipole were combined to fit the experimental results with the anisotropy factor, a. The experimental results on PL intensity of p-polarized light ($I_{p,exp}$) can be expressed as follows: $I_{p,exp}$ = a $I_{p,vert}$ + (1-a)/2 $I_{p,hor}$, where $I_{p,ver}$ is calculated intensity from vertical dipole emitting p-polarized lights and $I_{p,ver}$ is calculated intensity from horizontal dipole emitting p-polarized lights.[3] We note that the factor 2 is to consider the contribution to the total emission from horizontal dipoles emitting s-polarized light.

s-polarized light is used to check the optical model or to get actual film thickness by comparing the calculated results and experimental results.

Figure **S1** shows a schematic of our angle-resolved PL measurement system. The film samples were prepared by thermal evaporation on fused silica substrates (UQG Ltd.). The nominal thicknesses of the films were 50 nm, which was determined by a calibrated quartz crystal microbalance thickness monitor. The film samples were encapsulated in a globe box with a glass lid (Shanghai Mega-9 Optoelectronic Co., Ltd.) and a UV curable epoxy glue (Norland Products Inc., Norland Optical Adhesive 68). This is to avoid photo-oxidation of the films during the measurements. A custom-made semi-cylindrical lens made of fused silica (Nanyang Jingying Trade Co., Ltd) was attached to the film samples from the substrate side. A refractive index matching fluid (McCrone UL Limited, Refractive Index Liquid Series A with a refractive index of 1.46) was used between the substrates to reduce complication in the optical simulation caused by air interfaces. The films were excited by a CW He-Cd Laser (Kimmon Koha) at an excitation wavelength of 325 nm through the glass lid. A quarter wave plate was used to excite the films by circularly polarised light. The angle-resolved emission from films was recorded with a fibre-coupled CCD spectrometer. The spectrometer consisted of a CCD camera (Andor Technology Ltd., DV420-BV) and a spectrograph (Oriel, 77400 MS125™). The head of the fibre was attached to a computer-controlled rotation stage (ESP) and used to detect emission from the films at different angles. The angle was scanned from 0° to 180° to check symmetry of the measurement results and to make sure the good optical alignment of the set-up. To selectively detect each polarized component, a polarizer (Thorlabs, LPVISE100-A) was placed between the fibre head and the lens. Also, a 400-nm-long pass filter (Thorlabs, FEL0400) was placed to reduce the signal from the excitation to the CCD. To monitor photo-degradation of the films

and fluctuation of excitation intensity, a photodiode (Thorlabs, PDA100A2) was placed above a beam path of excitation light (see Figure **S1**). The signal from the photodiode was measured by a multi-meter (Keithley, 2000). The intensity measured by the CCD at each angle was divided by the detected intensity by the photodiode at the same time. The divided intensity was used to compare with the optical simulation. To reduce the photo-degradation of the samples, a custom-made mechanical shutter was used to minimize exposure of the excitation laser to the films. Also, integration time and data points, i.e., angle steps, were optimized to minimize any degradation of the sample. All measurements were fully automated using a custom-made program written in Python.

The optical simulation considers the emission dipole as a forced damped harmonic oscillator and embedded in the thin film stack. Details are described in the references.[4-6] The simulation was conducted by a custom-made Python program. For the simulation, optical constants of host matrixes were measured by a variable angle spectroscopic ellipsometer (J. A. Woollam, M-2000 Ellipsometer). They were used to simulate films doped with different emitters assuming that the optical constants of the film doped with different emitters at low concentrations (<10 wt%) are the same as that of the host. The refractive index of other components such as the matching fluid, and the lens were provided by the suppliers and close to 1.46. The position of the dipole along the film thickness direction is important for calculating the angular dependent PL intensity. The calculated results at different positions were combined with weighting by the absorbed light at the position. The absorbed light was calculated by the Beer-Lambert law and absorption coefficient obtained from imaginary part of refractive index of host matrixes at the excitation wavelength, i.e., 325 nm. The calculation was conducted at peak PL emission wavelength of the films while the measured PL intensity was taken by integrating the measured intensity around the peak of the emission spectrum.

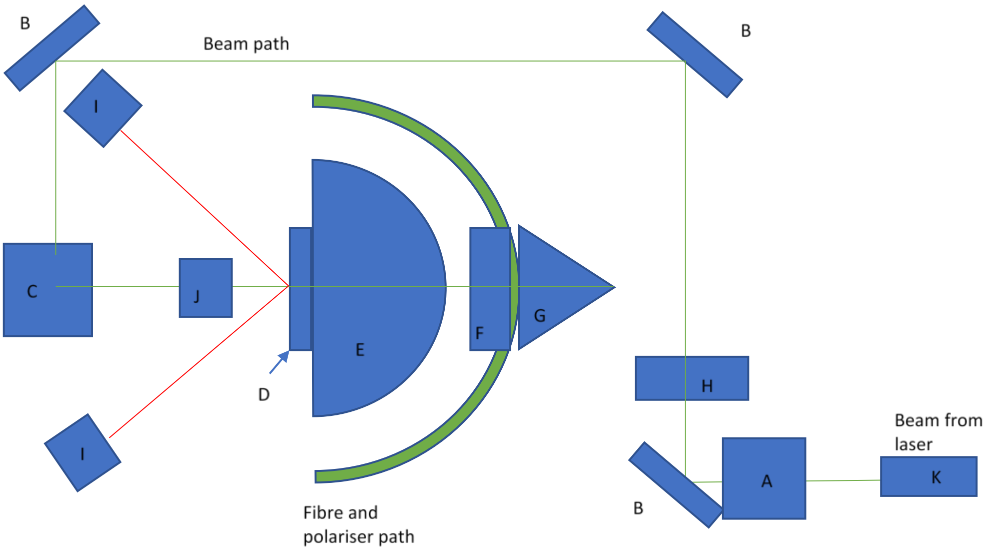

Figure **S1**. Schematic of our angle-resolved PL measurement system. In the figure, each component is as follows: A-shutter, B-mirrors, C-periscope, D-sample (attached to lens), E-semi cylindrical lens mounted on translation stage, F-polariser and long pass filter, G- optical fibre to CCD (mounted on rotation stage), H-quarter wave plate, I-alignment lasers, J-photodiode to connected to multimeter (mounted above beam path), K: attenuator and the CW He-Cd Laser.

Calculation of out-coupling efficiency of OLEDs: Our out-coupling simulation of the OLEDs is based on emission dipole as forced damped harmonic oscillator and embedded in thin film stacks. Details are described in the references.[4-6] Based on the mathematical details by Furno and co-workers,[5] out-coupling efficiency was calculated by the same program for the angle-resolved PL. For the simulation, optical constants of organic materials, which were thermally evaporated on glass, were measured by the same ellipsometer for the angle-resolved PL measurements and used. The PL spectra of thermally evaporated films, which are same as the emission layers of the OLEDs, were used for the simulation. The anisotropic factors of the emitter molecules measured by the angular resolved PL were considered in the simulation. The out-coupling was calculated within a wavelength range from 400 nm to 700 nm with a 1 nm step. At each

wavelength, dissipation powers were calculated for normalized in-plane wave vector from 0 to 2 with a step size of 0.002.

Angular dependent EL intensity of the OLEDs was also calculated by the same optical simulation. It was used to estimate the ratio of EQE estimated by the assumption Lambertian to EQE estimated by the calculated angular distribution (Discrepancy from Lambertian).

In the optical calculation, it was assumed that emitter dipole is localized at mCP and PPT interface. This is reasonable because the hole conduction is dominate in the host, mCP. The effect of dipole position within the emission layer on out-coupling efficiency and the discrepancy from the Lambertian were also calculated. The difference in the calculated results is lower than 10% in ratio (See Figure **S2**)

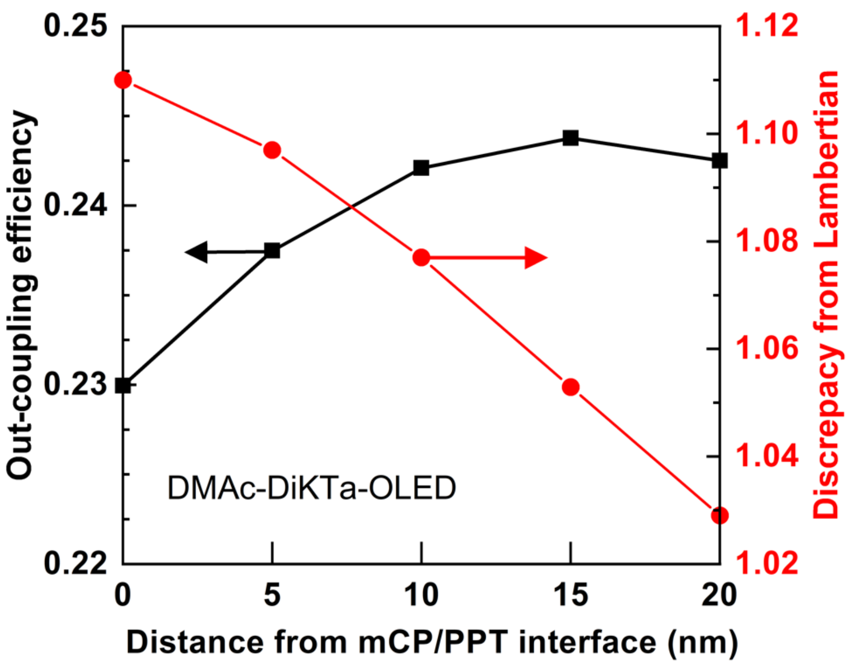

Figure **S2**. Effect of dipole position on out-coupling and discrepancy from Lambertian of OLEDs based on DMAc-DiKTa. Dipole position was changed within the emission layer.

Literature Study

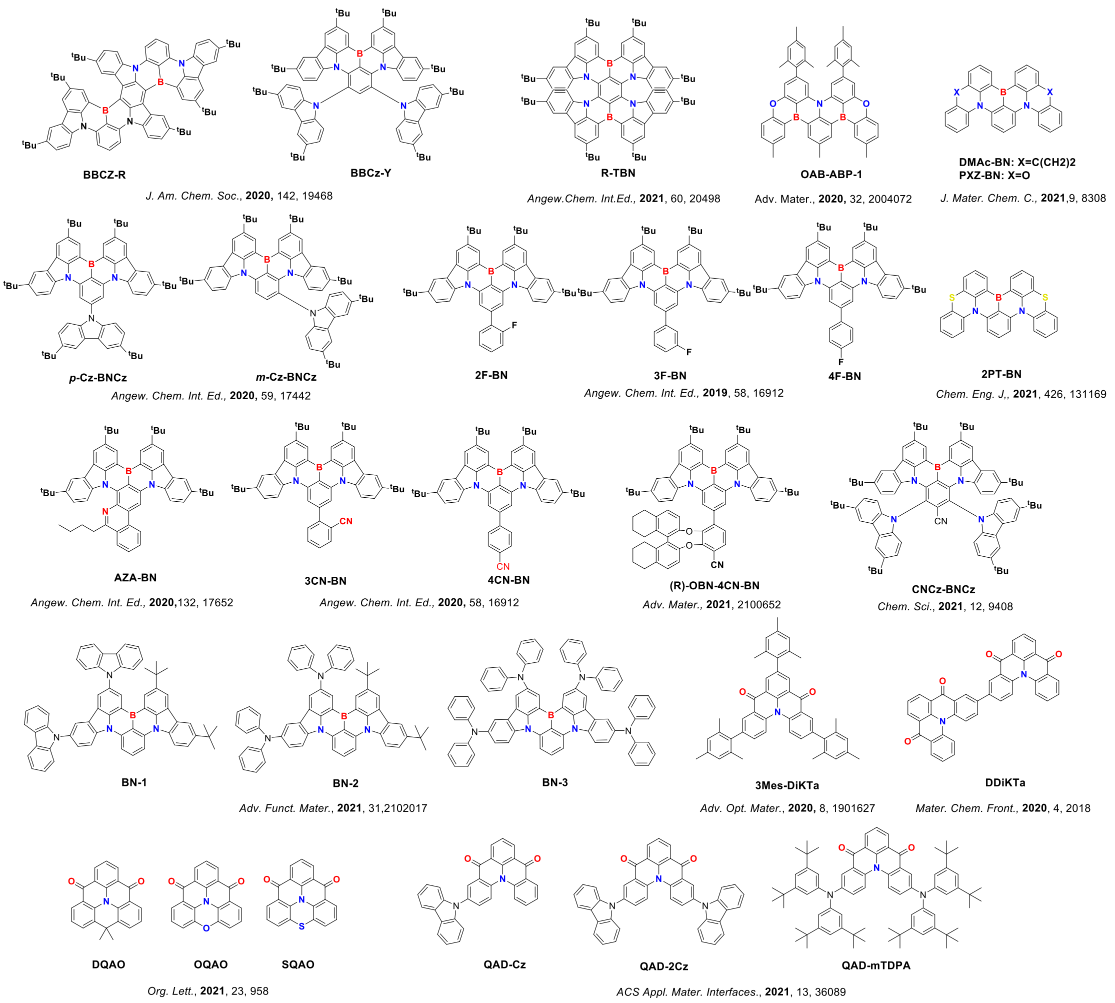

Figure **S3.** Molecular structures of MR-TADF emitters discussed in the main text.

Calculations

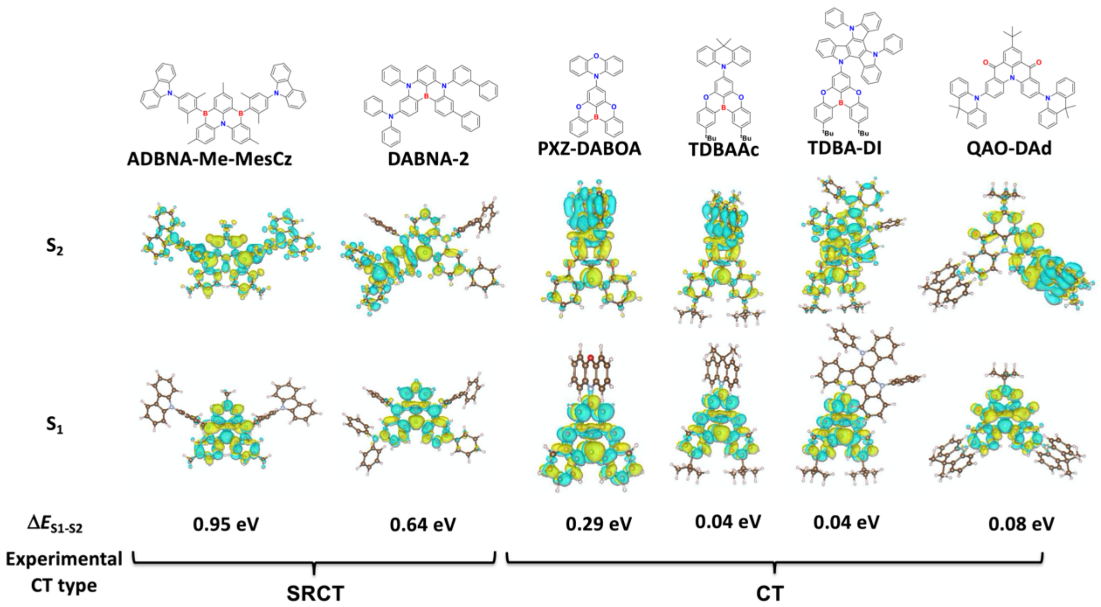

Figure **S4.** Difference density plots for literature compounds using SCS-CC2 method with cc-pVDZ basis set calculated in the gas phase. Blue balls represent a decrease in density and green balls represent increase in density.

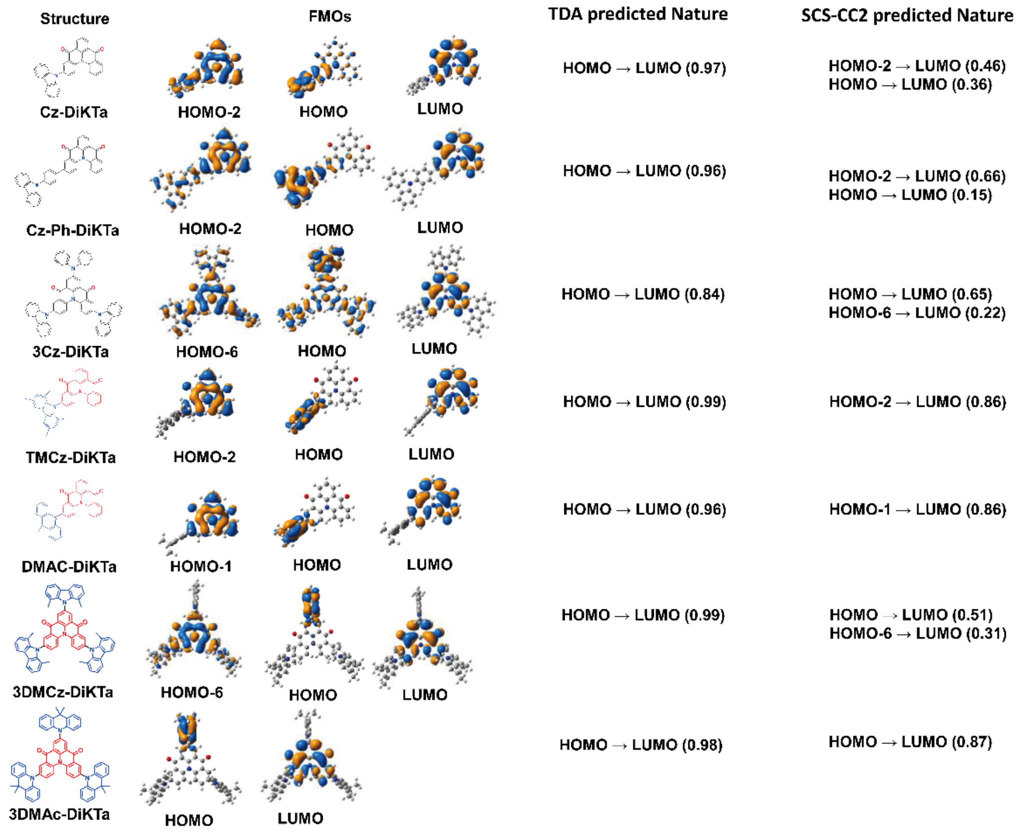

Figure **S5.** FMOs orbitals calculated in the gas phase at the PBE0/6- 31G(d,p) level and the electronic transition nature calculated using TDA-DFT and SCS-CC2 in gas phase.

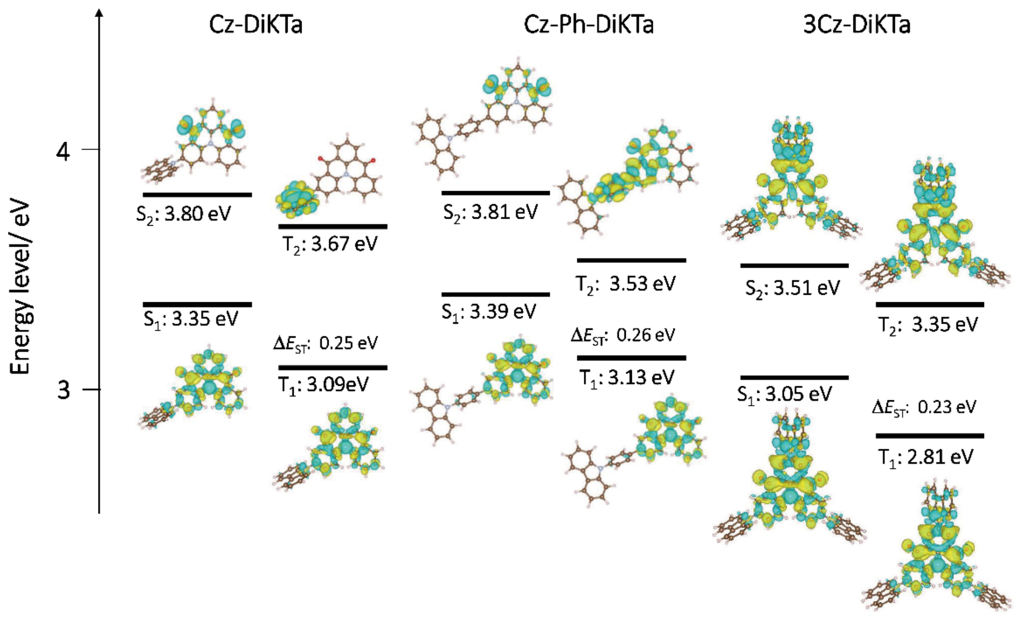

Figure **S6.** Difference density plots of S_1, S_2, T_1 and T_2 excited states calculated in the gas phase at the SCS-CC2/cc-pVDZ level for **Cz-DiKTa**, **Cz-Ph-DiKTa** and **3Cz-DiKTa**.

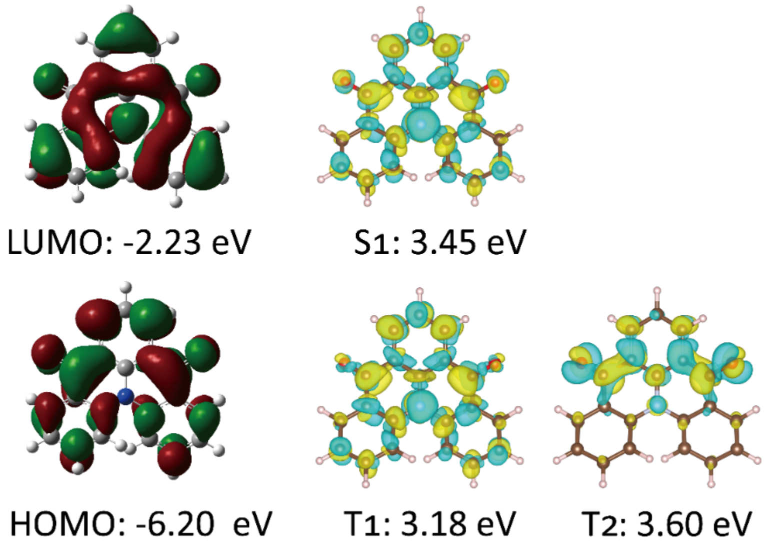

Figure **S7.** HOMO and LUMO orbitals calculated in the gas phase at the PBE0/6-31G(d,p) level and difference density plots of S1, T1 and T2 excited states calculated in the gas phase at the SCS-CC2/cc-pVDZ level.

Table **S1.** The summary of the calculated data calculated in the gas phase at the PBE0/6-31G(d,p) level.

Compound	FMOs / eV	S_1 / eV	T_1 / eV	ΔE_{ST} / eV	*f*
**Cz-DiKTa**	H: -5.73; H-2: -6.42; L: -2.36	2.83	2.49	0.34	0.05
**Cz-Ph-DiKTa**	H: -5.65; H-2: -6.21; L: -2.31	2.97	2.58	0.39	0.07
**3Cz-DiKTa**	H: -5.67; H-6: -6.76; L: -2.63	2.48	2.14	0.34	0.06
**TMCz-DiKTa**	H: -5.44; H-2: -6.37; L: -2.41	2.50	2.49	0.01	0.00
**DMAc-DiKTa**	H: -5.26; H-1: -6.33; L: -2.37	2.34	2.32	0.02	0.00
**3DMCz-DiKTa**	H: -5.66; H-6: -6.61; L: -2.80	2.08	2.01	0.07	0.00
**3DMAc-DiKTa**	H: -5.26; L: -2.62	1.94	1.91	0.03	0.00

Where H6=HOMO-6, H2=HOMO-2, H1=HOMO-1, H=HOMO, L=LUOMO.

Table **S2.** The summary of the calculated data calculated in the gas phase at the SCS-CC2/cc-pVDZ level.

Compound	S_1 / eV (D_{CT}/Å)	Nature	S_2 / eV (D_{CT} /Å)	T_1 / eV (D_{CT} /Å)	T_2 / eV (D_{CT}/Å)	ΔE_{ST} / eV	*f*
**Cz-DiKTa**	3.35 (1.36)	H2L (0.46) HL (0.36)	3.80 (0.14)	3.09 (1.02)	3.67 (0.25)	0.26	0.214
**Cz-Ph-DiKTa**	3.39 (1.05)	H2L (0.66) HL (0.15)	3.81 (0.14)	3.13 (0.81)	3.53 (0.30)	0.26	0.254
**3Cz-DiKTa**	3.05 (0.45)	HL (0.65) H6L (0.22)	3.51 (1.15)	2.81 (0.33)	3.35 (1.39)	0.24	0.191
**TMCz-DiKTa**	3.45 (0.85)	H2L (0.86)	3.79 (0.26)	3.18 (0.64)	3.58 (0.61)	0.27	0.227
**DMAc-DiKTa**	3.43 (0.82)	H1L (0.86)	3.51 (3.18)	3.17 (0.61)	3.46 (2.93)	0.26	0.229
**3DMCz-DiKTa**	3.19 (2.22)	HL (0.51) H6L (0.31)	3.44 (1.48)	3.01 (0.86)	3.31 (2.44)	0.18	0.100
**3DMAc-DiKTa**	2.97 (1.67)	HL (0.87)	3.31 (3.49)	2.94 (3.31)	3.13 (0.42)	0.03	0.000

Where H6=HOMO-6, H2=HOMO-2, H1=HOMO-1, H=HOMO, L=LUOMO. *f* is the oscillator strength.

Synthesis

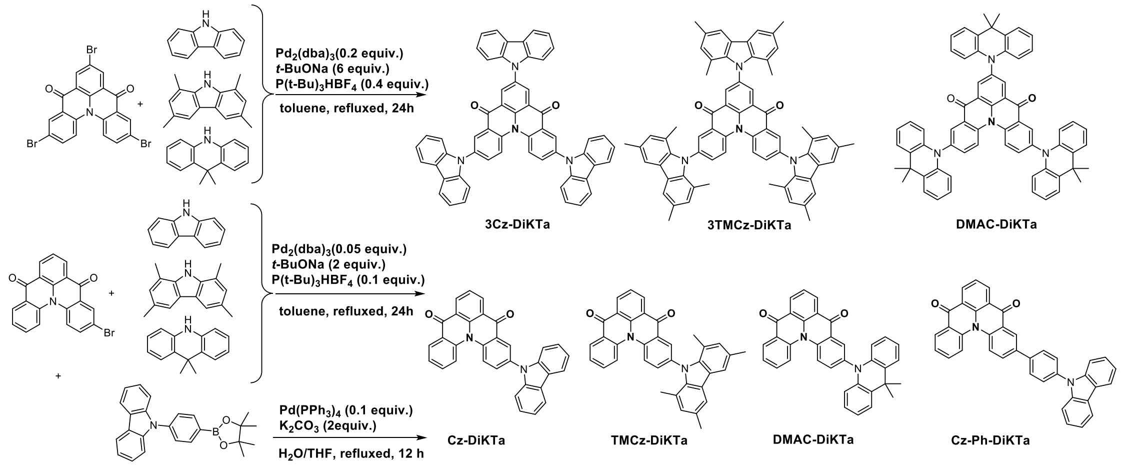

Scheme **S1**. Synthetic route for **Cz-DiKTa, Cz-Ph-DiKTa, TMCz-DiKTa, DMAC-DiKTa, 3Cz-DiKTa, 3TMCz-DiKTa** and **3DMAc-DiKTa**.

Br$_3$DiKTa and **BrDiKTa** were synthesised following a previously reported protocol.[7, 8]

Optoelectronic characterization

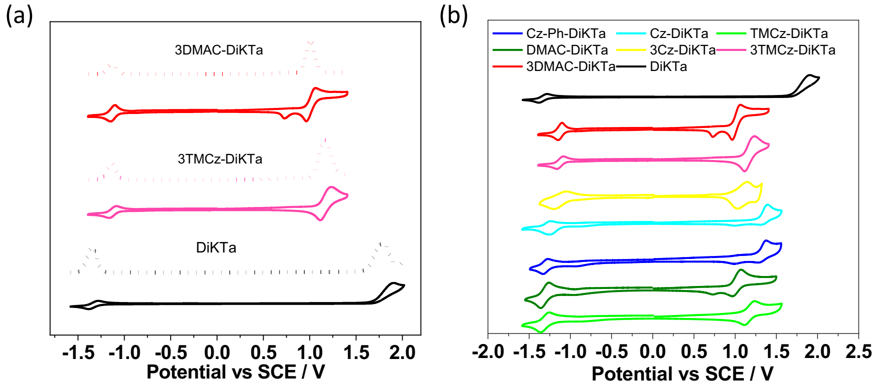

Figure **S8**. Cyclic voltammogram (CV) and differential pulse voltammetry (DPV) for (a) **3DMAC-DiKTa**, **3TMCz-DiKTa** and **DiKTa**. (b) CVs for **Cz-DiKTa**, **Cz-Ph-DiKTa**, **TMCz-DiKTa**, **DMAC-DiKTa**, **3Cz-DiKTa**, **3TMCz-DiKTa**, **3DMAC-DiKTa** and **DiKTa**. in degassed DCM with 0.1 M [*n*Bu_4N]PF_6 as the supporting electrolyte and Fc/Fc^+ as the internal reference (0.46 V vs. SCE).[9]

Table **S3.** Electrochemical data.

	DiKTa	3Cz-DiKTa	3TMCz-DiKTa	3DMAC-DiKTa	Cz-DiKTa	Cz-Ph-DiKTa	TMCz-DiKTa	DMAC-DiKTa
**E_{ox} / V**	1.78	1.02	1.17	0.97	1.35	1.27	1.14	1.02
**E_{red} / V**	-1.34	-1.15	-1.14	-1.14	-1.3	-1.33	-1.34	-1.31
**HOMO / eV**	-6.12	-5.36	-5.51	-5.31	-5.69	-5.61	-5.48	-5.36
**LUMO / eV**	-3. 00	-3.19	-3.20	-3.20	-3.04	-3.01	-3.00	-3.03

In degassed DCM with 0.1 M [*n*Bu_4N]PF_6 as the supporting electrolyte and Fc/Fc^+ as the internal reference (0.46 V vs. SCE).[9] The HOMO and LUMO energies were determined using the relation $E_{HOMO/LUMO} = -(E_{ox} / E_{red} + 4.8)$ eV,[10] where E_{ox} and E_{red} are the peak of anodic and cathodic potentials from DPV relative to Fc/Fc^+.

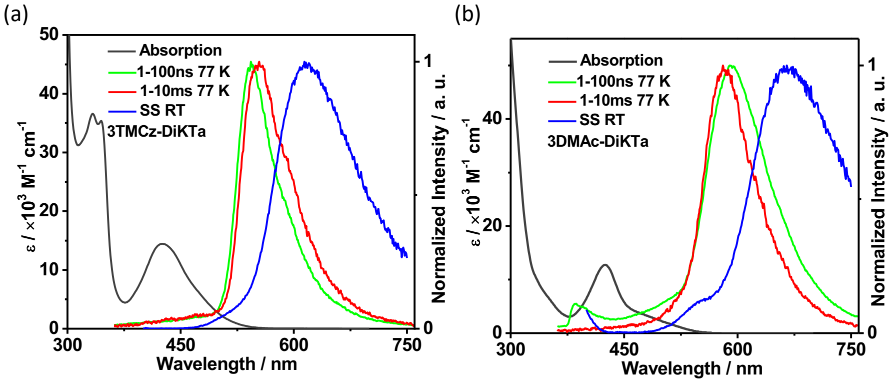

Figure **S9.** Absorption and steady-state PL spectra obtained in toluene at RT, prompt PL (1-100 ns) and phosphorescence spectra (1-10 ms) obtained in toluene glass at 77 K, measured by iCCD (λ_{exc} = 340 nm) for (a) **3TCz-DiKTa** and (b) **3DMAC-DiKTa.**

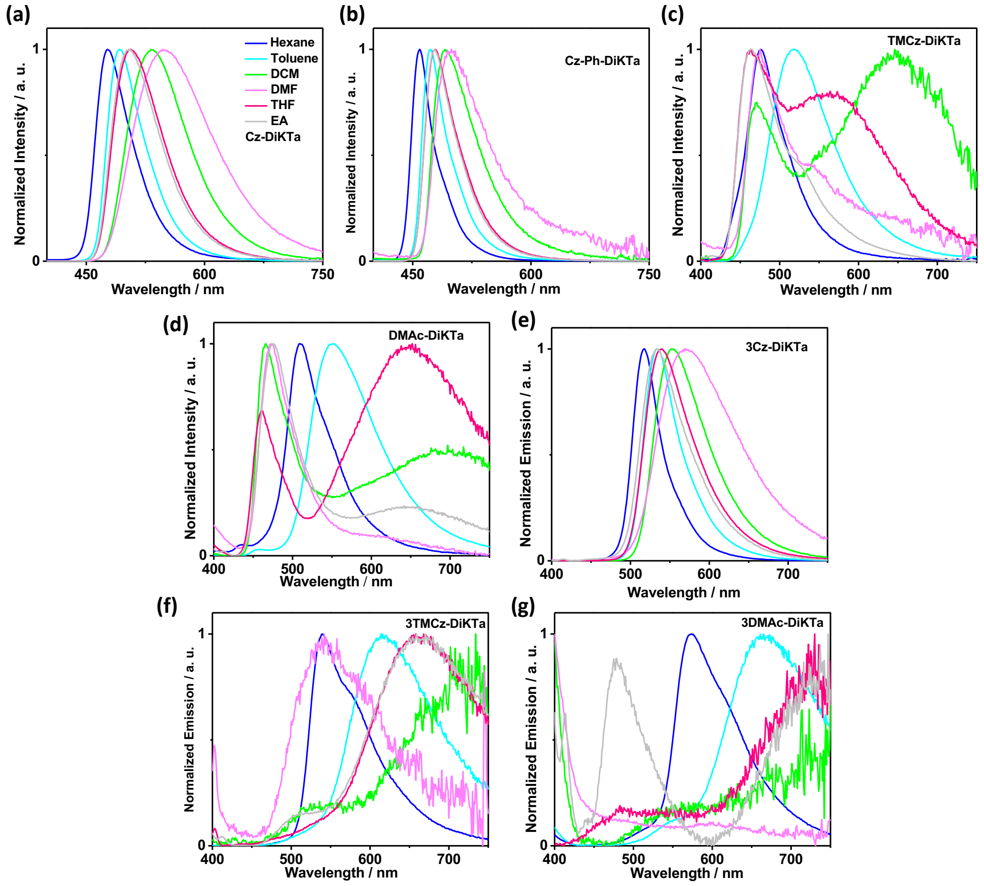

Figure **S10**. Solvatochromatic PL study, (a) **Cz-DiKTa** (b) **Cz-Ph-DiKTa** (c) **TMCz-DiKTa** (d) **DMAC-DiKTa** (e) **3Cz-DiKTa** (f) **3TMCz-DiKTa** and (g) **3DMAC-DiKTa** (λ_{exc} = 340 nm).

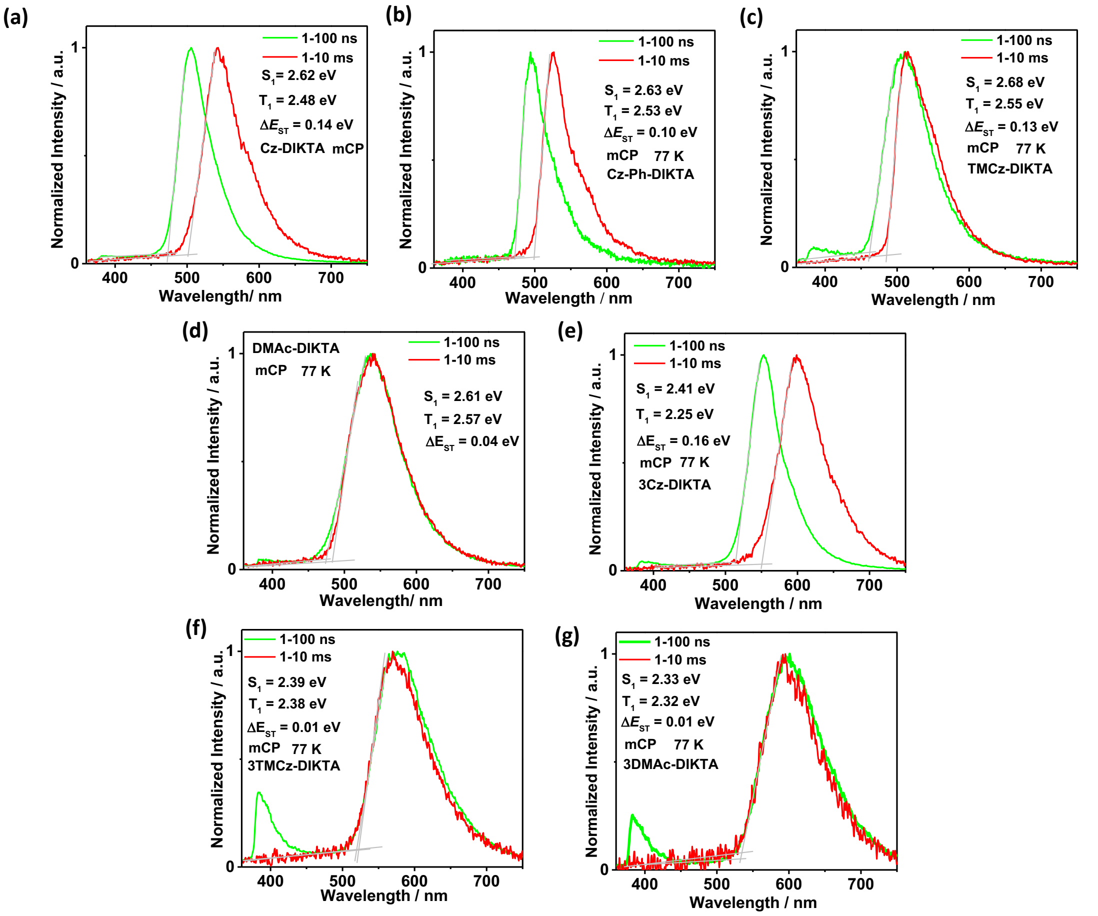

Figure **S11**. Prompt PL and phosphorescence spectra in 2 wt% mCP at 77 K, (a) **Cz-DiKTa** (b) **Cz-Ph-DiKTa** (c) **TMCz-DiKTa** (d) **DMAC-DiKTa** (e) **3Cz-DiKTa** (f) **3TMCz-DiKTa and** (g) **3DMAC-DiKTa**, λ_{exc} = 340 nm. The feature at 380 nm in the prompt spectra is from the mCP host.

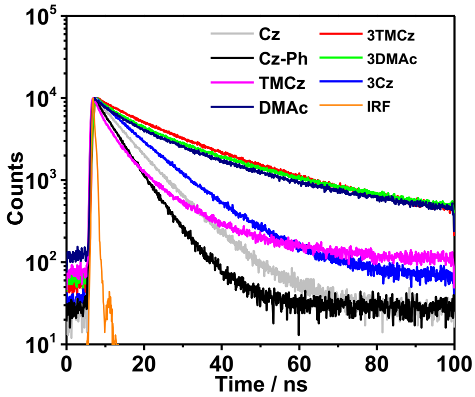

Figure **S12**. Time-resolved PL decay profiles of **Cz-DiKTa, Cz-Ph-DiKTa, TMCz-DiKTa, DMAC-DiKTa, 3Cz-DiKTa, 3TMCz-DiKTa and 3DMAc-DiKTa** in 2 wt% mCP within 100 ns time window.

Table **S4.** Solution-state photophysical data in toluene (10^{-5} M)

**Compound**	λ_{Abs}[a] / nm	λ_{Pl}[b] / nm	**FWHM**[b] / nm	Φ_{PL}[c] / %	T_1[d] / eV	S_1[e] / eV	ΔE_{ST}[f] / eV
**Cz-DiKTa**	313, 339, 376, 443	495	54	50	2.46	2.66	0.20
**Cz-Ph-DiKTa**	325, 343, 420, 443	472	47	34	2.5	2.72	0.22
**TMCz-DiKTa**	325, 336, 414, 434	516	80	12	2.55	2.76	0.21
**DMAC-DiKTa**	343, 431	551	94	16	2.56	2.67	0.11
**3Cz-DiKTa**	327, 338, 365,491	534	53	46	2.25	2.48	0.23
**3TMCz-DiKTa**	332, 346, 426	617	110	-	2.44	2.41	0.03
**3DMAc-DiKTa**	335, 425	667	116	-	2.33	2.34	0.01

[a]Obtained under aerated conditions at 298 K. [b] Concentration 1.2×10^{-5} M, λ_{exc} = 345 nm, [c] Obtained in toluene solution, λ_{exc} = 327-343 nm in toluene; [d]Obtained from the onset of the delayed spectrum at 77 K 1-100 ns, λ_{exc} = 355 nm. [e]Obtained from the onset of the prompt spectrum at 77 K, λ_{exc} = 355 nm, 1-10 ms. [f]$\Delta E_{ST} = E(S_1) - E(T_1)$.

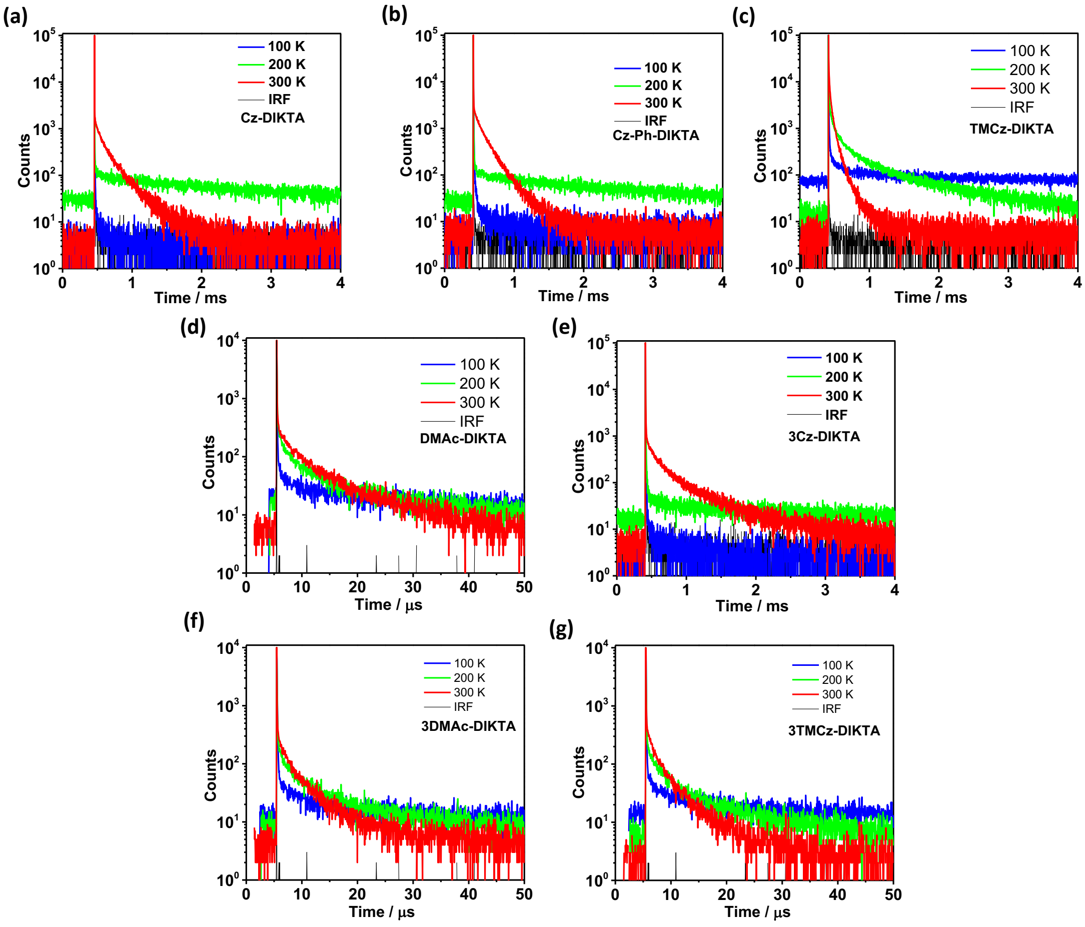

Figure **S13**. Temperature-dependent time-resolved PL decay, λ_{exc} = 378 nm in 2 wt% mCP, a) **Cz-DiKTa** (b) **Cz-Ph-DiKTa** (c) **TMCz-DiKTa** (d) **DMAC-DiKTa** (e) **3Cz-DiKTa** (f) **3TMCz-DiKTa** and (g) **3DMAC-DiKTa**.

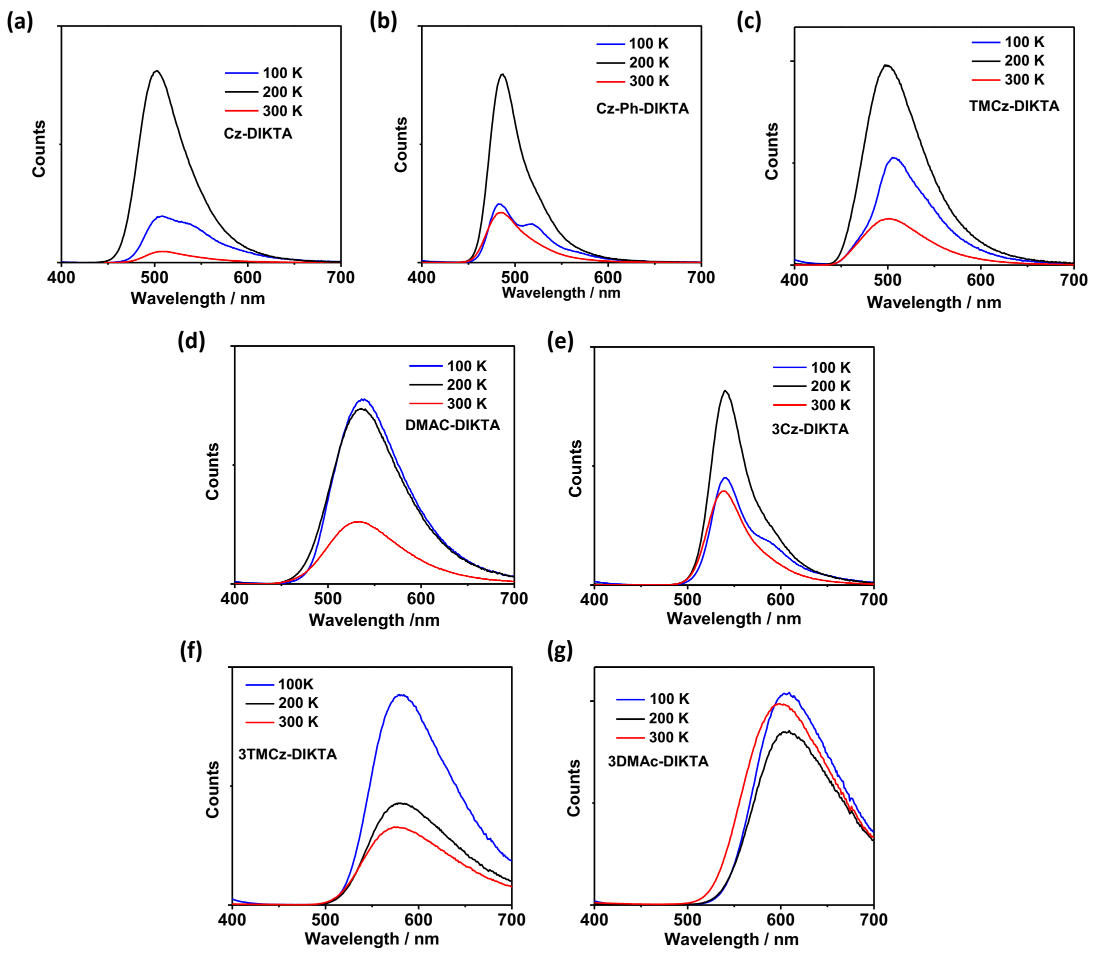

Figure **S14.** Temperature-dependent steady-state PL spectra in 2 wt% mCP, λ_{exc} = 340 nm, (a) **Cz-DiKTa,** (b) **Cz-Ph-DiKTa** (c) **TMCz-DiKTa** (d) **DMAC-DiKTa** (e) **3Cz-DiKTa** (f) **3TMCz-DiKTa** and (g) **3DMAC-DiKTa.**

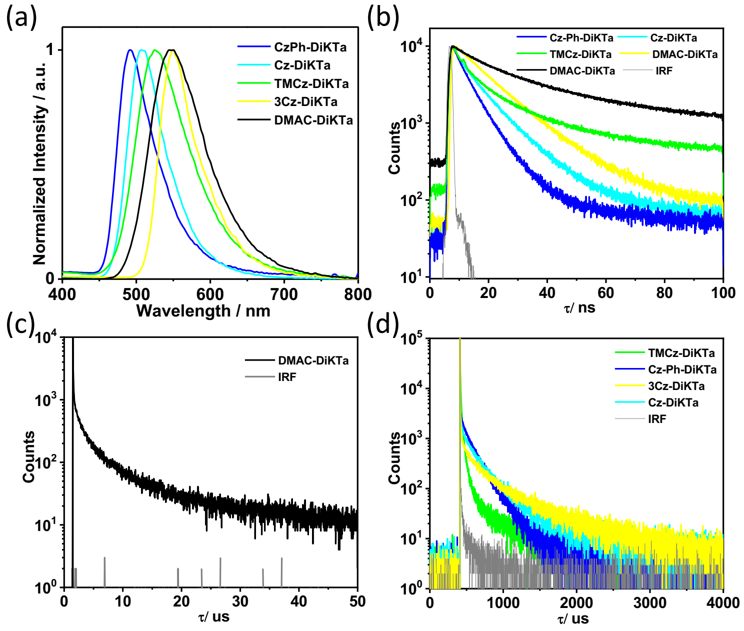

Figure **S15.** (a) PL spectra of **Cz-DiKTa, Cz-Ph-DiKTa, 3Cz-DiKTa, TMCz-DiKTa, DMAC-DiKTa** in 7.5 wt% doped mCP films at room temperature, λ_{exc} = 340 nm. (b) Time-resolved PL decay profiles of **Cz-DiKTa, Cz-Ph-DiKTa, TMCz-DiKTa, DMAC-DiKTa** and **3Cz-DiKTa** in 7.5 wt% mCP within 100 ns time window; (c) Time-resolved PL decays of **DMAC-DiKTa** in 7.5 wt% doped mCP films. λ_{exc} = 379 nm. (d) Time-resolved PL decays of **Cz-DiKTa, Cz-Ph-DiKTa, 3Cz-DiKTa** and **TMCz-DiKTa** in 7.5 wt% mCP, λ_{exc} = 379 nm.

Table **S5.** Quantum yield screening in different host matrices.

Emitter	Concentration	Host	Φ_{PL} (N_2)
**3Cz-DiKTa**	2%	PPT	62%
		TPBi	58%
		mCP	78%
**Cz-DiKTa**	2%	PPT	68%
		TPBi	72%
		mCP	90%

Calculated using an integrating sphere, under N_2 at λ_{exc} = 340 nm.

Table **S6.** Quantum yield screening at different doping concentrations.

Emitter	Concentration	Φ_{PL} (N_2) /%	Emitter	Concentration	Φ_{PL} (N_2) /%
**3Cz-DiKTa**	0.5 wt%	65	**Cz-Ph-DiKTa**	0.5 wt%	73
	1 wt%	71		2 wt%	77
	2 wt%	78		5 wt%	68
**Cz-DiKTa**	0.5 wt%	76		10 wt%	51
	1 wt%	78	**DMAC-DiKTa**	1 wt%	65
	2 wt%	90		2 wt%	76
	5 wt%	70	**TMCz-DiKTa**	1 wt%	63
	10 wt%	59		2 wt%	71

Calculated using an integrating sphere, under N_2 at λ_{exc} = 340 nm.

Table **S7**. Quantum yield of emitters in 7.5 wt% mCP doped film fabricated by different methods.[a]

Emitters	Vacuum-deposited 7.5 wt%		Solution-processed 2 wt%	
	Φ_{PL} / %	λ_{max}/nm	Φ_{PL} / %	λ_{max}/nm
**Cz-DiKTa**	77	507	90	502
**Cz-Ph-DiKTa**	69	492	77	486
**3Cz-DiKTa**	74	549	78	539
**TMCz-DiKTa**	57	525	71	501
**DMAC-DiKTa**	62	546	76	534

[a] Measured using an integrating sphere, under N_2 at λ_{exc} = 340 nm.

Device section

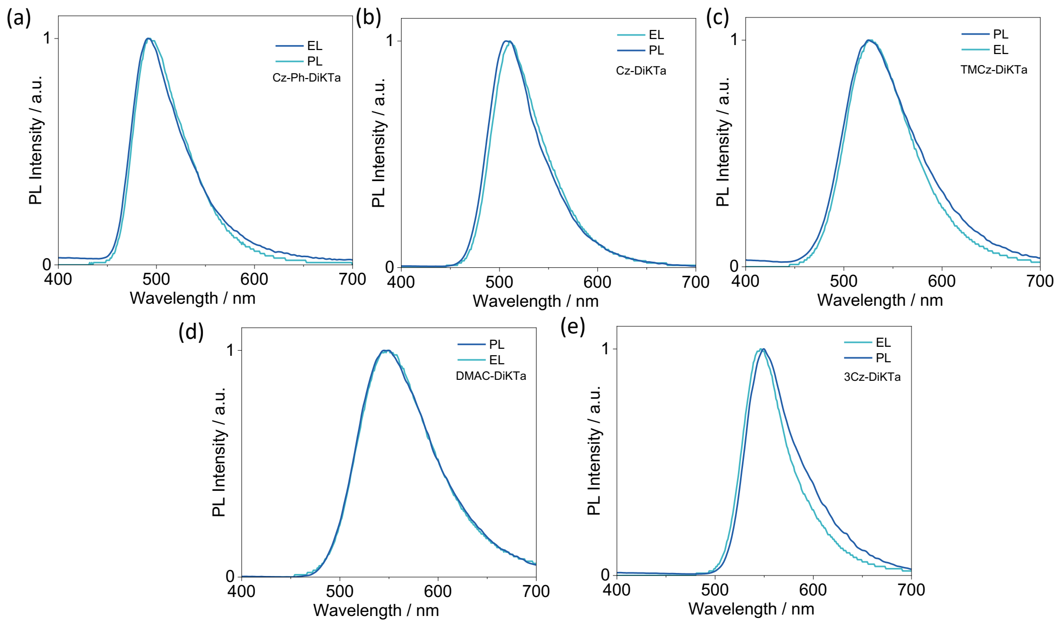

Figure **S16.** The comparation between the EL and steady-state PL spectrum of (a) **Cz-Ph-DiKTa**, (b) **Cz-DiKTa**, (c) **TMCz-DiKTa**, (d) **DMAC-DiKTa** and (e) **3Cz-DiKTa**.

Table **S8**. Device metrics for OLEDs employing the emitters based on carbonyl/amine type MR-TADF emitters.

Emitter	V_{on} [a)] / V	EQE_{max} [b)] / %	EQE_{1000} [c)] / %	λ_{EL} [d)] / nm	FWHM [e)] / nm	Ref
Cz-DiKTa	3.2	24.9	13.0	511	62	This work
Cz-Ph-DiKTa	3.2	23.0	10.2	492	61	
TMCz-DiKTa	3.1	20.2	16.7	527	78	
DMAC-DiKTa	3.1	23.8	19.9	549	89	
3Cz-DiKTa	3.2	24.4	6.2	547	54	
Mes_3DiKTa	3.0	21.1	4.5	480	36	[8]
QAD-Cz	N/A	20.3	0.73	494	57	[11]

QAD-2Cz	N/A	27.3	12.4	530	56	[11]
QAD-mTDPA	N/A	26.3	4.7	589	67	[11]
DDiKTa	3.7	19.0	N/A	500	59	[7]
OQAO	N/A	20.3	N/A	532	34	[12]
DQAO	N/A	17.8	N/A	564	45	[12]
QA-PCZ	3.4	17.5	7.6	482	29	[13]
QA2	N/A	19.0	N/A	463	37	[14]
QA3	N/A	18.6	N/A	515	67	[14]
mBDPA-TOAT	N/A	17.3	N/A	600	45	[15]

a) V_{on}=Turn-on voltage; b) EQE_{max}=Maximum external quantum effciency; c) EQE_{1000}=EQE at 1000 cd m^{-2} d) EL = electroluminescence. E) FWHM = full width at half maximum.

Determination of emitter dipole orientation of new TADF emitter based on DiKTa

First, to test our measurement setup, films of *N,N′*-di(1-naphthyl)-*N,N′*-diphenyl-(1,1′-biphenyl)-4,4′-diamine (NPB) doped with bis(2-methyldibenzo[f,h]quinoxaline)(acetylacetonate)iridium(III) ($Ir(MDQ)_2acac$) fabricated by different methods, thermal evaporation and spin-coating, were measured. This is because the dipole ratio for this film has already been reported by many groups.[3, 16] NPB doped films with $Ir(MDQ)_2acac$ at a doping concentration of 5 wt% fabricated by thermal evaporation was tested. The film was measured to be 42nm thick by the ellipsometer.

Figure **S17a** shows a comparison of the simulated and measured data of the evaporated film on s-polarization. Several simulations with different film thicknesses were fitted

to the data. The best fit was obtained with a 42 nm thickness, as measured with the ellipsometer. Figure **S17b** is the comparison between measured and simulated p-polarisation data for the evaporated film. A few simulated curves have been fitted to the data, corresponding to different anisotropy coefficients. The curve that fits best is the grey curve (a = 0.238), showing a mostly horizontal dipole orientation that is consistent with literature values 0.23 - 0.25.[3] Figure **S17c** also shows the effect of repeatable measurements which includes removing and reloading. Whilst there is some slight variation, the alignment is still very good and the change to the estimated anisotropy coefficient is less than 0.1. This result show that reliability of our measurement set-up.

Next, NPB doped with Ir(MDQ)$_2$acac (6 wt%) film fabricated by spin coating, on a fused silica substrate were tested. The film was measured to be 29.3 nm thick by the ellipsometer. Figure **S17c** shows a comparison of the simulated and measured data of the spin coated film on s-polarization. Several simulations of different thicknesses were considered, with the best fit being 25 nm thickness. Spin coated films are less even than evaporated films so the thickness across the film may vary significantly by several nanometres. Figure **S17d** is the comparison between measured and simulated p-polarisation data for the spin coated film. The best fit is the grey curve a = 0.333 – suggesting near perfect isotropy which is expected for spin coated films. This is similar as the reported value of is 0.34- 0.35.[16] These result show that our set-up can measure the molecular orientation reproducibly and consistently with other groups.

Next, angular dependent PL of mCP films doped with new DiKTa core TADF emitters (7.5 wt%) were measured. mCP films doped with these TADF emitters experience significantly more photo degradation than NPB films. The film sensitivity to photo degradation is linked to its peak wavelength, with less stable samples emitting shorter

wavelengths, hence the red-emitting NPB films are very stable, whereas blue emitting **Cz-Ph-DiKTa** is quite unstable and degrades quickly. Thus, data points for the measurement were reduced compared with the NPB films. Figures **S18** and **S19** show measured angular distribution of the mCP films and its comparison with the simulated angular dependent PL. As shown in Figure **S18**, all the results are well simulated thicknesses which are close to the expected film thickness of 50 nm. From the fitting of the calculated results to the experimental result, the anisotropy factors were obtained as summarized in Table **S9**. All the new DiKTa core TADF emitter films exhibit similar dipole orientations factor, ~0.333 which corresponds to nearly isotropic dipole orientation.

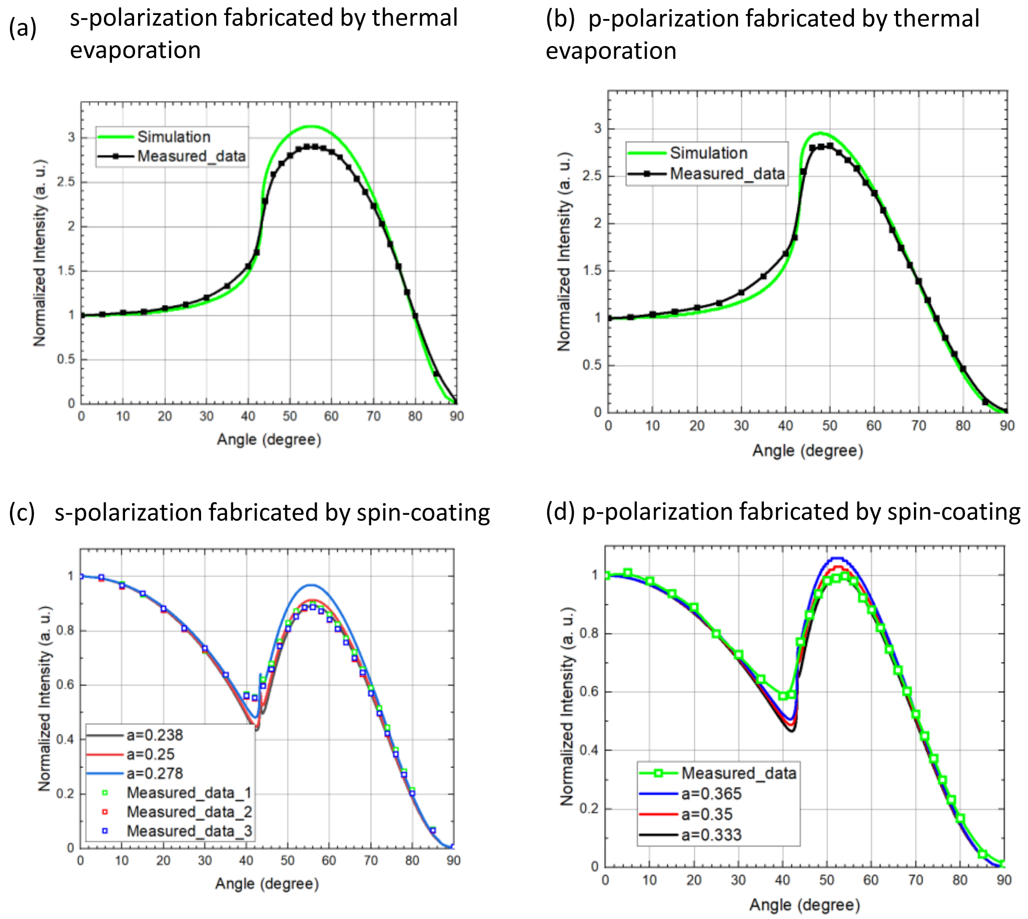

Figure **S17**. Measured polarization dependent PL intensity as a function of a rotation angle of NPB films doped with $Ir(MDQ)_2(acac)$ at different concentration fabricated by

different methods: (a) s- and (b) p-polarization fabricated by thermal evaporation and (c) s- and (d) p-polarization fabricated by spin-coating.

Table **S9**. Summary of determination of molecular orientation of NPB films doped with $Ir(MDQ)_2(acac)$ fabricated by different methods.

Doping concentration (wt%)	Fabrication Method	Measured Thickness (nm)	Simulated thickness (nm)	Anisotropy factor	Reported Anisotropy factor
5	Thermal evaporation	42	42	0.238	0.23 – 0.25 [3]
6	Spin coating	29.3	25	0.333	0.333 – 0.35 [16]

Table **S10**. Summary of determination of molecular orientation of new TADF emitters based on DiKTa core in mCP host. Nominal thickness of the films are 50 nm measured using a quartz crystal microbalance monitor.

Compounds	Simulated thickness (nm)	Peak wavelength (nm)	Anisotropy factor, a
**Cz-DiKTa**	48	509	0.33
**Cz-Ph-DiKTa**	50	494	0.36
**TMCz-DiKTa**	52	520	0.37
**DMAc-DiKTa**	53	545	0.36
**3Cz-DiKTa**	50	548	0.30

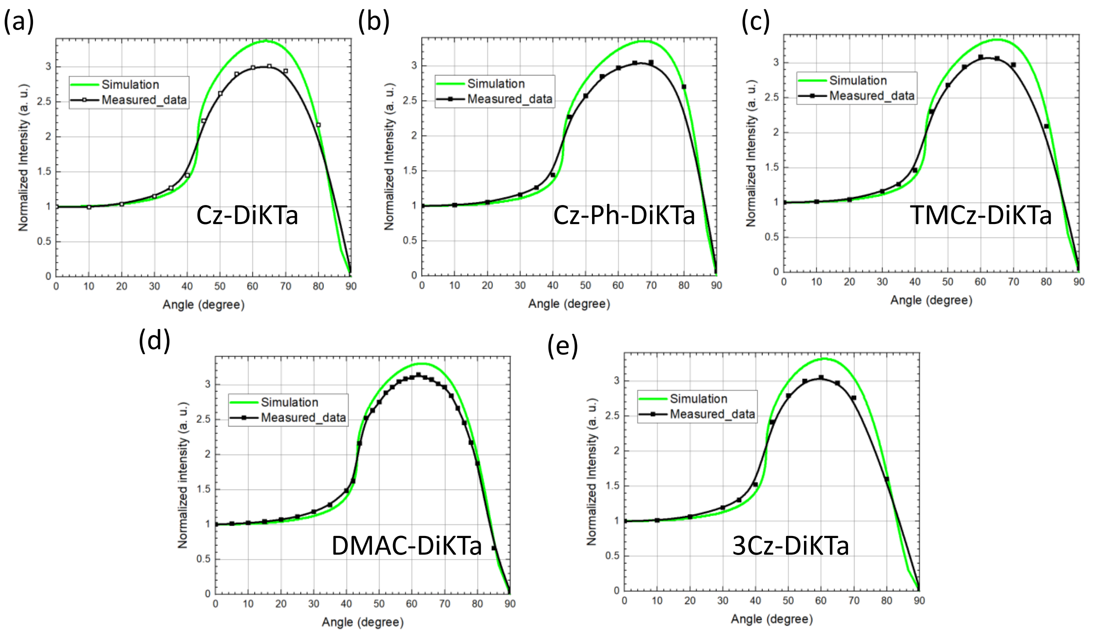

Figure **S18**. Measured PL intensity of s-polarized emission as a function of a rotation angle of mCP doped films with different emitters films at peak emission wavelength and comparison with simulated results: (a) **Cz-DiKTa**, (b) **Cz-Ph-DikTa**, (c) **TMCz-DiKTa**, (d) **DMAc-DiKTa**, and (e) **3Cz-DiKTa**

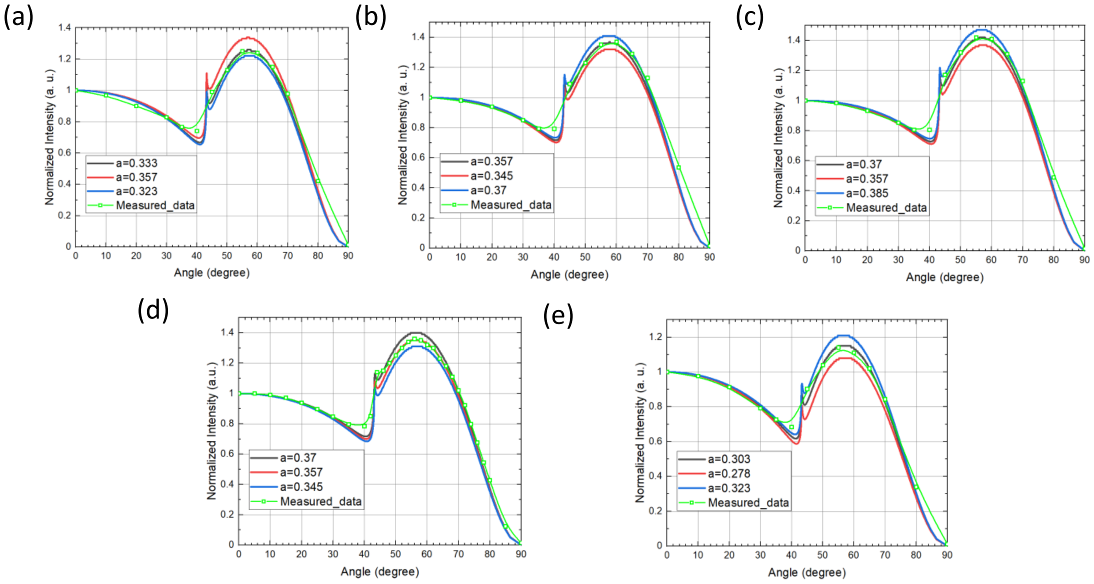

Figure **S19**. Measured PL intensity of p-polarized emission as a function of a rotation angle of mCP films doped with different emitters films at peak emission wavelength and comparison with simulated results: (a) **Cz-DiKTa**, (b) **Cz-Ph-DikTa**, (c) **TMCz-DiKTa**, (d) **DMAc-DiKTa**, and (e) **3Cz-DiKTa**

Out-coupling efficiency simulation and possible explanation about higher experimental EQE than predicted EQE

Table **S11** summarizes the calculated out-coupling efficiency of the OLEDs. Experimental EQE_{max} values were slightly higher than the predicted EQE_{max} values.

The possible reasons can be underestimation of emission efficiency of emission layer and/or overestimation of experimental EQE_{max} values. The details are as follows.

Underestimation of the emission efficiency of the emission layer could potentially occur due to emission quenching by oxygen during the measurements of the Φ_{PL}. Although the Φ_{PL} was measured under nitrogen flow conditions, oxygen may not have been completely removed from the integrating sphere used for the measurement. This environment is different from the emission layer inside the OLED stack. Thus, the emission efficiency of the emissive layer in the device stack could be higher than the measured Φ_{PL} of the films emulating the emission layer. In addition, in the OLED stack emission efficiency can be enhanced by the electromagnetic standing wave in the cavity;[4] this is the so-called the Purcell effect.

Overestimation of the EQE_{max} value could potentially happen because we measure light emitted in the forward direction and assume a Lambertian angular distribution of the emitted light. While OLEDs are usually assumed to be Lambertian emitters, the emission patterns can be different from Lambertian depending on the thickness of OLEDs.[5] This is applicable even for conventional bottom-emitting OLEDs based on ITO as the semi-transparent electrode. Based on the out-coupling simulation, some of our OLEDs will have slightly different emission pattern from Lambertian. For example, the OLEDs with **DMAc-DiKTa** and **3Cz-DiKTa** have more directed emission in the forward direction than Lambertian, thus experimental EQE_{max} values could be overestimated by up to 15%.

Table **S11**. Summary of out-coupling simulation of OLEDs.

Compound	Φ_{PL} [a] /%	Experimental EQE_{max} /%	Calculated Out-coupling efficiency /%	Predicted EQE_{max} /%
**Cz-DiKTa**	77	24.9	25.4	19.6
**Cz-Ph-DiKTa**	69	23.0	24.4	16.8
**TMCz-DiKTa**	57	20.2	23.2	13.1
**DMAc-DiKTa**	62	23.8	23.0	14.3
**3Cz-DiKTa**	74	24.4	25.4	18.8

[a] Measured in vacuum-deposited 7.5 wt% doped mCP films.

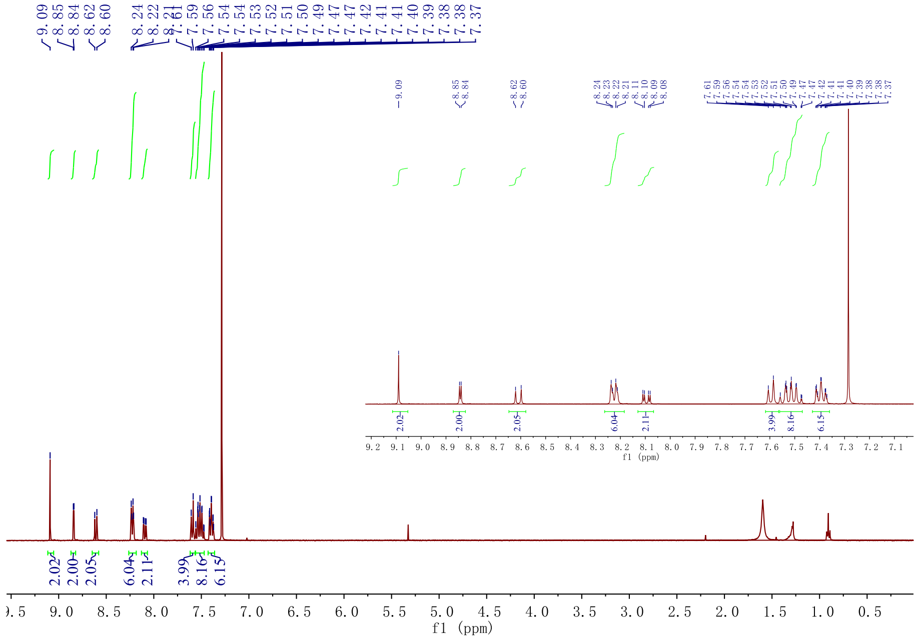

Figure **S20.** ^{1}H-NMR of **3Cz-DiKTa** in $CDCl_3$.

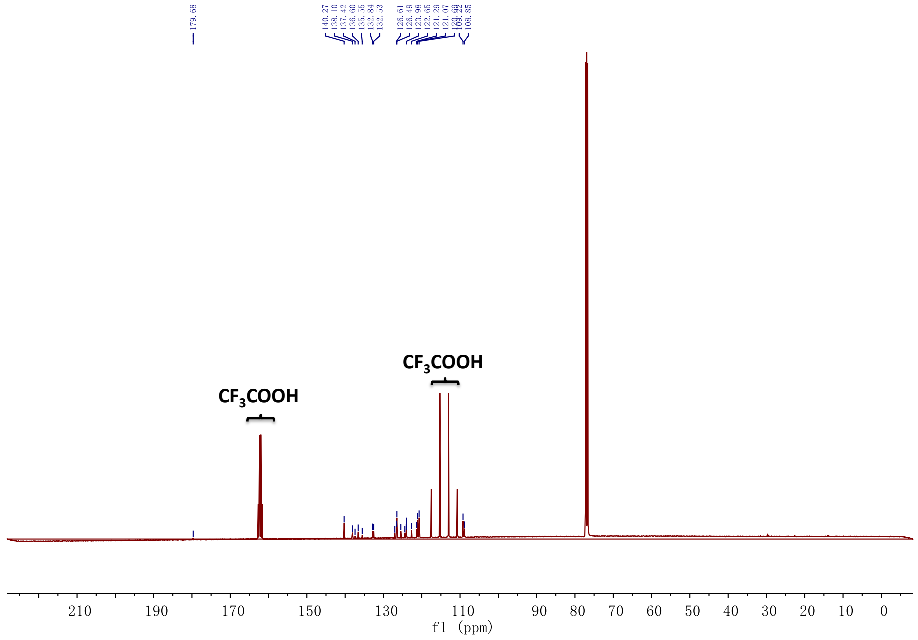

Figure **S21.** ^{13}C-NMR of **3Cz-DiKTa** in $CDCl_3$

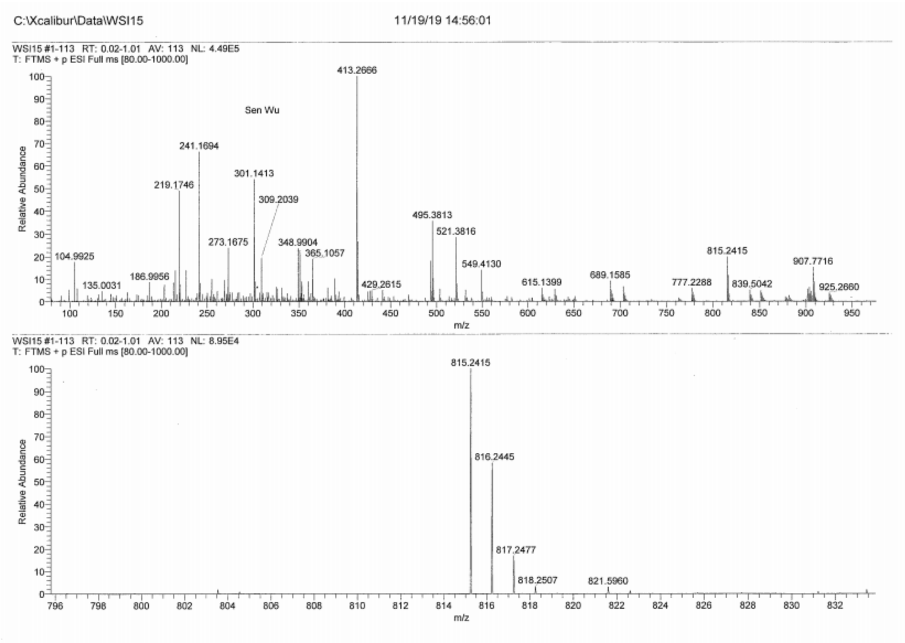

Figure **22.** HRMS of **3Cz-DiKTa**.

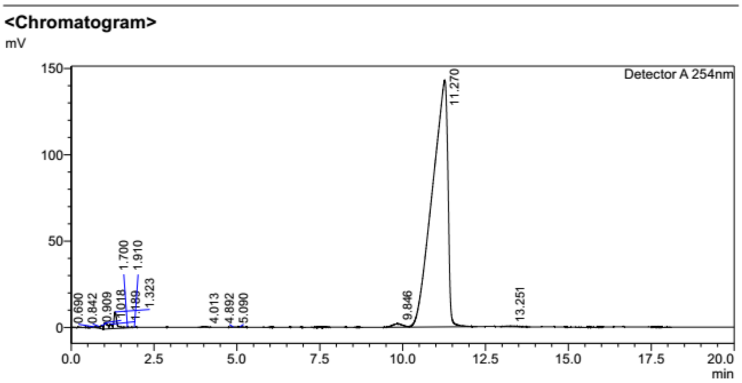

<Peak Table>

Detector A 254nm

Peak#	Ret. Time	Area	Height	Area%	Area/Height	Width at 5% Height
1	0.690	3278	494	0.067	6.629	0.179
2	0.842	3778	1034	0.078	3.653	--
3	0.909	7772	1692	0.160	4.594	--
4	1.018	25223	3510	0.519	7.185	--
5	1.189	14507	2676	0.298	5.420	--
6	1.323	58369	9599	1.201	6.081	--
7	1.700	5123	476	0.105	10.773	--
8	1.910	2173	213	0.045	10.208	--
9	4.013	5372	542	0.110	9.908	0.312
10	4.892	2394	173	0.049	13.862	--
11	5.090	2907	267	0.060	10.897	--
12	9.846	41524	1873	0.854	22.172	--
13	11.270	4678028	142900	96.224	32.736	1.048
14	13.251	11139	437	0.229	25.472	0.761
Total		4861586	165886	100.000		

Figure **23.** HPLC report of **3Cz-DiKTa**.

Elemental Analysis Service Request Form

Researcher name Sen Wu

Researcher email ws60@st-andrews.ac.uk

NOTE: Please submit ca. 10 mg of sample

**Sample reference number**	WS-714
**Name of Compound**	3Cz-DiKTa.
**Molecular formula**	$C_{56}H_{32}N_4O_2$
**Stability**	
**Hazards**	
**Other Remarks**	

Analysis type:

Single ☐ **Duplicate** ☑ **Triplicate** ☐

Analysis Result:

Element	Expected %	Found (1)	Found (2)	Found (3)
Carbon	84.83	84.54	84.31	
Hydrogen	4.07	4.16	4.01	
Nitrogen	7.07	6.34	6.91	
Oxygen				

Authorising Signature:

**Date completed**	21.05.21
**Signature**	[illegible]
**comments**	

Figure **24.** Elemental analysis report of **3Cz-DiKTa**.

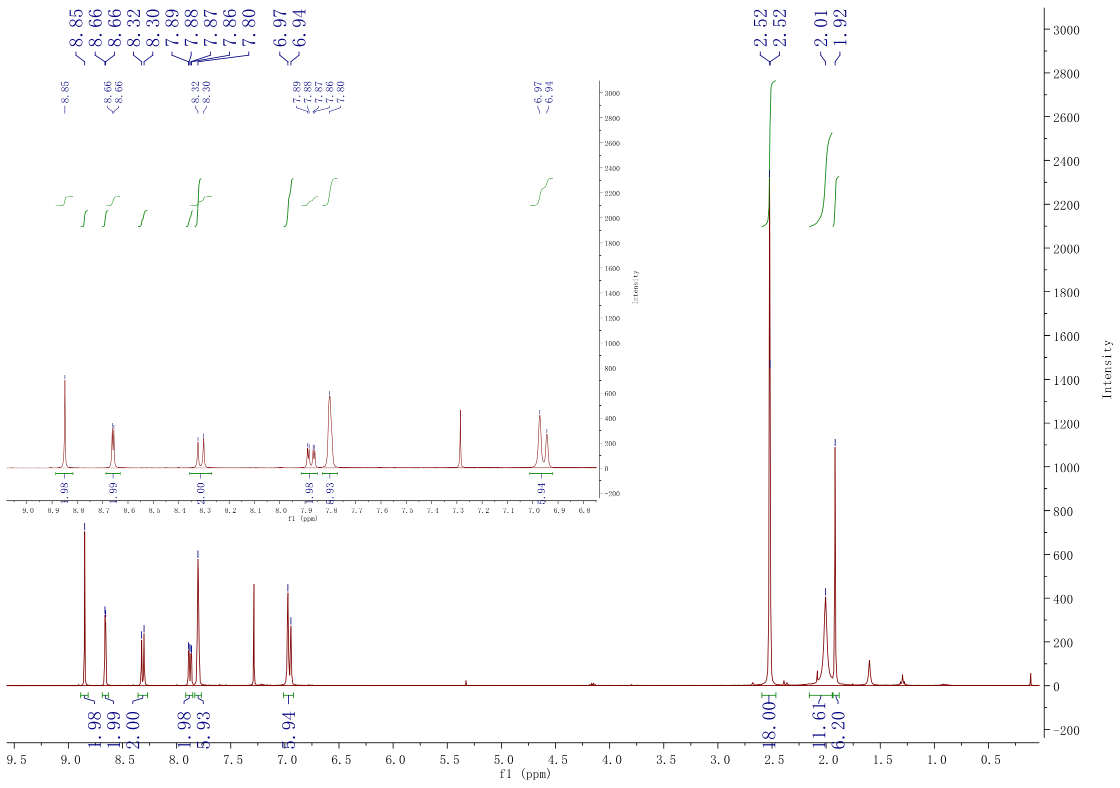

Figure **S25.** ^{1}H-NMR of **3TMCz-DiKTa** in $CDCl_3$.

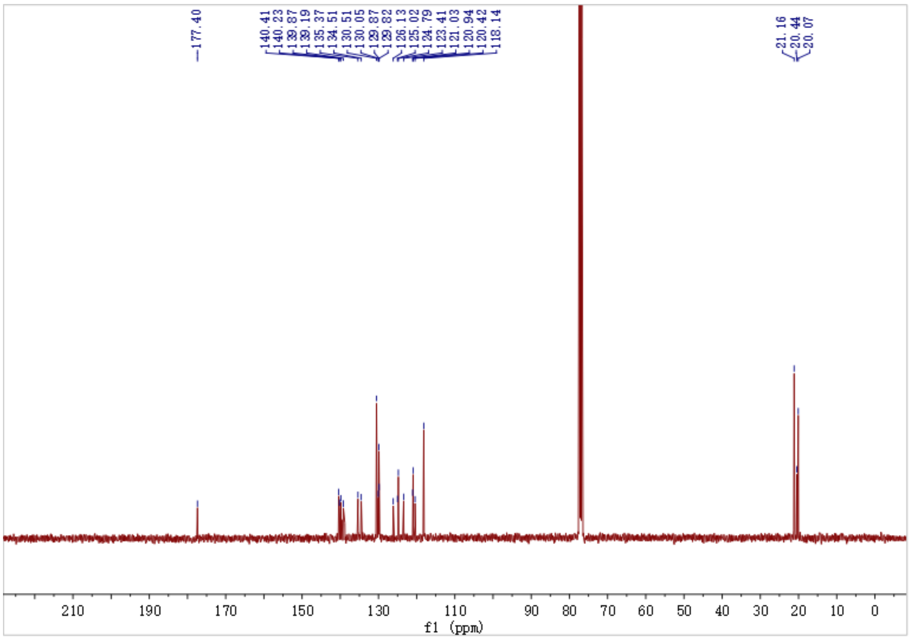

Figure **S26.** ^{13}C-NMR of **3TMCz-DiKTa** in $CDCl_3$.

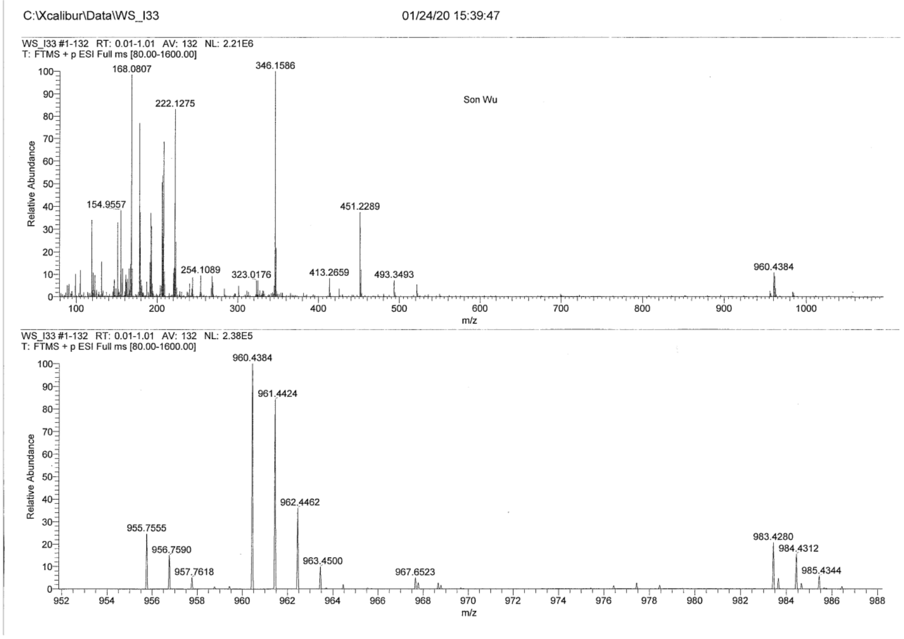

Figure **S27.** HRMS of **3TMAC-DiKTa**.

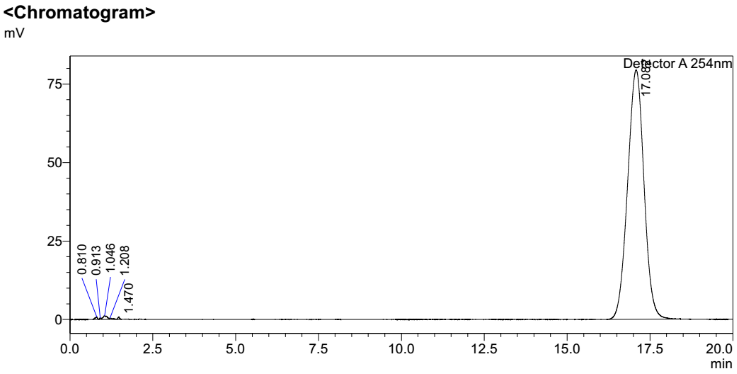

<Peak Table>

Detector A 254nm

Peak#	Ret. Time	Area	Height	Area%	Area/Height	Width at 5% Height
1	0.810	4093	789	0.149	5.188	0.175
2	0.913	1645	469	0.060	3.509	--
3	1.046	10236	1181	0.372	8.667	--
4	1.208	2991	392	0.109	7.633	--
5	1.470	2714	762	0.099	3.562	--
6	17.082	2727175	79434	99.211	34.332	1.142
Total		2748855	83027	100.000		

Figure **S28.** HPLC report of **3TMCZ-DiKTa**.

Elemental Analysis Service Request Form

Researcher name Sen Wu

Researcher email ws60@st-andrews.ac.uk

NOTE: Please submit ca. 10 mg of sample

**Sample reference number**	WS-I44
**Name of Compound**	3TMCz-DiKTa
**Molecular formula**	C68H56N4O2
**Stability**	
**Hazards**	
**Other Remarks**	

Analysis type:

Single ☐ **Duplicate** ☑ **Triplicate** ☐

Analysis Result:

Element	Expected %	Found (1)	Found (2)	Found (3)
Carbon	84.97	~~81.66~~ 84.02	~~77.80~~ 83.78	
Hydrogen	5.87	6.08	6.29	
Nitrogen	5.83	~~5.29~~ 5.70	~~4.95~~ 5.68	
Oxygen				

Authorising Signature:

**Date completed**	06.10.21
**Signature**	J -P L
**comments**	

Figure **S29.** Elemental analysis report of **3TMCZ-DiKTa**.

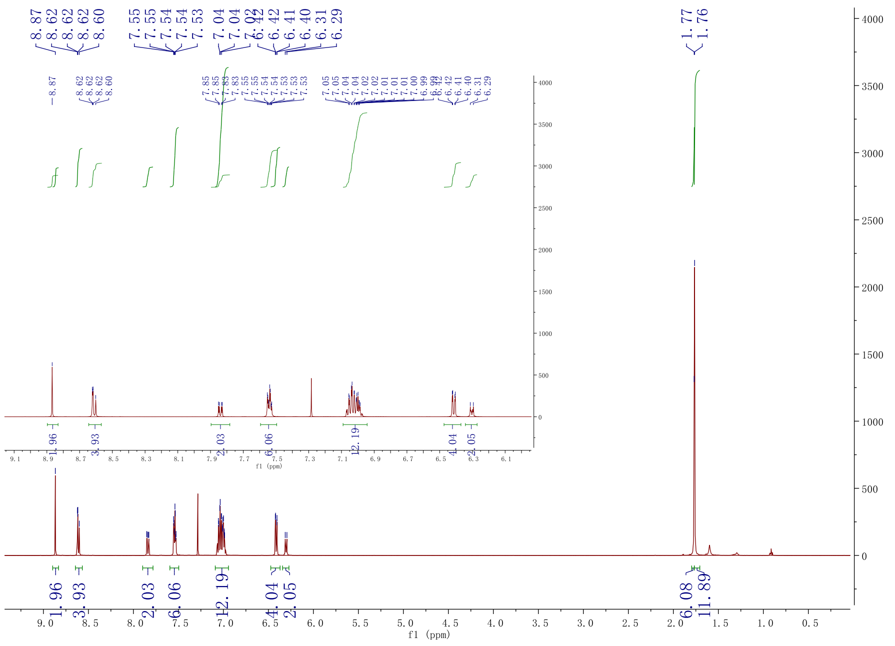

Figure **S30.** ^{1}H-NMR of **3DMAC-DiKTa** in $CDCl_3$.

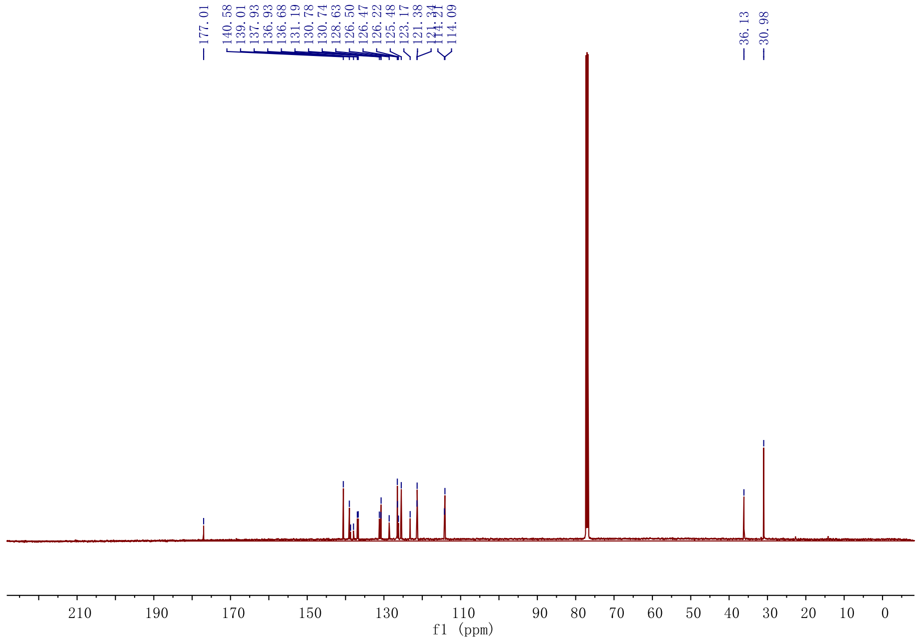

Figure **S31.** ^{13}C-NMR of **3DMAC-DiKTa** in $CDCl_3$.

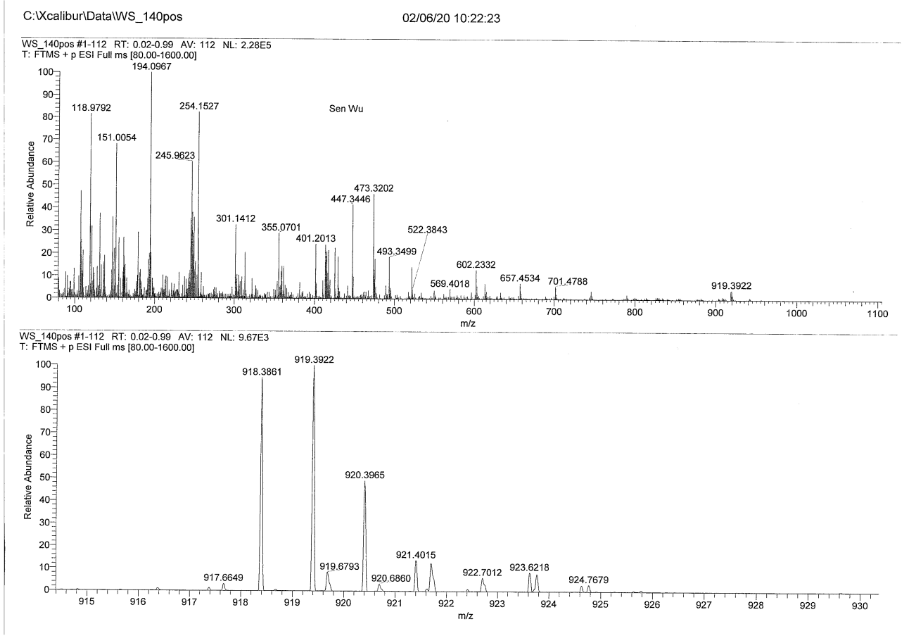

Figure **S32.** HRMS of **3DMAC-DiKTa**.

<Chromatogram>

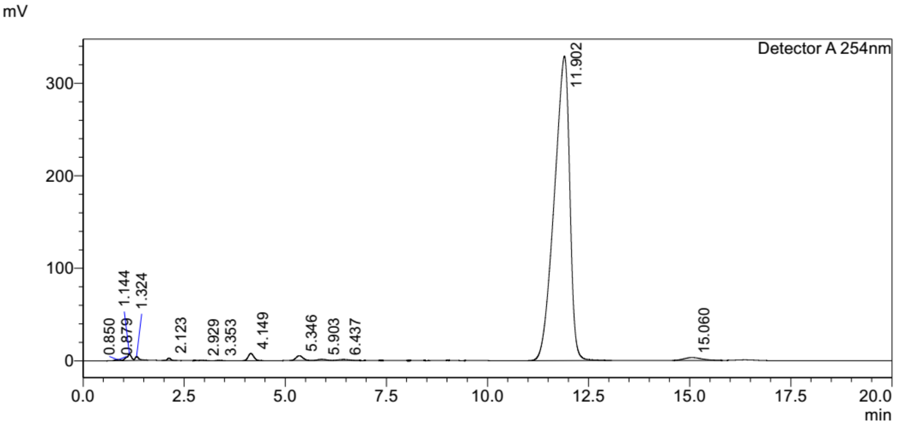

<Peak Table>

Detector A 254nm

Peak#	Ret. Time	Area	Height	Area%	Area/Height	Width at 5% Height
1	0.850	6241	1314	0.068	4.748	--
2	0.879	6459	1308	0.071	4.937	--
3	1.144	56968	6913	0.622	8.241	--
4	1.324	31044	4228	0.339	7.343	--
5	2.123	15481	2663	0.169	5.814	0.237
6	2.929	3673	461	0.040	7.968	0.252
7	3.353	2646	412	0.029	6.429	0.211
8	4.149	75121	7797	0.821	9.635	0.320
9	5.346	61941	5187	0.677	11.941	0.391
10	5.903	21160	1421	0.231	14.893	--
11	6.437	24270	1205	0.265	20.135	--
12	11.902	8753043	328975	95.612	26.607	0.878
13	15.060	96690	3112	1.056	31.068	1.031
Total		9154735	364997	100.000		

Figure **S33.** HPLC report of **3DMAC-DiKTa**.

NOTE: Please submit ca. 10 mg of sample

**Sample reference number**	WS-240
**Name of Compound**	3DMAC DiKTa.
**Molecular formula**	$C_{65}H_{50}N_4O$
**Stability**	
**Hazards**	
**Other Remarks**	

Analysis type:

Single ☐ **Duplicate** ☒ **Triplicate** ☐

Analysis Result:

Element	Expected %	Found (1)	Found (2)	Found (3)
Carbon	84.94	84.16	84.21	
Hydrogen	5.48	5.23	5.10	
Nitrogen	6.1	5.90	5.87	
Oxygen				

Authorising Signature:

**Date completed**	JPL 10.12.20 ~~12.10.20~~ 10.12.20
**Signature**	J-PL
**comments**	

Figure **S34.** Elemental analysis report of **3DMAC-DiKTa**.

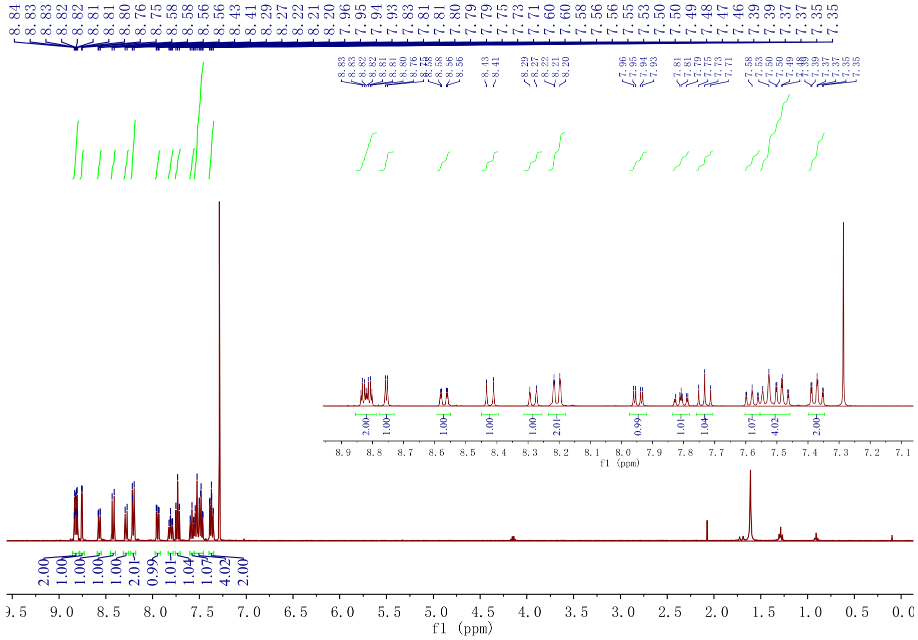

Figure **S35.** ^{1}H-NMR of **Cz-DiKTa** in CD_2Cl_2.

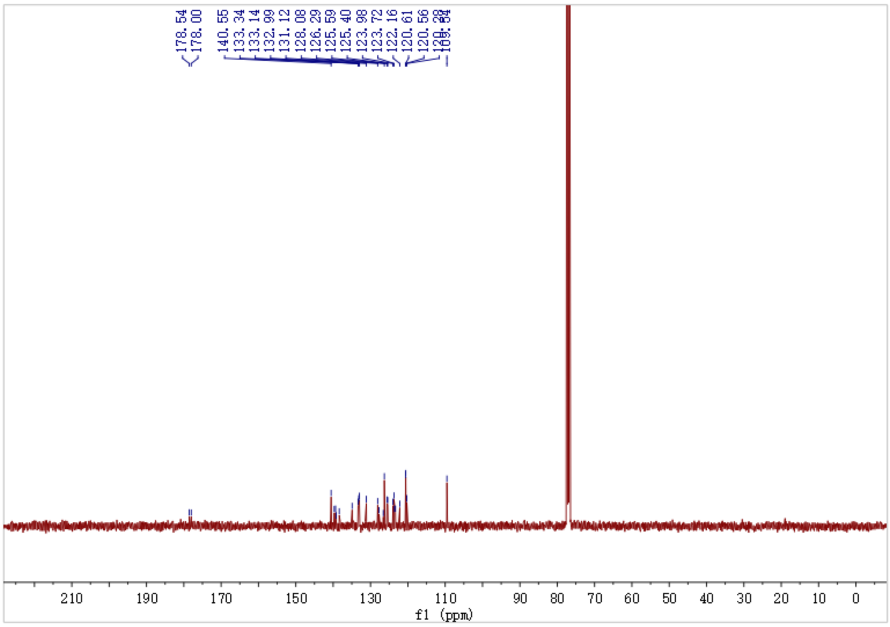

Figure **S36.** ^{13}C-NMR of **Cz-DiKTa** in $CDCl_3$.

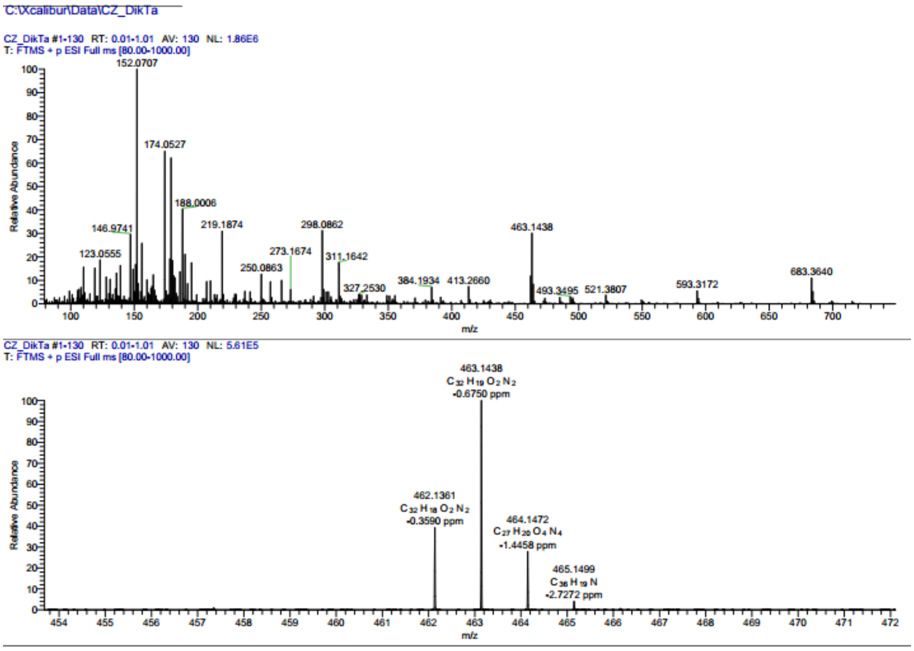

Figure **S37.** HRMS of **Cz-DiKTa**.

<Chromatogram>

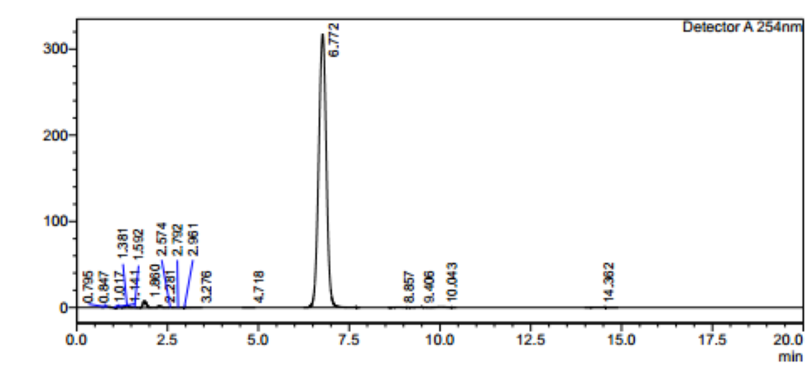

<Peak Table>

Detector A 254nm

Peak#	Ret. Time	Area	Height	Area%	Area/Height	Width at 5% Height
1	0.795	3371	749	0.070	4.500	--
2	0.847	4429	713	0.093	6.213	--
3	1.017	1850	339	0.039	5.449	--
4	1.141	11226	1434	0.235	7.829	--
5	1.381	21793	2487	0.456	8.761	--
6	1.592	6244	602	0.131	10.372	--
7	1.860	56225	7547	1.176	7.450	--
8	2.281	15830	1997	0.331	7.928	--
9	2.574	4564	442	0.095	10.316	--
10	2.792	1586	191	0.033	8.317	--
11	2.961	2604	247	0.054	10.528	--
12	3.276	1149	109	0.024	10.542	--
13	4.718	1008	95	0.021	10.643	0.289
14	6.772	4617214	316929	96.535	14.569	0.476
15	8.857	6802	408	0.142	16.652	--
16	9.406	7883	465	0.165	16.958	--
17	10.043	12400	648	0.259	19.123	0.598
18	14.362	6782	269	0.142	25.207	0.758
Total		4782960	335672	100.000		

Figure **S38.** HPLC report of **Cz-DiKTa**.

NOTE: Please submit ca. 10 mg of sample

**Sample reference number**	WS-I62
**Name of Compound**	Cz-DIKTA
**Molecular formula**	C32H18N2O2
**Stability**	
**Hazards**	
**Other Remarks**	

Analysis type:

Single ☐ **Duplicate** ☑ **Triplicate** ☐

Analysis Result:

Element	Expected %	Found (1)	Found (2)	Found (3)
Carbon	83.10	81.79	83.38	
Hydrogen	3.92	3.91	4.02	
Nitrogen	6.06	5.91	6.07	
Oxygen				

Authorising Signature:

**Date completed**	21.05.21
**Signature**	J-P C
**comments**	

Figure **S39.** Elemental analysis report of **Cz-DiKTa**.

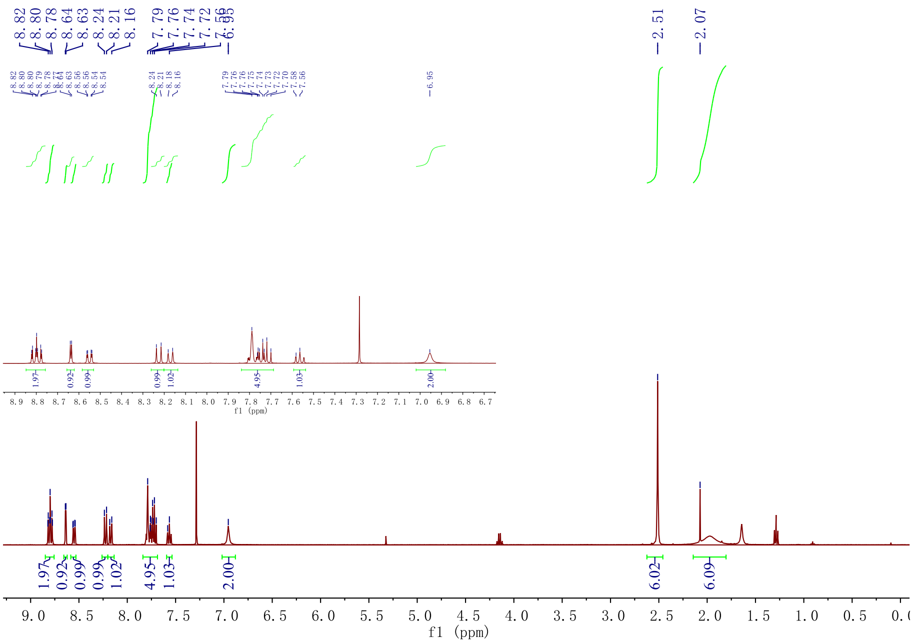

Figure **S40.** ^{1}H-NMR of **TMCz-DiKTa** in $CDCl_3$.

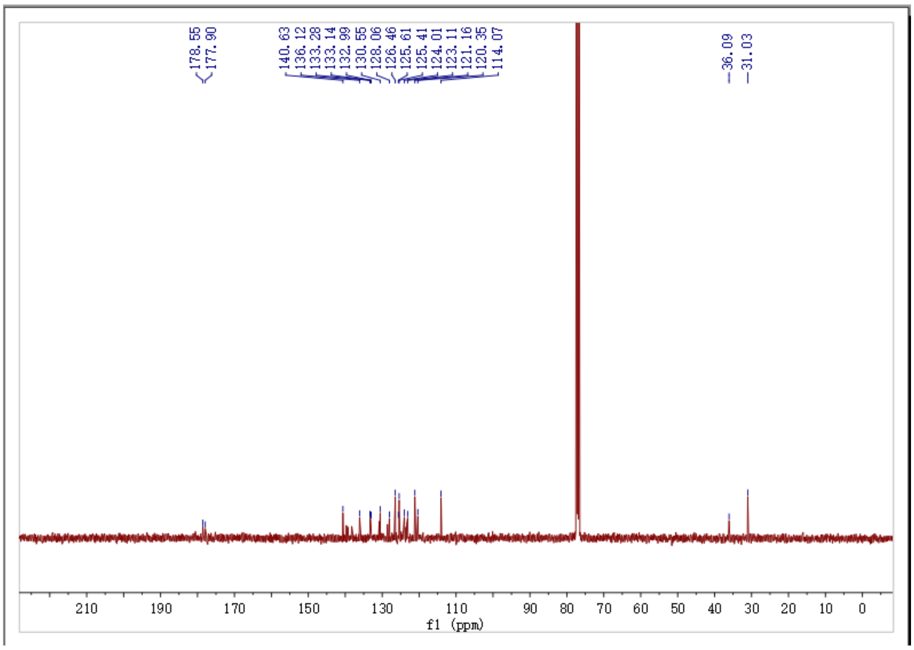

Figure **S41.** ^{13}C-NMR of **TMCz-DiKTa** in $CDCl_3$.

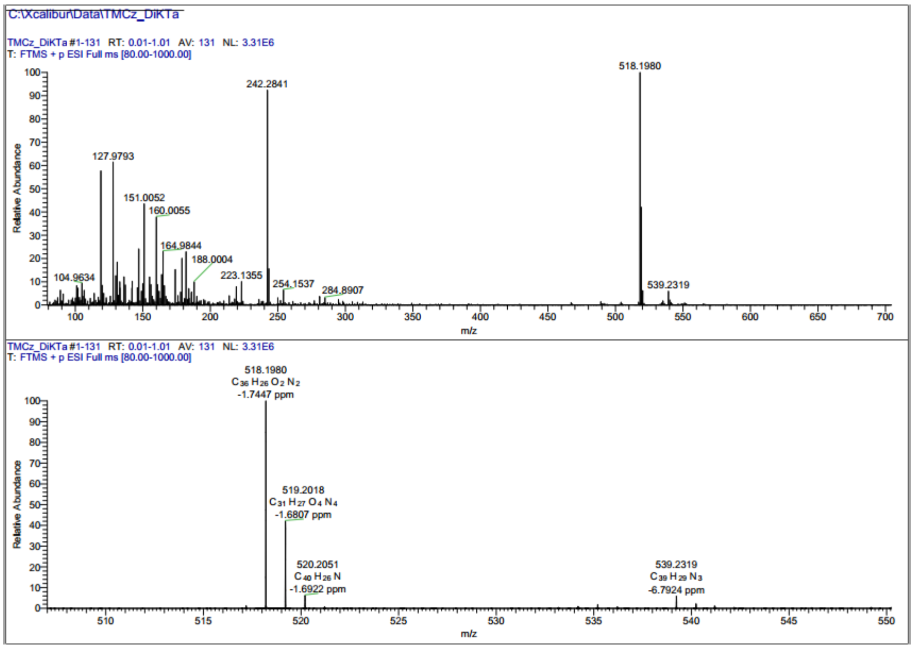

Figure **S42.** HRMS report of **TMCz-DiKTa**.

<Chromatogram>

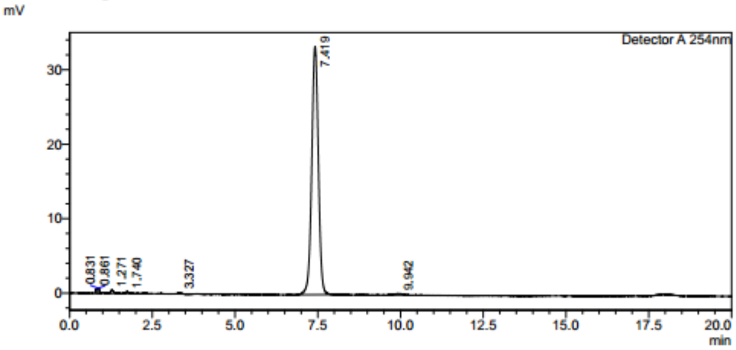

<Peak Table>

Detector A 254nm

Peak#	Ret. Time	Area	Height	Area%	Area/Height	Width at 5% Height
1	0.831	2653	739	0.554	3.590	--
2	0.861	2754	680	0.575	4.049	--
3	1.271	2893	471	0.604	6.138	--
4	1.740	1207	250	0.252	4.826	0.156
5	3.327	1278	176	0.267	7.244	0.214
6	7.419	464493	33298	97.038	13.950	0.474
7	9.942	3393	192	0.709	17.691	0.521
Total		478671	35807	100.000		

Figure **S43.** HPLC report of **TMCz-DiKTa**.

NOTE: Please submit ca. 10 mg of sample

**Sample reference number**	WS-I72
**Name of Compound**	TMCZ-DIKTA
**Molecular formula**	C36H26N2O2
**Stability**	
**Hazards**	
**Other Remarks**	

Analysis type:

Single ☐ **Duplicate** ☑ **Triplicate** ☐

Analysis Result:

**Element**	**Expected %**	**Found (1)**	**Found (2)**	**Found (3)**
**Carbon**	83.37	82.68	83.34	
**Hydrogen**	5.05	5.00	4.97	
**Nitrogen**	5.40	5.17	5.33	
**Oxygen**				

Authorising Signature:

**Date completed**	20.11.20
**Signature**	J-DL
**comments**	

Figure **S44.** Elemental analysis report of **TMCz-DiKTa**.

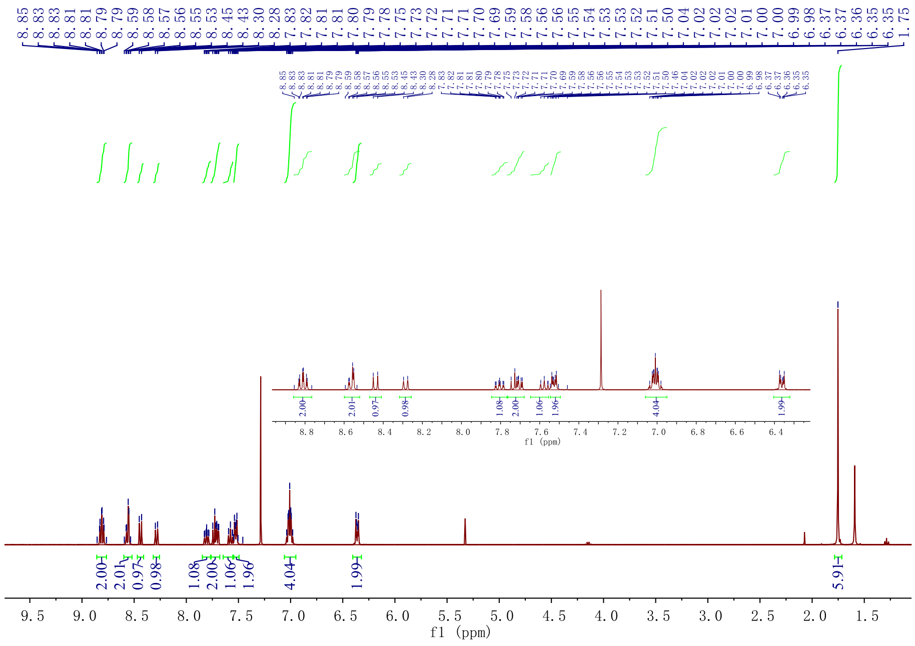

Figure **S45.** ^{1}H-NMR of **DMAC-DiKTa** in $CDCl_3$.

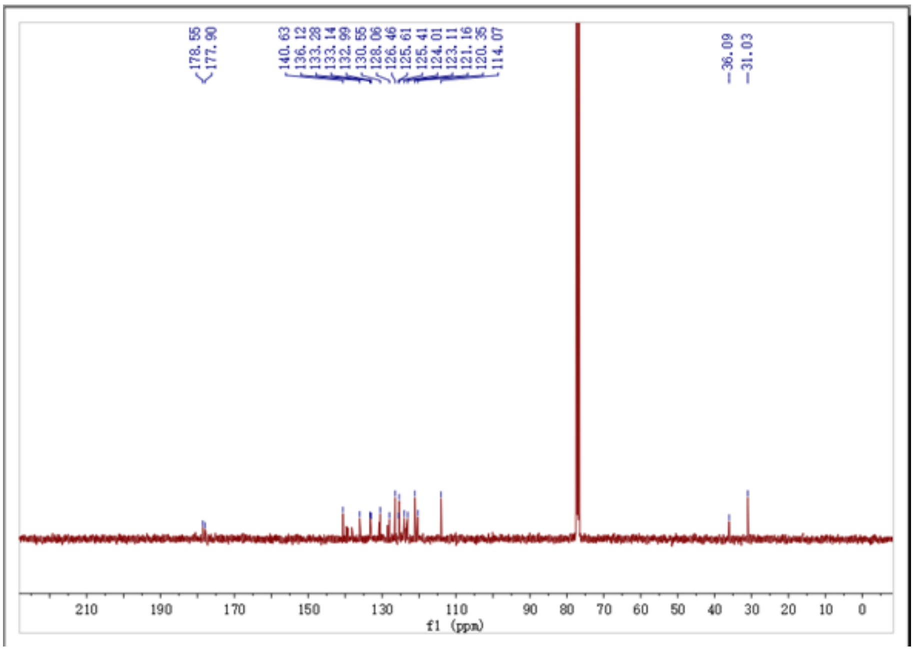

Figure **S46.** ^{13}C-NMR of **DMAC-DiKTa** in $CDCl_3$.

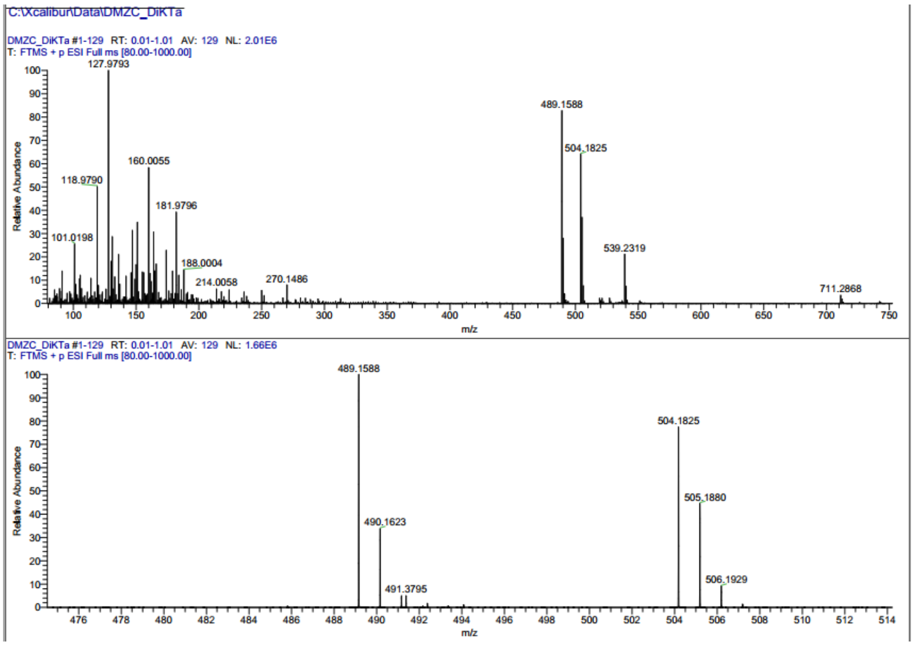

Figure **S47.** HRMS report of **DMAC-DiKTa** in $CDCl_3$.

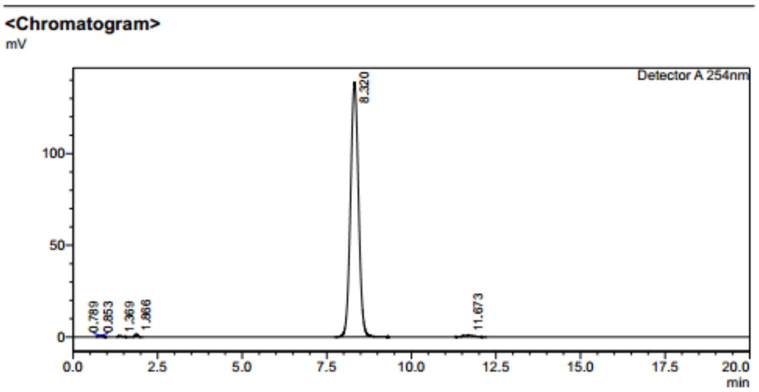

<Peak Table>

Detector A 254nm

Peak#	Ret. Time	Area	Height	Area%	Area/Height	Width at 5% Height
1	0.789	4110	779	0.177	5.274	--
2	0.853	4539	800	0.195	5.673	--
3	1.369	5047	749	0.217	6.735	0.284
4	1.866	8914	1516	0.383	5.880	0.183
5	8.320	2286157	138776	98.183	16.474	0.548
6	11.673	19708	865	0.846	22.784	0.729
Total		2328476	143485	100.000		

Figure **S48.** HPLC report of **DMAC-DiKTa** in $CDCl_3$.

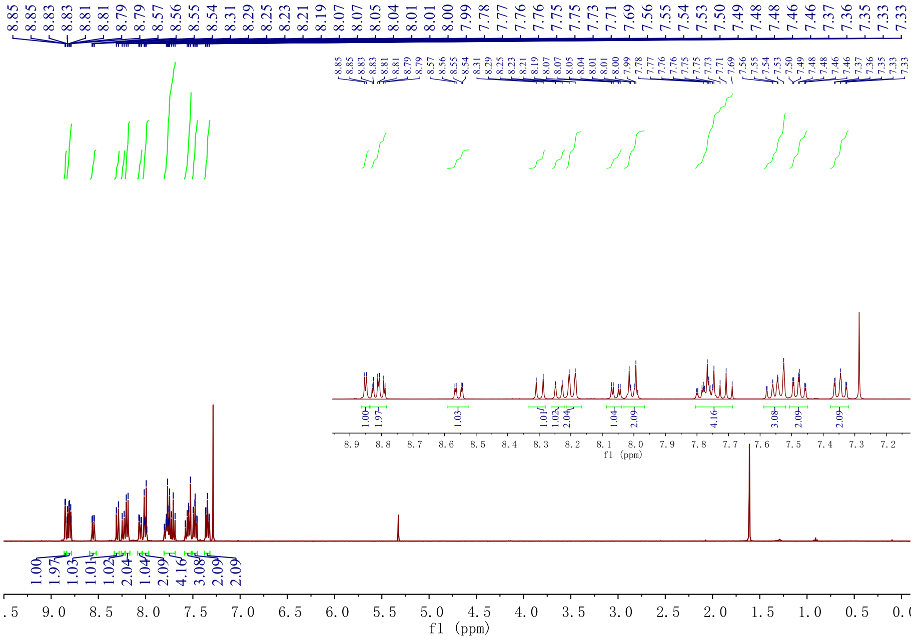

Figure **S49.** ^{1}H-NMR of **Cz-Ph-DiKTa** in $CDCl_3$.

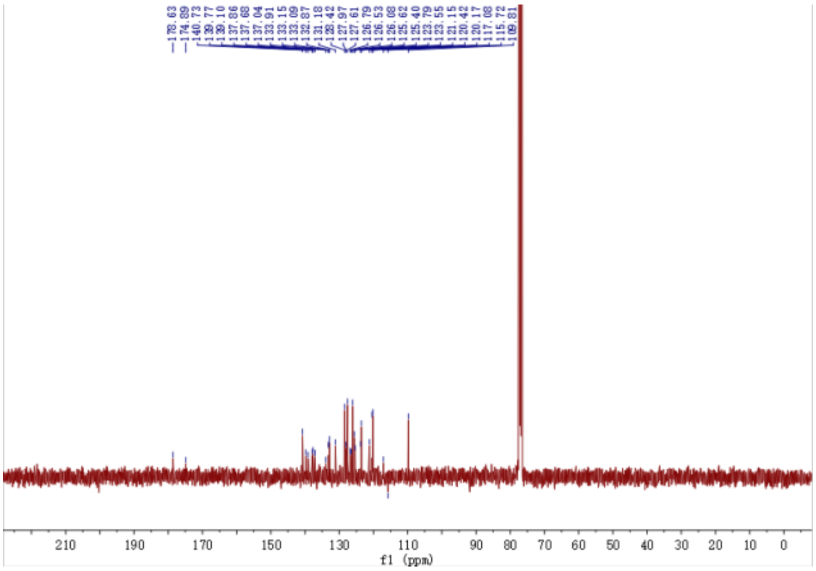

Figure **S50.** ^{13}C-NMR of **Cz-Ph-DiKTa** in $CDCl_3$.

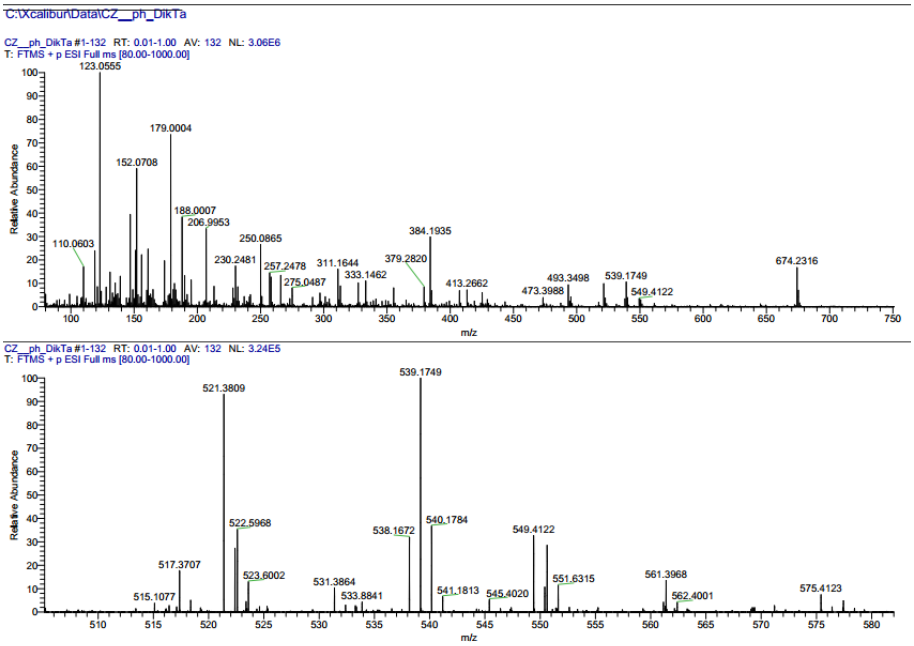

Figure **S51.** HRMS report of **Cz-Ph-DiKTa**.

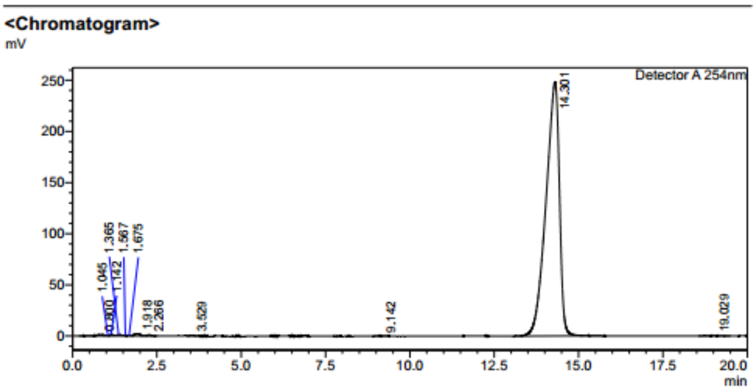

<Peak Table>

Detector A 254nm

Peak#	Ret. Time	Area	Height	Area%	Area/Height	Width at 5% Height
1	0.800	20194	1427	0.288	14.154	--
2	1.045	3565	580	0.051	6.150	--
3	1.142	9905	1258	0.141	7.872	--
4	1.365	11348	1169	0.162	9.708	--
5	1.567	2815	477	0.040	5.901	--
6	1.675	2142	388	0.031	5.518	--
7	1.918	26035	2231	0.372	11.672	--
8	2.266	7315	1133	0.104	6.458	--
9	3.529	6864	760	0.098	9.030	0.285
10	9.142	2801	195	0.040	14.374	0.418
11	14.301	6904257	248278	98.542	27.809	0.933
12	19.029	9194	321	0.131	28.659	0.833
Total		7006435	258216	100.000		

Figure **S52.** HPLC report of **Cz-Ph-DiKTa**.

NOTE: Please submit ca. 10 mg of sample

**Sample reference number**	WS-I67
**Name of Compound**	Cz-Ph-DIKTA
**Molecular formula**	C38H22N2O2
**Stability**	
**Hazards**	
**Other Remarks**	

Analysis type:

Single ☐ **Duplicate** ☑ **Triplicate** ☐

Analysis Result:

Element	Expected %	Found (1)	Found (2)	Found (3)
Carbon	84.74	84.29	83.24	
Hydrogen	4.12	4.18	4.11	
Nitrogen	5.20	5.15	5.15	
Oxygen				

Authorising Signature:

**Date completed**	21.05.21
**Signature**	J-P L
**comments**	

Figure **S53.** Elemental analysis report of **Cz-Ph-DiKTa**.

References

(1) Wasey, J.; Safonov, A.; Samuel, I.; Barnes, W. Effects of Dipole Orientation and Birefringence on the Optical Emission from Thin Films. *Opt. Commun.* **2000**, *183* (1-4), 109-121.

(2) Frischeisen, J.; Yokoyama, D.; Adachi, C.; Brütting, W. Determination of Molecular Dipole Orientation in Doped Fluorescent Organic Thin Films by Photoluminescence Measurements. *Appl. Physl. Lett.* **2010**, *96* (7), 073302.

(3) Schmidt, T. D.; Lampe, T.; Djurovich, P. I.; Thompson, M. E.; Brütting, W. Emitter Orientation as a Key Parameter in Organic Light-Emitting Diodes. *Phys. Rev. Appl.* **2017**, *8* (3), 037001.

(4) Nowy, S.; Krummacher, B. C.; Frischeisen, J.; Reinke, N. A.; Brütting, W. Light Extraction and Optical Loss Mechanisms in Organic Light-Emitting Diodes: Influence of the Emitter Quantum Efficiency. *J. Appl. Phys.* **2008**, *104* (12), 123109.

(5) Furno, M.; Meerheim, R.; Hofmann, S.; Lüssem, B.; Leo, K. Efficiency and Rate of Spontaneous Emission in Organic Electroluminescent Devices. *Phys. Rev. B* **2012**, *85* (11), 115205.

(6) Neyts, K. A. Simulation of Light Emission from Thin-Film Microcavities. *J. Opt. Soc. An. A.* **1998**, *15* (4), 962-971.

(7) Sun, D.; Suresh, S. M.; Hall, D.; Zhang, M.; Si, C.; Cordes, D. B.; Slawin, A. M. Z.; Olivier, Y.; Zhang, X.; Zysman-Colman, E. The Design of an Extended Multiple Resonance TADF Emitter Based on a Polycyclic Amine/Carbonyl System. *Mater. Chem. Front.* **2020**, *4* (7), 2018-2022.

(8) Hall, D.; Suresh, S. M.; dos Santos, P. L.; Duda, E.; Bagnich, S.; Pershin, A.; Rajamalli, P.; Cordes, D. B.; Slawin, A. M. Z.; Beljonne, D.; et al. Improving Processability and Efficiency of Resonant TADF Emitters: A Design Strategy. *Adv. opt. Mater.* **2020,** *8*(2), 1901627.

(9) Connelly, N. G.; Geiger, W. E. J. C. R. Chemical Redox Agents for Organometallic Chemistry. *Chem. Rev.* **1996**, *96* (2), 877-910.

(10) Cardona, C. M.; Li, W.; Kaifer, A. E.; Stockdale, D.; Bazan, G. C. Electrochemical Considerations for Determining Absolute Frontier Orbital Energy Levels of Conjugated Polymers for Solar Cell Applications. *Adv. Mater.* **2011**, *23* (20), 2367-2371.

(11) Huang, F.; Wang, K.; Shi, Y.-Z.; Fan, X.-C.; Zhang, X.; Yu, J.; Lee, C.-S.; Zhang, X.-H. Approaching Efficient and Narrow RGB Electroluminescence from D–A-Type TADF Emitters Containing an Identical Multiple Resonance Backbone as the Acceptor. *ACS Appl. Mater. Interfaces* **2021**, *13* (30), 36089-36097.

(12) Zou, S. N.; Peng, C. C.; Yang, S. Y.; Qu, Y. K.; Yu, Y. J.; Chen, X.; Jiang, Z. Q.; Liao, L. S. Fully Bridged Triphenylamine Derivatives as Color-Tunable Thermally Activated Delayed Fluorescence Emitters. *Org. Lett.* **2021**, *23* (3), 958-962.

(13) Qiu, X.; Tian, G.; Lin, C.; Pan, Y.; Ye, X.; Wang, B.; Ma, D.; Hu, D.; Luo, Y.; Ma, Y. Narrowband Emission from Organic Fluorescent Emitters with Dominant Low-Frequency Vibronic Coupling. *Adv. Opt. Mater.* **2021**, *9* (4), 2001845.

(14) Min, H.; Park, I. S.; Yasuda, T. cis-Quinacridone-Based Delayed Fluorescence Emitters: Seemingly Old but Renewed Functional Luminogens. *Angew. Chem. Int. Ed.* **2021**, *60* (14), 7643-7648.

(15) Fan, X. C.; Wang, K.; Shi, Y. Z.; Chen, J. X.; Huang, F.; Wang, H.; Hu, Y. N.; Tsuchiya, Y.; Ou, X. M.; Yu, J. Managing Intersegmental Charge-Transfer and Multiple Resonance Alignments of D3-A Typed TADF Emitters for Red OLEDs with Improved Efficiency and Color Purity. *Adv. Opt. Mater.* **2022**, *10* (3), 2101789.

(16) Lampe, T.; Schmidt, T. D.; Jurow, M. J.; Djurovich, P. I.; Thompson, M. E.; Brütting, W. Dependence of Phosphorescent Emitter Orientation on Deposition Technique in Doped Organic Films. *Chem. Mater.* **2016**, *28* (3), 712-715.
